# Robust excision of different purine lesions by homologs thymine DNA glycosylase and mismatch-specific uracil DNA-glycosylase

**DOI:** 10.1016/j.jbc.2026.113446

**Published:** 2026-08-13

**Authors:** Kurt B. Espinosa, Xiao Ding, Hardler W. Servius, Lakshmi S. Pidugu, Jeehiun K. Lee, Alexander C. Drohat

**Affiliations:** 1 Department of Biochemistry and Molecular Biology, University of Maryland School of Medicine, Baltimore, Maryland, USA; 2 Department of Chemistry and Chemical Biology, Rutgers, The State University of New Jersey, New Brunswick, New Jersey, USA; 3 Cancer Biology Program, University of Maryland Marlene and Stewart Greenebaum Comprehensive Cancer Center, Baltimore, Maryland, USA

**Keywords:** Base excision repair, DNA damage, DNA repair, DNA glycosylase, 7,8-dihydro-8-oxoadenine, xanthine, oxidation, deamination, UDG superfamily

## Abstract

DNA bases are subject to alkylation, deamination, and oxidation, yielding mutagenic and cytotoxic lesions implicated in cancer and neurodegenerative disease. The damage is countered by DNA glycosylases, which hydrolyze the *N*-glycosyl bond to release the lesion and initiate base excision repair. One glycosylase family is represented by human thymine DNA glycosylase (TDG) and *Escherichia coli* mismatch-specific uracil DNA-glycosylase (MUG), which exhibits 32% sequence identity. TDG excises thymine/uracil from guanine mispairs, 5-formylcytosine, 5-carboxylcytosine, and 3,*N*^4^-ethenocytosine, while MUG excises 3,*N*^4^-ethenocytosine and mismatched uracil. Remarkably, TDG was shown to excise a purine lesion, 7,8-dihydro-8-oxoadenine (8OA), much faster than pyrimidine substrates, while MUG lacks 8OA activity. To define the purine specificity of these enzymes, we performed structure-activity relationship studies, determining the rate constant for excision of 10 different purines from DNA. We also performed theoretical calculations to determine chemical properties of the purines that could influence their enzymatic excision, including N9 acidity, an indicator of leaving group ability during bond cleavage, tautomer stability, and electrostatic potential maps. TDG activity is 4 to 7 orders of magnitude higher for 8OA *versus* other purines, while MUG exhibits strikingly high activity and specificity for excision of xanthine. Rate enhancements for TDG excision of 8OA (10^9.2^), other purines (≤10^4.5^), uracil (10^8.2^), and thymine (10^7.1^) reveal high specificity for 8OA, and those of MUG for excising xanthine (10^7.8^), other purines (≤10^5.2^), and uracil (10^7.8^), indicate high xanthine specificity. The results suggest mechanisms for purine specificity and provide evidence that 8OA and xanthine are biological substrates of TDG and MUG, respectively.

The bases of DNA are subjected to chemical modification through reactions involving alkylation, deamination, and oxidation, yielding a wide range of mutagenic and cytotoxic lesions that are implicated in cancer, neurodegenerative diseases, and in aging (1). Damaged DNA bases are corrected predominantly through base excision repair (BER), a conserved pathway that is essential for maintaining genomic integrity and is present in all three domains of life (2, 3). BER is initiated by DNA glycosylases, which employ a nucleotide flipping mechanism to identify modified bases and excise them by hydrolyzing the *N*-glycosyl bond, generating an abasic site (4, 5), and the process is completed by follow-on BER enzymes. Some DNA glycosylases are highly specific, excising a single or limited number of damaged bases, as exemplified by MutY excision of adenine mispaired with 7,8-dihydro-8-oxoguanine (8OG) (6), and uracil DNA glycosylase (UNG) excision of uracil (Fig. 1) (7). By contrast, other DNA glycosylases can excise a broad range of base lesions, as illustrated by alkyladenine DNA glycosylase (AAG) excision of the adenine lesions hypoxanthine (H) and 1,*N*^6^-ethenoadenine, among other types of damage (8).

**Figure 1:**
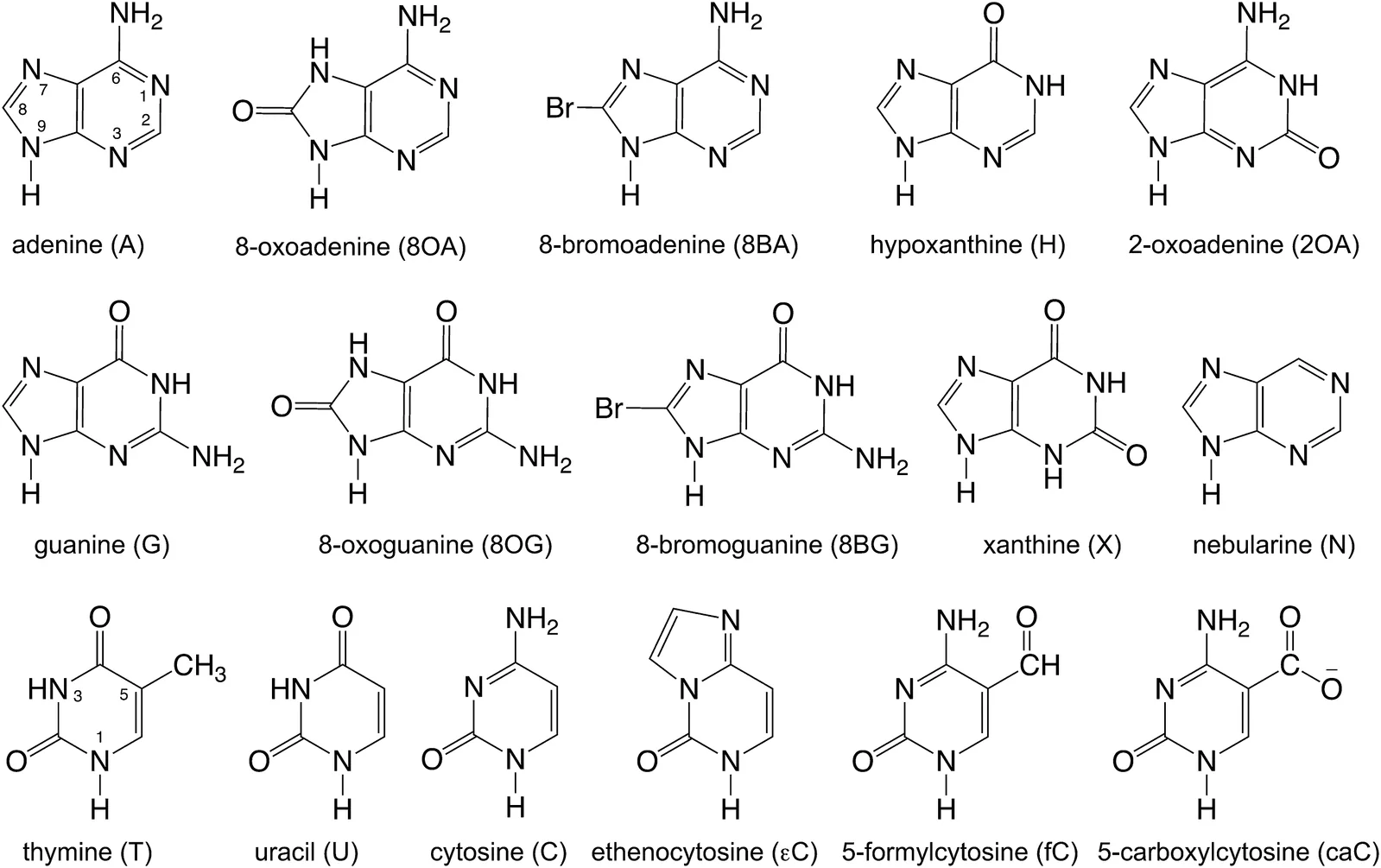
**Structures of purine and pyrimidine bases considered in this work**.

Another example of a DNA glycosylase with broad substrate specificity is thymine DNA glycosylase (TDG), which was discovered for its ability to excise thymine from mutagenic G⋅T mispairs that can arise by deamination of 5-methylcytosine (mC) to thymine (9, 10). TDG also excises uracil, 5-halogenated uracils, 5-hydroxymethyl uracil, and the cytosine lesion 3,*N*^4^-ethenocytosine (εC), with a preference for removing these bases when they are paired with guanine in duplex DNA (11, 12, 13, 14, 15). Moreover, TDG performs a critical role in the epigenetic process of active DNA demethylation by excising 5-formylcytosine (fC) and 5-carboxylcytosine (caC), which are generated *via* oxidation of mC by ten-eleven translocation (TET) enzymes (16, 17, 18). The breadth of substrate specificity exhibited by TDG was expanded to include purines with the finding that it excises the major adenine oxidative lesion, 7,8-dihydro-8-oxoadenine (8OA) from DNA *in vitro* and is required for 8OA excision in murine cell free extracts (19). We subsequently defined the magnitude of this activity *in vitro*, finding that TDG excises 8OA much (18- to 480-fold) faster than the pyrimidines (when bases are paired with G) and excises 8OA from T⋅8OA pairs with activity that is comparable to that of the pyrimidine substrates (20, 21). 8OA is generated by ionizing irradiation and other oxidizing agents, is readily detected in normal mammalian cells, and is found at elevated levels in cancer cells (22, 23, 24, 25, 26, 27, 28, 29). 8OA is mutagenic when encountered by mammalian DNA polymerases, where Pol α, Pol β, and Pol η, incorporate dGTP opposite 8OA, causing A→C transversions, and Pol β incorporates dCTP opposite 8OA, causing A→G transitions (30, 31, 32, 33, 34, 35, 36). Moreover, 8OA forms interstrand crosslinks with A or G under oxidative conditions *in vitro* (37), and it perturbs the activity of enzymes including RNA polymerase II (38), the exonuclease activity of the WRN protein (39, 40), and the repair of proximal abasic sites by AP endonuclease 1 and bifunctional DNA glycosylases (41). While TDG is the only human DNA glycosylase with substantial 8OA activity, the mechanism by which it recognizes and excises this mutagenic and cytotoxic lesion remains unknown.

Early studies of TDG led to the discovery of a homolog in *Escherichia coli* that has activity for G⋅U mispairs, termed mismatch-specific uracil DNA-glycosylase (MUG), and related enzymes in insects, unveiling TDG-MUG as a major family within the UNG superfamily (42, 43). While *E. coli* MUG and human TDG (catalytic domain) exhibit 32% sequence identity and a similar overall fold, they have variations in several active site residues and exhibit starkly different activity for some base lesions (13, 20, 44, 45, 46, 47). Relative to TDG, MUG activity is 10^5^-fold lower for 8OA and 100-fold higher for εC, and MUG lacks significant activity for G⋅T mispairs. MUG excises 1,*N*^2^-ethenoguanine (48) and xanthine (X), which arises *via* guanine deamination, and MUG is required for excision of X in *E. coli* cell extracts (49). Notably, guanine deamination can occur *via* spontaneous hydrolysis or nitrosylation by N_2_O_3_ generated from nitric oxide, which is released by activated macrophages in the host immune response (50, 51). Guanine deamination yields X·C pairs initially, and subsequent DNA replication gives X·T mispairs that cause G → A transition mutations (52, 53). MUG appears to be the predominant DNA glycosylase for excision of xanthine in *E*. *coli* (49), but the mechanism by which it recognizes and removes this mutagenic lesion remains unknown.

Regarding what is known about the mechanism of base excision by DNA glycosylases, prior studies show that *N*-glycosyl bond hydrolysis for deoxynucleotides, either spontaneous or enzyme-catalyzed, proceeds through one of two general mechanisms (Fig. 2*A*). These include a stepwise (S_N_1) mechanism, where rupture of the *N*-glycosyl bond (step 1) gives a discrete oxacarbenium-ion intermediate followed by nucleophile (water) addition (step 2), and a concerted (S_N_2) mechanism that has a single oxacarbenium ion-like transition state (TS) in which LG departure is advanced over nucleophile addition (a “loose” TS) (54, 55, 56, 57, 58, 59). Each pathway features a TS in which the *N*-glycosyl bond is largely ruptured, with positive charge developing on the ribose and negative charge migrating to the LG. The inherent LG ability of a given nucleobase depends on its capacity to accommodate the incoming negative charge, as indicated by the acidity (p*K*_a_) of the glycosidic nitrogen (N1 pyrimidines and N9 purines), where LG ability increases with acidity (decreasing p*K*_a_) (Fig. 2*B*) (60). The rate constant (*k*) for spontaneous reactions depends on LG ability (p*K*_a_), as observed for a series of deoxyuridines with p*K*_a_-modulating C5-substituents and for other deoxynucleotides as reported previously and shown below (14, 61, 62, 63). Notably, LG ability is enhanced by alkylation when it renders a positive charge on the base (*e.g.*, 3-methyladenine) or by protonation of the base (acid catalysis), which both serve to stabilize negative charge arising from bond cleavage (Fig. 2*C*).

**Figure 2:**
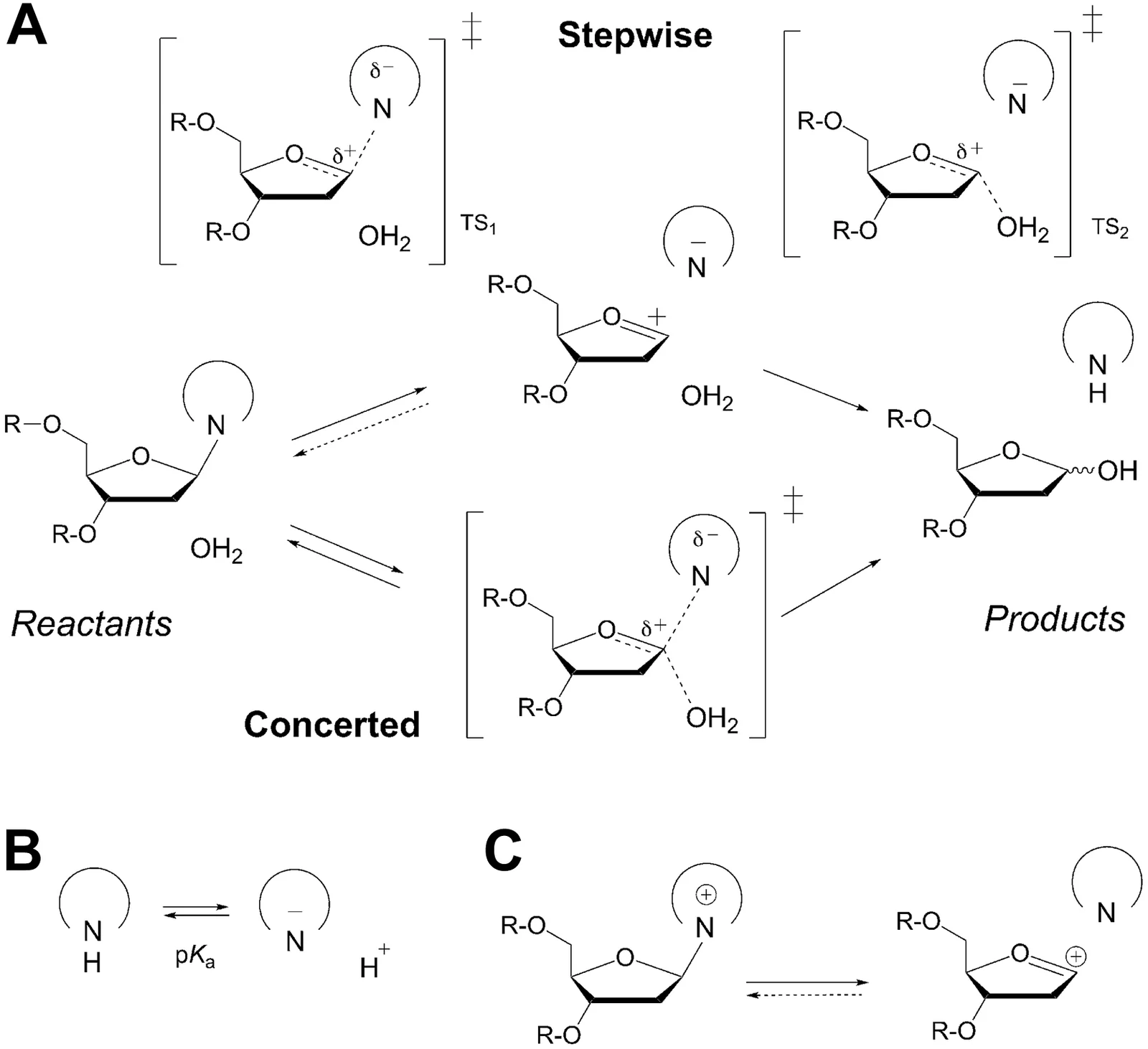
**Mechanisms for *N*-glycosyl bond hydrolysis.***A*, two general mechanisms for hydrolysis of the *N*-glycosyl bond in deoxynucleotides. A stepwise (S_N_1) reaction features atransition state (TS) for each of two steps, including leaving-group departure and nucleophile addition, while a concerted (S_N_2) mechanism has a single TS. *B*, acidity of the glycosidic nitrogen for the free nucleobase provides a measure of inherent LG ability. *C*, leaving group ability of a nucleobase is enhanced by protonation of the base, typically at a ring nitrogen.

DNA glycosylases catalyze *N*-glycosyl bond hydrolysis in part through leaving group activation, which can involve electrostatic interactions that stabilize negative charge on the departing nucleobase in the TS for bond cleavage, or protonation of the base (*i.e.*, general or specific acid catalysis) (60). Studying the dependence of glycosylase activity on nucleobase LG ability can be complicated by enzyme interactions that can impart differing effects on LG ability, or nucleotide flipping, among a group of potential nucleotide substrates. Nevertheless, such studies can provide mechanistic insight. For example, TDG activity for excising C5-substituted uracils and cytosines was found to depend on N1 p*K*_a_ for a group of C5 substituents that modulate N1 acidity without dramatically altering active site interactions, indicating that specificity for excising U or T from guanine mispairs but not C (from G·C pairs) depends on LG ability (N1 p*K*_a_) (14, 64). Other studies indicate that TDG specificity for excising fC and caC likely depends on the greater LG ability (N1 acidity) of these bases relative to C, mC, and 5-hydroxymethyl-C (64), though specificity could also derive from enzyme–base interactions that may stabilize nucleotide flipping to a greater extent for fC and caC *versus* the other cytosines (65, 66, 67). In another example, *E*. *coli* AlkA exhibits nearly the same rate enhancement for excision of six diverse purine bases, indicating that activity depends on the inherent stability of the *N*-glycosyl bond and, to a large extent, on LG ability (N9 acidity) of the purine base (59, 61, 68, 69). These examples illustrate that the specificity of DNA glycosylases that excise a diverse group of nucleobases can depend in part on the inherent LG ability of those nucleobases.

In the studies reported below, we investigated the mechanisms by which TDG and MUG attain specificity for excising select purines from DNA. We performed structure-activity relationship studies, determining the activity of TDG and MUG for excising 10 different purine bases (Fig. 1). We also performed theoretical calculations to determine chemical properties of the purines that could influence their enzymatic excision, including N9 acidity, electrostatic potential maps, and the relative stability and N9 acidity of tautomers for 8OA and xanthine. We address the question of whether the activity of TDG and MUG depends on inherent LG ability (N9 acidity) for the 10 purine bases and consider the rate enhancement exhibited by the enzymes for excision of purine and pyrimidine substrates. Together, the results advance our understanding of the mechanism for selective purine excision by TDG and MUG, underscore the vast differences in specificity for these homologous enzymes, and inform the role of these enzymes in protecting against mutagenic and cytotoxic purine lesions in cells.

## Results

### Experimental considerations

The catalytic turnover of both TDG and MUG is strongly limited by slow release of the reaction product, AP (apurinic/apyrimidinic) DNA, and potent product inhibition, such that rate constants obtained from multiple turnover kinetics experiments (*k*_cat_) are dominated by these events and not useful for comparing activity between different substrates or enzymes (44, 70). As such, glycosylase activity was determined using single turnover kinetics experiments performed under saturating enzyme conditions, giving rate constants (*k*_obs_) that approximate the maximal rate of base excision (*k*_max_) and are not impacted by events after the chemical step (*N*-glycosyl bond hydrolysis) (44, 71). Notably, prior studies report *k*_max_/*k*_cat_ ratios of 40- to 440-fold for TDG and MUG acting on various substrates, demonstrating the dramatic impact of slow product release on *k*_cat_ (44, 70, 72). While single turnover kinetics experiments are the most appropriate method for the studies here, the observed rate constants (*k*_obs_) do not necessarily reflect cellular activity, which can be influenced by many factors including the concentration and localization of the glycosylase, the accessibility of lesions in genomic DNA, the dissociation rate of the glycosylase from its abasic product and rebinding to that product, and stimulation of product release by an AP endonuclease or other protein (21, 44, 70, 73, 74). Nevertheless, because product release is much slower than base excision for TDG and MUG (*k*_cat_ << *k*_max_), and product release can be dramatically enhanced by cellular factors including AP endonucleases (such that *k*_cat_ can approach *k*_max_), the results from single turnover experiments (*k*_max_) are likely to be more informative than those from multiple turnover experiments (*k*_cat_) regarding cellular activity. We note that the kinetics experiments were collected at 23 °C because prior studies find that MUG is not sufficiently stable over extended time periods at 37 °C (44).

Regarding the DNA substrates, prior studies show that TDG excision of target bases (*e.g.*, T, U, 8OA) is fastest when the base is paired with G *versus* A (15, 20, 75), and MUG excision of U is much higher for G⋅U *versus* A⋅U pairs (47). As such, the duplex DNA substrates (28 bp) include a “target” base that is paired with G in the complementary strand (Fig. S1). Previous work has also shown that TDG activity for excising a given base depends on its 3′ neighbor, with optimal activity for G at the 3′ position, particularly for excision of T (from G·T pairs) but also for excision of U and 8OA (13, 15, 21, 76, 77). These findings reflect the strong specificity of TDG for excising deaminated mC from a DNA context for which cytosine methylation is most abundant (CpG dinucleotides). As such, the DNA substrates used here contain G at the 3′ position relative to the target base.

### Excision of modified purines by TDG

**Figure 3:**
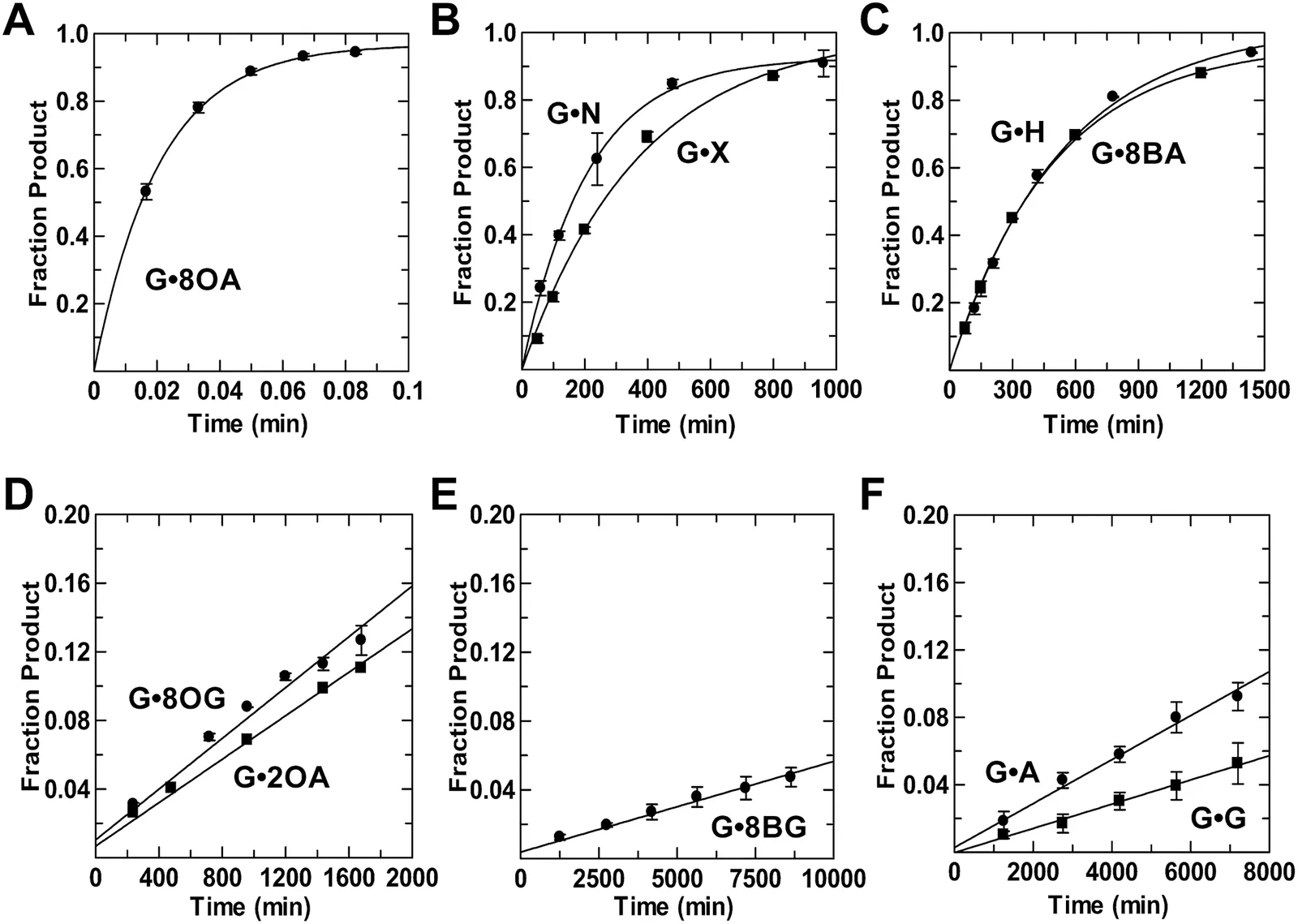
**Activity of TDG for excising damaged purines.** Results from single turnover kinetics experiments for TDG activity on DNA substrates containing base pairs including (*A*) G⋅8OA, (*B*) G⋅N (*circles*) and G⋅X (*squares*), (*C*) G⋅H (*circles*) and G⋅8BA (*squares*), (*D*) G⋅8OG (*circles*) and G⋅2OA (*squares*), (*E*) G⋅8BG, and (*F*) G⋅A (*circles*) and G⋅G (*squares*). Progress curves were fitted with a single exponential equation or a linear equation, giving rate constants shown in Table 1. Values are the mean ± SD for at least three replicates. The experiments were collected at 23 °C using a DNA substrate concentration of 0.5 μM and a TDG concentration of 2.5 μM or 5.0 μM. 8OA, 7,8-dihydro-8-oxoadenine; TDG, thymine DNA glycosylase.

To investigate the mechanism and specificity for excision of 8OA by TDG, we determined its activity for removing a wide range of other modified purines from duplex DNA (Fig. 3, Table 1). As we reported previously, TDG activity for excising 8OA from G⋅8OA pairs, *k*_obs_ = 48.7 ± 0.9 min^−1^, greatly exceeds that for its established pyrimidine substrates including G⋅T, G⋅U, G⋅fC, G⋅caC, and G⋅εC (20, 21). By contrast, we find that TDG activity for removing nine other purine bases is reduced by at least four orders of magnitude relative to 8OA and is also much lower than activity for the pyrimidine substrates. Thus, TDG activity for excising xanthine (X), hypoxanthine (H), 8-bromoadenine (8BA), and nebularine (N), is reduced by 10,000- to 25,000-fold compared to that for 8OA. TDG activity is even further reduced for other purines, where excision is 800,000- and 9 million-fold slower for 8OG and 8-bromoguanine (8BG), respectively, relative to 8OA. TDG activity is also extremely low for excision of 2-oxoadenine (2OA) and the unmodified purines A and G (from G⋅A and G⋅G mispairs).

**Table 1:** Glycosylase activity of TDG and MUG

Substrate base	TDG *k*_obs_ (min^−1^)	TDG *k*_obs_ relative to 8OA	MUG *k*_obs_ (min^−1^)	MUG *k*_obs_ relative to X	Nonenzymatic reaction *k*_non_ (min^−1^)^a^	TDG rate enhancement^b^	MUG rate enhancement^b^
8-oxoadenine (8OA)	48.7 ± 0.9	(1)	(2.7 ± 0.1) × 10^–4^	10^–5.3^	2.9 × 10^–8^^c^	10^9.2^	10^4.0^
8-bromoadenine (8BA)	(2.0 ± 0.1) × 10^–3^	10^–4.4^	(3.0 ± 0.1) × 10^–3^	10^–4.3^			
hypoxanthine (H)	(1.9 ± 0.1) × 10^–3^	10^–4.4^	0.010 ± 0.001	10^–3.7^	6.6 × 10^–8^	10^4.5^	10^5.2^
2-oxoadenine (2OA)	(6.0 ± 0.1) × 10^–5^	10^–5.9^	(3.1 ± 0.1) × 10^–5^	10^–6.2^			
adenine (A)	(1.3 ± 0.1) × 10^–5^	10^–6.6^	(2.2 ± 0.1) × 10^–5^	10^–6.4^	7.2 × 10^–9^	10^3.3^	10^3.5^
8-oxoguanine (8OG)	(6.4 ± 0.2) × 10^–5^	10^–5.9^	(5.2 ± 0.2) × 10^–4^	10^–5.0^			
8-bromoguanine (8BG)	(5.0 ± 0.4) × 10^–6^	10^–7.0^	≤ 1.4 × 10^–6^^d^	≥10^–7.6^			
xanthine (X)	(2.7 ± 0.2) × 10^–3^	10^–4.3^	55.0 ± 1.3	(1)	9.3 × 10^–7^^e^	10^3.5^	10^7.8^
nebularine (N)	(4.8 ± 0.2) × 10^–3^	10^–4.0^	(4.6 ± 0.1) × 10^–5^	10^–6.1^			
guanine (G)	(7.2 ± 0.6) × 10^–6^	10^–6.8^	(4.5 ± 0.1) × 10^–5^	10^–6.1^	1.9 × 10^–8^	10^2.6^	10^3.4^
uracil (U)	2.13 ± 0.07	10^–1.4^	0.97 ± 0.05	10^–1.8^	1.5 × 10^–8^	10^8.2^	10^7.8^
thymine (T)	0.158 ± 0.002	10^–2.5^	(9.7 ± 0.3) × 10^–5^	10^–5.8^	1.4 × 10^–8^	10^7.1^	10^3.8^
ethenocytosine (εC)	0.060 ± 0.001	10^–2.9^	8.99 ± 0.46	10^–0.8^			
cytosine (C)	(1.2 ± 0.5) × 10^–5^^f^	10^–6.6^	ND		1.4 × 10^–8^	10^3.3^	

a Rate constant (*k*_non_) for spontaneous hydrolysis of dA, dG, deoxyinosine (H), dU, and thymidine at 25 °C as reported in Ref (78).

b Rate enhancement is given by the ratio of rate constants for the enzyme catalyzed and spontaneous reactions (*k*_obs_/*k*_non_). Values are slightly below actual because enzyme activity will be slightly higher at 25 °C *versus* 23 °C (experimental temperature). Rate enhancements for TDG excision of C, U, and T are lower than we previously reported (Ref (14)), because *k*_non_ values used here (Wolfenden, Ref (78)) are higher than those used previously.

c *k*_non_ was obtained from the linear dependence of log *k*_non_ on N9 p*K*_a_ for dA, dG, dI, and dX (log *k*_non_ = −0.68(p*K*_a_) −1.08; *r*^2^ = 0.982).

d Activity was not detected so value shown is an upper limit for activity assuming <1% product in 5 days (or 7200 min).

e Value for *k*_non_ reported in Ref (79) for hydrolysis of dX at 37 °C, pH 6 was converted to *k*_non_ at 25 °C using the average of the ratio for *k*_non_ at these temperatures (*k*_non_^37^/*k*_non_^25^ = 5.5) observed for dA, dG, and dI in Ref (78), and reduced two-fold to adjust for pH (from pH 6.0 to pH 6.8).

f Value from Ref. (14).

We also calculated the rate enhancement (or catalytic power) for excision of 8OA and other purines by TDG, which is given by the ratio of rate constants for maximal enzyme activity and the nonenzymatic reaction (*k*_max_/*k*_non_) (Table 1). Prior studies reported *k*_non_ for spontaneous hydrolysis of four purine deoxynucleosides considered here, including dA, dG, dI, 2′-deoxyxanthosine (dX), and for the pyrimidines dC, dU, and thymidine (78, 79). Notably, we find a linear dependence of log *k*_non_ on N9 p*K*_a_ for the four purine deoxynucleosides (*r*^2^ = 0.982; Table 1, legend), and this relationship, together with the N9 p*K*_a_ for 8OA, was used to estimate *k*_non_ for 8-oxo-dA. The result indicates that TDG exbibits a rate enhancement of 10^9.2^ for excision of 8OA from G⋅8OA pairs, corresponding to 12.5 kcal/mol of TS stabilization relative to the spontaneous reaction. Notably, the rate enhancement for 8OA is much (≥10^4.7^-fold) higher than that for four other purines and higher than that for excision of uracil (10^8.2^) and thymine (10^7.1^). These findings reveal that TDG exhibits remarkably high specificity for excising 8OA *versus* other purine lesions.

### Excision of modified purines by MUG

**Figure 4:**
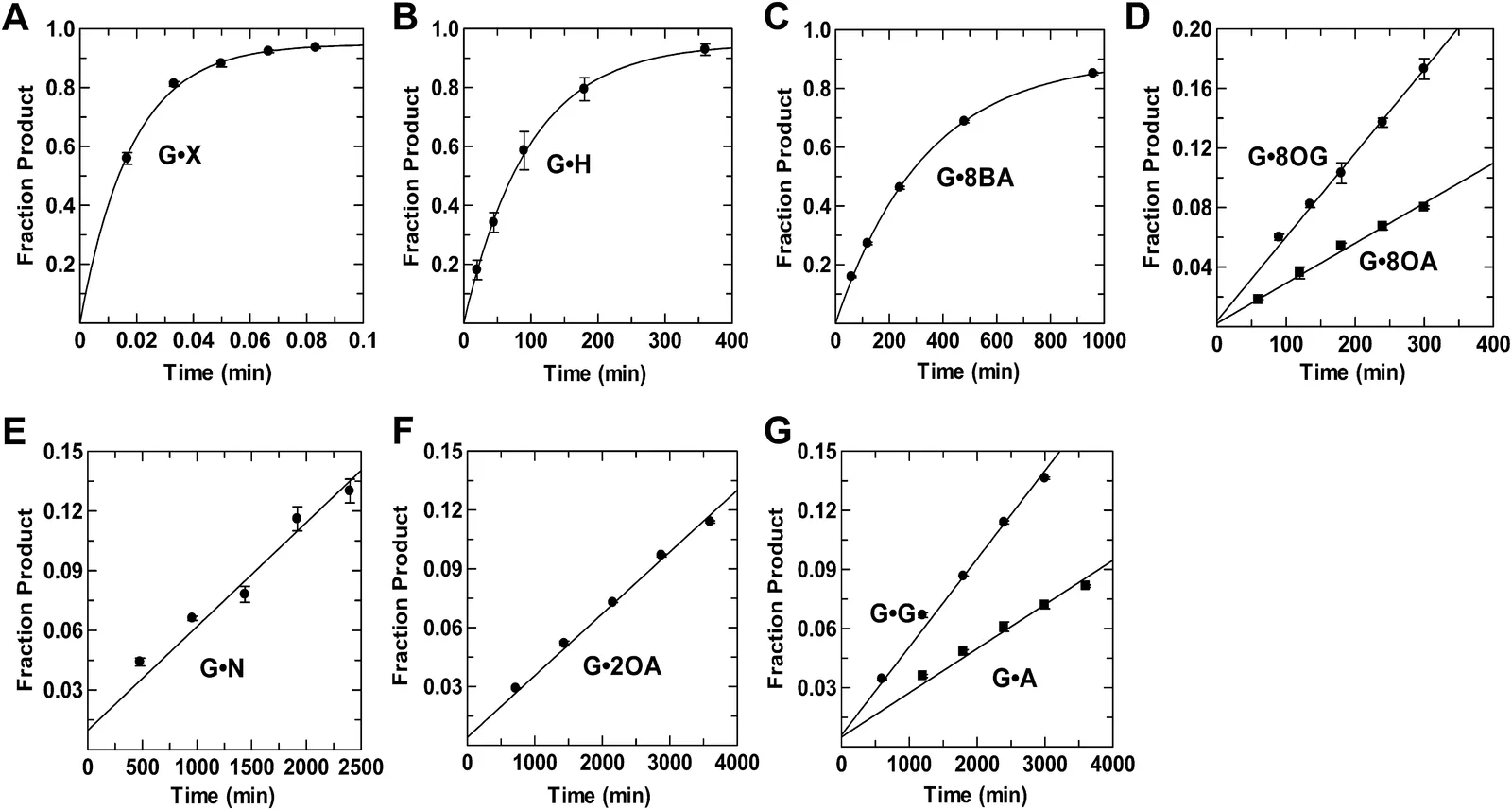
**Activity of MUG for excising damaged purines.** Results from single turnover kinetics experiments for MUG activity on DNA substrates containing base pairs including (*A*) G⋅X, (*B*) G⋅H, (*C*) G⋅8BA, (*D*) G⋅8OG (*circles*) and G⋅8OA (*squares*), (*E*) G⋅N, (*F*) G⋅2OA, and (*G*) G⋅G (*circles*) and G⋅A (*squares*). Progress curves were fitted with a single exponential equation or a linear equation, and the resulting rate constants are provided in Table 1. Values are the mean ± SD for at least three replicates. The experiments were collected at 23 °C using a DNA substrate concentration of 0.5 μM and a MUG concentration of 10 μM. 2OA, 2-oxoadenine; 8BA, 8-bromoadenine; 8OA, 7,8-dihydro-8-oxoadenine; 8OG, 7,8-dihydro-8-oxoguanine; MUG, mismatch-specific uracil DNA-glycosylase.

We also determined the activity of MUG for acting on the same set of purine substrates (Fig. 4, Table 1). MUG exhibits strikingly high activity for excision of xanthine, with a rate constant, *k*_obs_ = 55.0 ± 1.3 min^−1^, that is 57-fold higher than its activity for G⋅U pairs, the pyrimidine substrate for which it was discovered, and 6-fold greater than its robust activity for G⋅εC pairs. Moreover, MUG exhibits very high specificity for xanthine over the nine other purines examined here. For example, MUG activity for H is 5300-fold lower than X, and activity for 8BA and 8OG is reduced by 18,000- and 100,000-fold relative to X. MUG activity is reduced by 200,000-fold for 8OA relative xanthine, and its 8OA activity is five orders of magnitude below that of TDG (67). MUG activity is reduced by at least six orders of magnitude for N and 2OA relative to X and it does not detectably excise 8BG over a period of 5 days, indicating an upper limit of *k*_obs_ ≤1.4 × 10^−6^ min^−1^ (assuming fraction product <0.01 in 5 days) and an activity reduction of at least 40 million-fold relative to X. Our results show that MUG exbibits a rate enhancement of 10^7.8^ for excision of X from G⋅X pairs, corresponding to 10.6 kcal/mol of TS stabilization relative to the spontaneous reaction. This rate enhancement is equivalent to that that for uracil excision and much (>400-fold) higher than that for excision of other purines. Thus, MUG exhibits high specificity for excising X *versus* other purine lesions.

### Theoretical calculations for purines and pyrimidines

**Figure 5:**
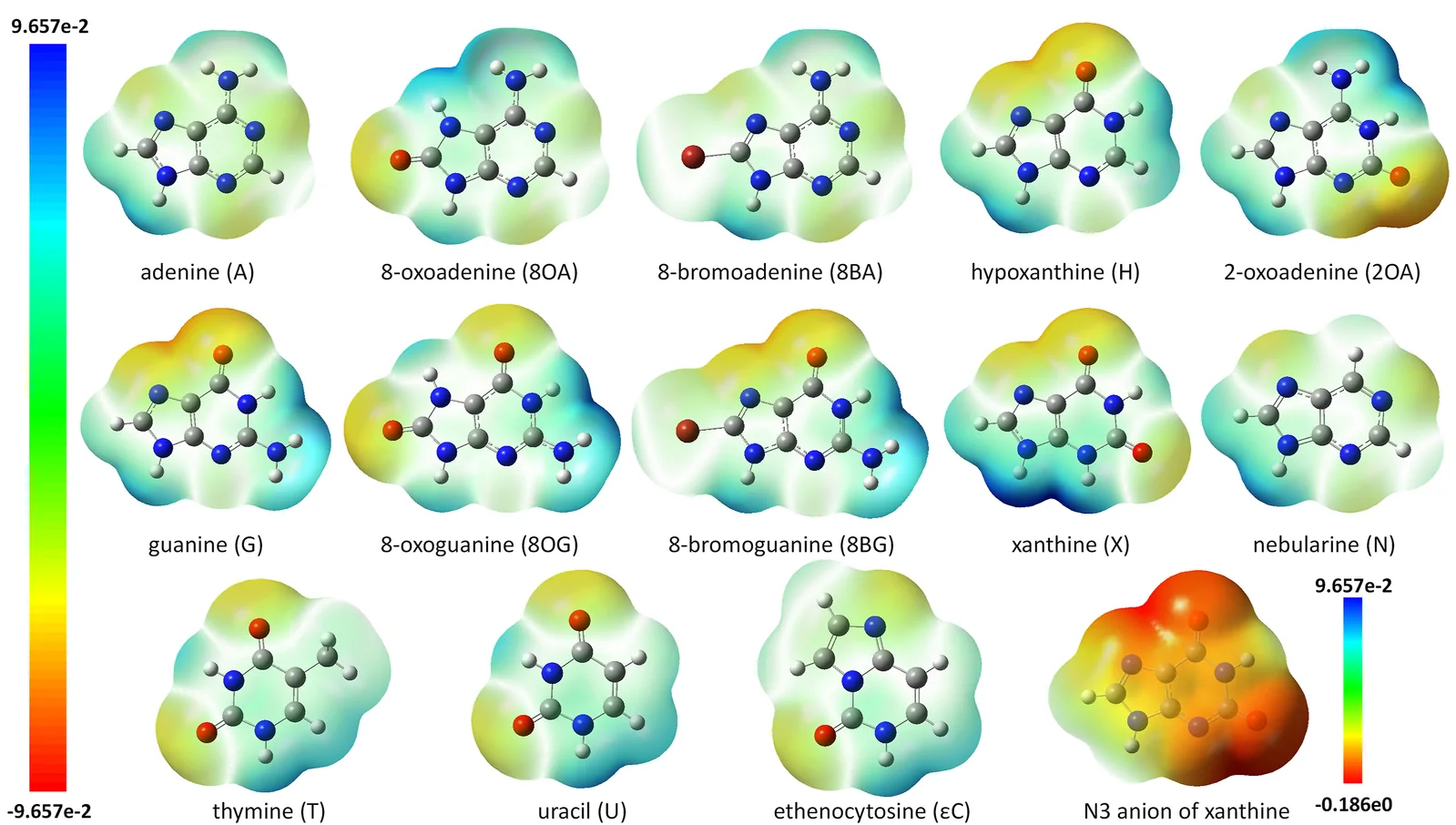
**Electrostatic potential maps of neutral purines and pyrimidines and N3 anion of X.** The range of electrostatic potential varies from −0.097 (*red*) to 0.097 (*blue*) for the neutral bases and from −0.19 (*red*) to 0.097 (*blue*) for the N3 anion of xanthine.

To inform the mechanism for rapid and selective excision of 8OA and xanthine by TDG and MUG, respectively, we calculated the N9 acidity for these and eight other purines, using the B3LYP-D3(BJ)/6-311++G (2d,p) basis set (Table 2). We also calculated the electrostatic potential maps for the purines and several pyrimidines (Fig. 5). The acidities are reported as the free energy of N9-H deprotonation (Δ*G*, kcal/mol), in the gas phase and in water, where a lower value indicates higher acidity and better inherent LG ability during *N*-glycosyl bond hydrolysis. We find that xanthine exhibits the highest N9 acidity in both the gas phase and water, while 8OA has the lowest acidity in the gas phase and among the lowest in water. The gas phase acidities decrease with a trend of X >> 8BG > 8BA >> N > H >> 2OA > G > A > 8OG > 8OA, while acidities in water decrease as X > 8BG > 8BA > H > N > 8OG > 8OA > G > A > 2OA. Table 2 also includes N9 p*K*_a_ values, calculated and experimental, which have been previously reported for each of the 10 purines considered here except 8OA, 8BA, and 8BG (to our knowledge) (62, 80, 81, 82, 83). We find a strong correlation between the calculated N9 acidity in water and N9 p*K*_a_ (*r*^2^ = 0.988; Fig. S2*A*) and a good correlation between calculated gas phase N9 acidity and N9 p*K*_a_ (*r*^2^ = 0.845; Fig. S2*B*). As such, we used the fitting for N9 acidity in water to predict N9 p*K*_a_ values of 9.5 for 8OA, 7.6 for 8BA, and 7.4 for 8BG (Table 2).

**Table 2:** Calculated acidity of purines (N9-H) and pyrimidines (N1-H) and reported p*K*_a_

Base	Acidity, gas phase (Δ*G*, kcal/mol)^a^	Acidity, water (Δ*G*, kcal/mol)^a^	p*K*_a_
purines			
xanthine (X)	312.4	11.4	7.3^b^
8-bromoguanine (8BG)	321.3 (8.9)	11.8 (0.4)	7.4^c^
8-bromoadenine (8BA)	321.9 (9.4)	12.2 (0.8)	7.6^c^
nebularine (N)	325.6 (13.2)	15.4 (4.1)	8.9^d^
hypoxanthine (H)	326.3 (13.8)	15.3 (3.9)	8.9^e^
2-oxoadenine (2OA)	329.9 (17.5)	19.0 (7.6)	10.6^b^
guanine (G)	330.1 (17.7)	18.0 (6.7)	10.0^b^
adenine (A)	330.8 (18.3)	18.3 (6.9)	10.2^b^
8-oxoguanine (8OG)	330.9 (18.5)	16.0 (4.6)	9.5^f^
8-oxoadenine (8OA)	331.2 (18.7)	16.6 (5.2)	9.5^c^
pyrimidines			
uracil (U)	326.5	15.1	9.8^g^
thymine (T)	328.2	16.2	10.2^g^
ethenocytosine (εC)	327.5	16.3	

a Values in parentheses are the difference in acidity (ΔΔ*G*) relative to xanthine; values were calculated at B3LYP-D3(BJ)/6-311++G (2d, p). Previously reported p*K*_a_ values (calculated and experimental) are from.

b Ref. (80).

c p*K*_a_ values for 8BA, 8BG, and 8OA are estimates obtained by interpolation from the linear dependence of calculated N9 acidities (water) on N9 p*K*_a_ for the seven other purines (Fig. S2*A*, see main text).

d Ref. (80).

e Ref. (83).

f Ref. (82).

g Ref. (62).

### Stability and N9 acidity for tautomeric forms of 8-oxoadenine

**Figure 6:**
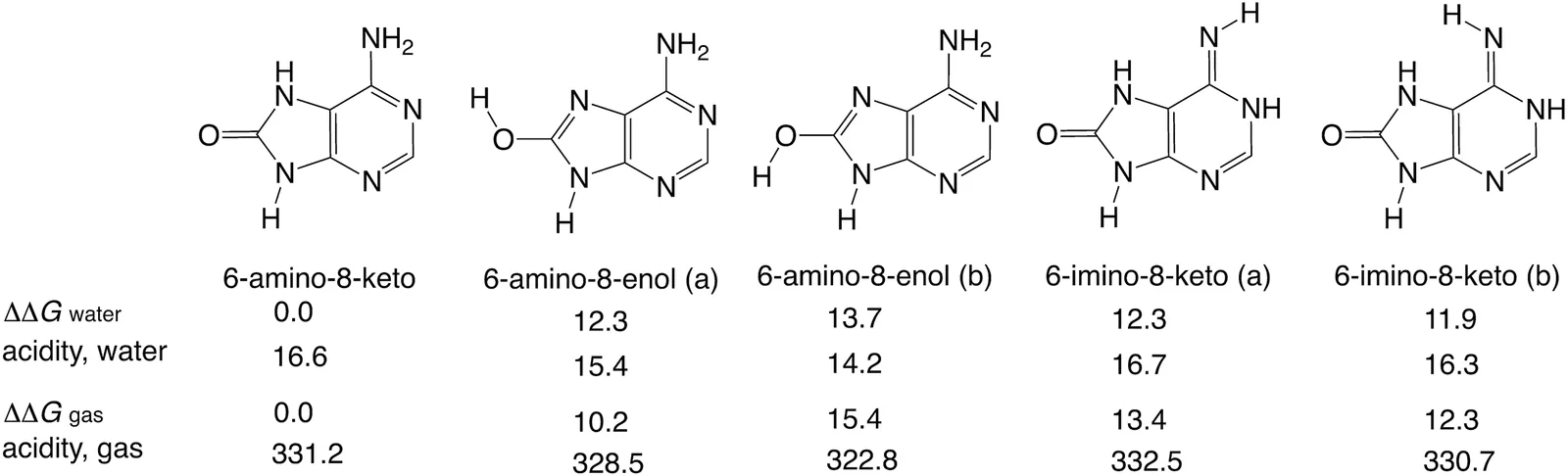
**Stability and N9 acidity of 8-oxoadenine N9-H tautomers.** Calculated stability of N9-H 8OA tautomers, in water and the gas phase, shown as the difference in free energy (ΔΔ*G*, kcal mol^–1^) with respect to the most stable species (ΔΔ*G* = 0). Also shown are calculated N9 acidities. 8OA, 7,8-dihydro-8-oxoadenine.

While the 8-oxoadenine lesion was initially thought to exist as a 6-amino-8-enol tautomer termed 8-hydroxyadenine (Fig. 6), NMR studies of the free deoxynucleoside and the deoxynucleotide in duplex DNA show that the 8-keto tautomer predominates in aqueous solution (84, 85). However, the relative stability of these and the full set of other N9-H tautomers of 8OA have not been reported (to our knowledge) (86). Obtaining this information is important given that tautomerization of 8OA, even transiently, could affect its excision by a DNA glycosylase or alter its base pairing properties and thereby cause a DNA polymerase to generate mutagenic mispairs when the lesion arises in the template strand or in the nucleotide pool (as 8-oxo-dNTP). For example, prior studies suggest that TDG excision of caC could involve protonation of its carboxylate to give the neutral amino species, followed by tautomerization to the imino form of caC prior to *N*-glycosyl bond cleavage (66). As such, we calculated the relative stability of N9-H tautomers for 8OA and find that the 6-amino-8-keto form is the most stable by a large margin (ΔΔ*G* ≥ 12 kcal/mol in water and >10 kcal/mol in the gas phase) (Figs. 6 and S3). We also calculated the N9 acidity of four tautomers exhibiting stability within 20 kcal/mol of the 6-amino-8-keto species and find that the 6-amino-8-enol tautomers exhibit modestly higher acidity than the 6-amino-8-keto. While this raises the possibility that TDG could mediate a change in tautomeric state to facilitate 8OA excision, the greatly reduced stability of the 6-amino-8-enol tautomers would pose a large energetic barrier, arguing against such a mechanism. The two most stable of four possible 6-imino-8-keto species exhibit lower N9 acidity and much lower stability relative to the 6-amino-8-keto tautomer and are therefore unlikely to play a role in TDG excision of 8-oxoadenine.

### Ionization of xanthine N3 and its effect on N9 acidity

**Figure 7:**
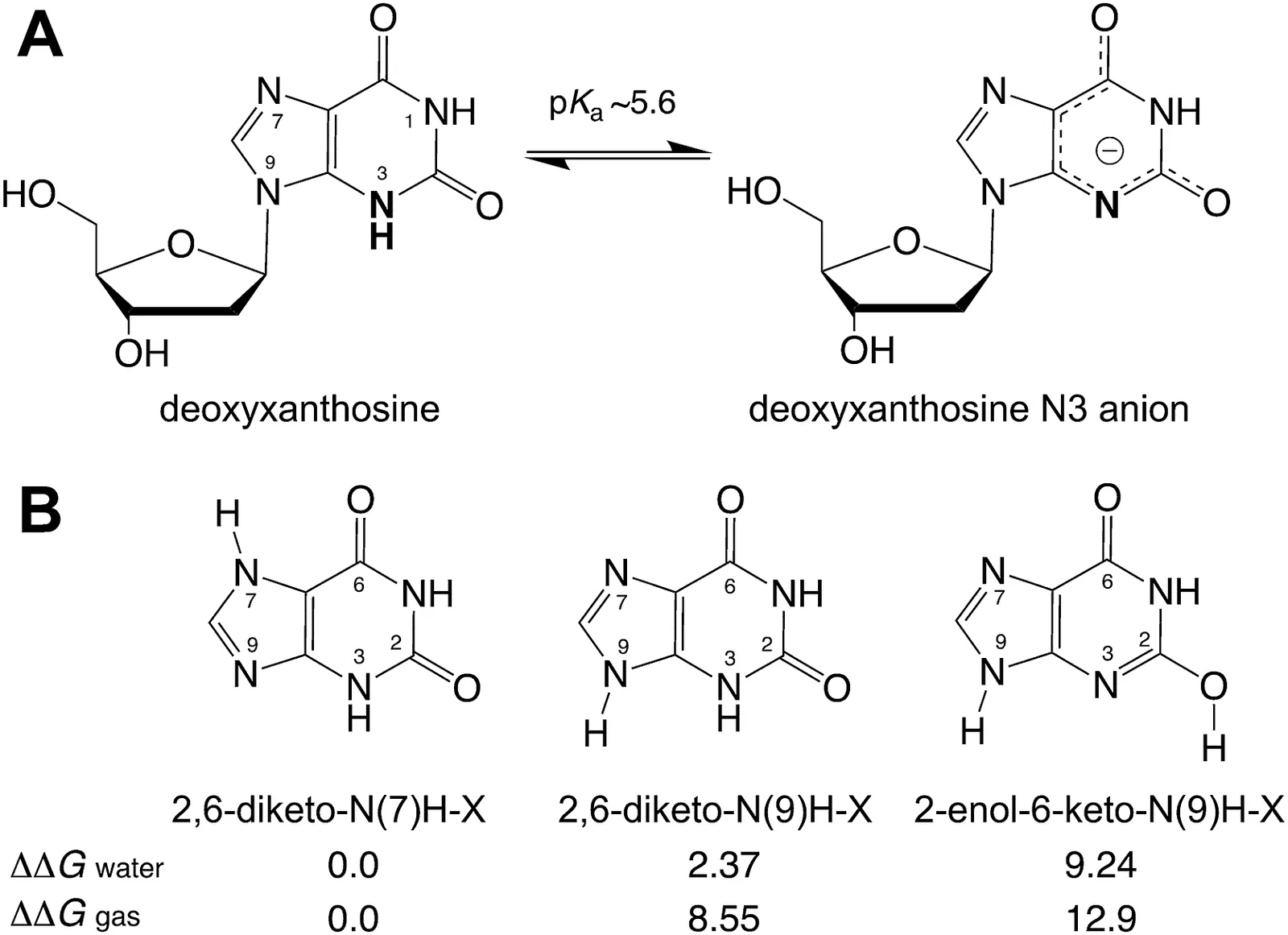
**Ionization of xanthosine and stability of xanthine tautomers.***A*, prior studies indicate that 2-deoxyxanthosine ionizes at N3 with a p*K*_a_ of ∼5.6 (see main text). *B*, calculated relative stability of the three most stable xanthine tautomers in water and the gas phase with values shown as the difference in free energy (ΔΔ*G*, kcal mol^–1^) with respect to the most stable species.

Previous studies indicate that the xanthine base of dX is largely anionic at physiological pH, due to relatively high N3-H acidity (Fig. 7*A*) (87). Indeed, xanthosine and xanthosine monophosphate ionize with p*K*_a_^N3^ of ∼5.6 (88, 89). Other studies showed that nonenzymatic *N*-glycosyl hydrolysis for dX in single strand DNA is pH-independent for pH > 7 and acid-catalyzed as pH falls from 7 to 2, with an apparent p*K*_a_ of ∼5 that likely reflects protonation at N3 to give neutral dX (79). We note that N7 of xanthine is unusually acidic for a purine base, with p*K*_a_^N7^ of 1.1 for the nucleoside (80). Hydrolysis of the *N*-glycosyl bond for the N3-anion of dX could involve expulsion of xanthine as an N9,N3-dianion, which is likely to have very poor LG ability (Fig. 5). Indeed, our theoretical calculations reveal that N9-H acidity of the xanthine N3-monoanion very low in water, Δ*G* = 22.6 kcal/mol, and the gas phase, Δ*G* = 416.5 kcal/mol; much lower than that for neutral xanthine or any of the neutral purines considered here (Table 2). This result indicates that glycosylase excision of xanthine from DNA is likely to be far more efficient for the neutral species *versus* the N3-anion.

Prior calculations for 2,6-diketo-xanthine show that stability is higher for the N7-H *versus* N9-H tautomer in the gas phase and water, indicating that the former predominates in both media (Fig. 7*B*) (69). This, together with ionization at N3 (p*K*_a_ ∼5.6), substantially complicates an experimental determination of the N9 p*K*_a_ for xanthine (87). As such, the calculated N9-H acidities presented here, together with a previously calculated N9 p*K*_a_ (Table 2) (80), inform the mechanistic understanding how xanthine is excised by MUG and other DNA glycosylases including human single-strand-selective monofunctional uracil DNA glycosylase, which removes xanthine from G·X and other X base pairs (A·X, C·X, T·X) (90). We also calculated the relative stability and N9-H acidity of xanthine N9-H tautomers, finding that the 2,6-diketo is the most stable species in water and the gas phase and has the highest N9 acidity in both media (Figs. 7*B* and S4). Together, these finding indicate that DNA glycosylases most likely excise the 2,6-diketo rather than other tautomers of xanthine from DNA.

### Does the purine specificity of TDG or MUG depend on N9 acidity?

**Figure 8:**
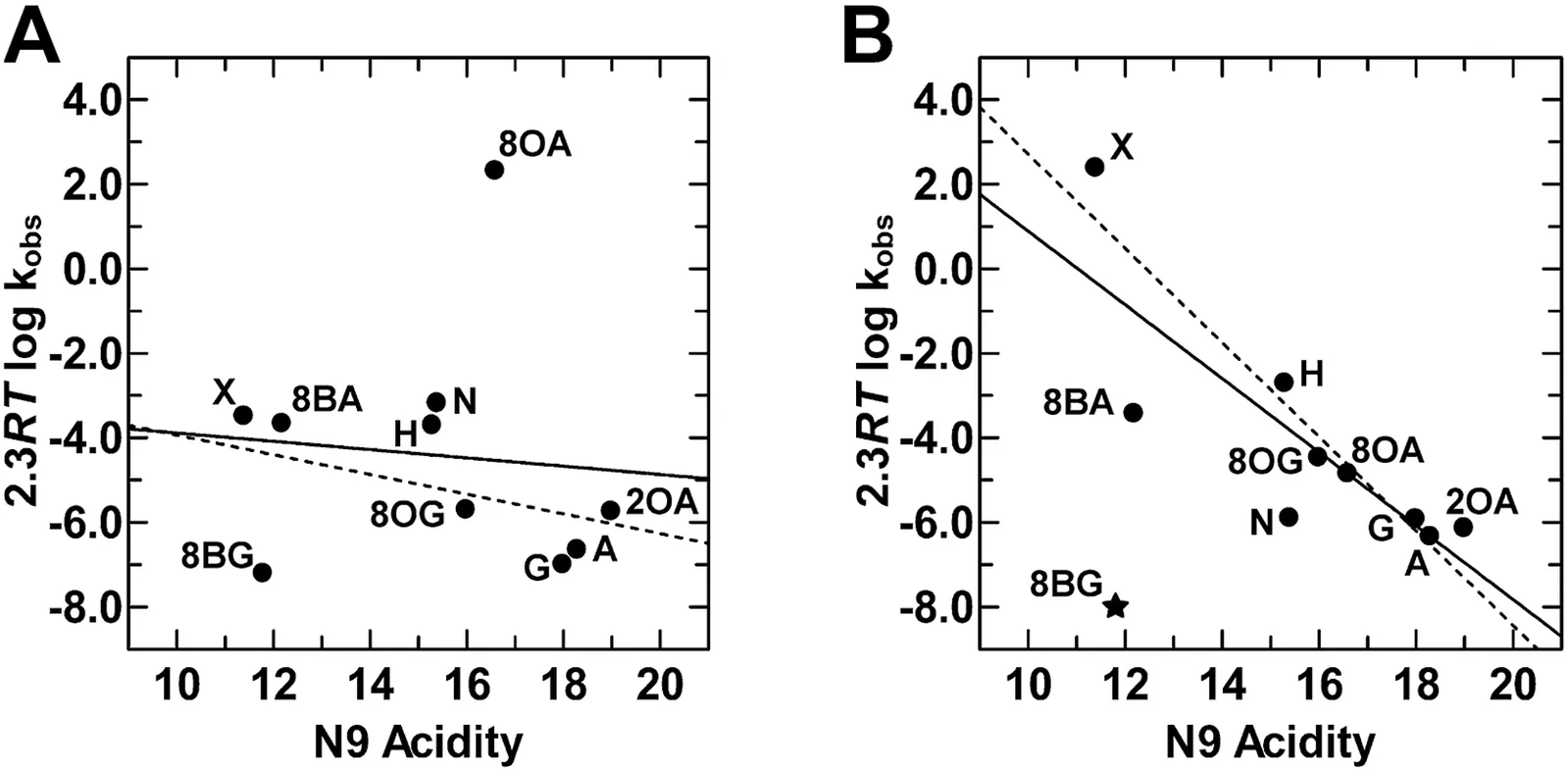
**Glycosylase activity (2.3*RT* log *k*_obs_) *versus* purine N9 acidity.** The dependence of glycosylase activity (2.3*RT* log *k*_obs_) on the calculated N9 acidity of purines in water is considered for TDG (*A*) and MUG (*B*) acting on 10 purine substrates. Linear fitting of the TDG data gives a correlation coefficient of *r*^2^ = 0.009 and slope of −0.10 ± 0.36 for all data points (*solid line*), and *r*^2^ = 0.170 and slope of *m* = −0.23 ± 0.19 when 8OA is excluded (*dashed line*). Linear fitting of the MUG data gives a correlation coefficient of *r*^2^ = 0.684 and a slope −0.87 ± 0.22 for all data except 8BG (*solid line*), and *r*^2^ = 0.829 and a slope of *m* = −1.12 ± 0.21 for all data except 8BA and 8BG (*dashed line*). Note that MUG activity for 8BG was not detected and the plotted value (*star*) reflects an upper limit (see main text). 8BA, 8-bromoadenine; 8BG, 8-bromoguanine; MUG, mismatch-specific uracil DNA-glycosylase; TDG, thymine DNA glycosylase.

We next consider the glycosylase activity of TDG and MUG in the context of purine N9 acidity. Our calculations show that 8OA exhibits the lowest N9 acidity in the gas phase, and among the lowest in water, indicating that it has relatively poor LG ability, which is remarkable given that TDG activity is at least four orders of magnitude higher for 8OA relative to the nine other purines examined here. While xanthine has the highest N9 acidity, TDG activity is over four orders of magnitude lower for X relative to 8OA. Although N9 acidity is much higher for 8BA *versus* 8OA, TDG activity is 24,000-fold lower for 8BA, even as the purines exhibit significant structural similarity (though 8BA is bulkier; Fig. 5). While 8OA and 8OG have similar N9-H acidity, TDG activity is nearly six orders of magnitude higher for 8OA *versus* 8OG. To provide a more rigorous analysis of the potential dependence of TDG activity on leaving group ability of the purine bases, we plotted (2.3*RT*)log *k*_obs_ *versus* N9-H acidity in water (Fig. 8*A*). We observe a poor correlation between these parameters when all data points are included in the fitting (*r*^2^ = 0.009) and an improved but still poor correlation when 8OA, the major outlier, is excluded (*r*^2^ = 0.170). Together, these results indicate that TDG specificity for 8OA over other purines is not significantly dependent on LG ability (N9 acidity).

The results for MUG indicate a more substantial dependence of glycosylase activity on purine N9 acidity (Fig. 8*B*). MUG exhibits the highest activity, by far, for excising X among the purines examined here, and X has the highest N9 acidity. Still, the results indicate that factors other than N9 acidity influence purine excision by MUG. For example, activity is very low for 8BA and not detected for 8BG, even as their N9 acidity is similar to that of X (in water). We observe a relatively good correlation between MUG activity and N9 acidity when 8BA and 8BG are excluded from the fitting, with *r*^2^ = 0.829 (Fig. 8*B*, dashed line), and the correlation is worse when 8BA is included (*r*^2^ = 0.684; solid line; 8BG remains excluded because the data point represents an upper limit for *k*_obs_). Together, the results indicate a substantial dependence of MUG activity on purine LG ability, which likely contributes to its high specificity for excising xanthine over other purines, as discussed below.

## Discussion

### Robust excision of xanthine by bacterial MUG

We find that MUG exhibits strikingly high activity for excising xanthine from G⋅X pairs in DNA, with *k*_obs_ = 55.0 ± 1.3 min^−1^, which is 6-fold greater than its robust activity for G⋅εC pairs and 57-fold higher than that for G⋅U mispairs, the substrate for which MUG was discovered. A prior study (Lee *et al.*) found that MUG excises X from G·X pairs with a rate constant of *k*_obs_ = 0.23 min^−1^, with similar activity for other X base pairs (C·X, A·X, T·X) (49). Thus, our results reveal that MUG has vastly (240-fold) higher activity for G·X pairs than had previously been thought. The prior study also reported no detectable MUG activity for excising hypoxanthine from G·H pairs, while we find readily detectable, albeit low activity for this purine lesion. We suspect that these discrepancies can be explained by an important difference in experimental conditions, namely enzyme concentration. Although both studies employed single turnover kinetics experiments, with [E] >> [S], the prior study used enzyme and substrate concentrations of 0.1 μM and 0.01 μM, respectively, while we used a 100-fold higher concentration of enzyme (10 μM) and a 50-fold higher concentration of substrate. Notably, another previous study (O’Neill *et al.*) reported that MUG exhibits relatively weak binding to a G·U substrate (*K*_0.5_ = 0.4 μM) such that a 5 to 10 μM concentration of MUG was required to obtain the saturating enzyme conditions needed to give maximal glycosylase activity (*k*_obs_ ≈ *k*_max_) in single turnover experiments (44). Our experiments were collected with a 10 μM concentration of MUG and our rate constants (*k*_obs_) agree with those of O’Neill *et al.* for substrates considered in both studies (G·H, G·T, G·U, G·εC). Together, these observations indicate that the results presented here reflect the maximal activity of MUG for excising xanthine from G·X mispairs.

### Specificity of TDG for 8OA

**Figure 9:**
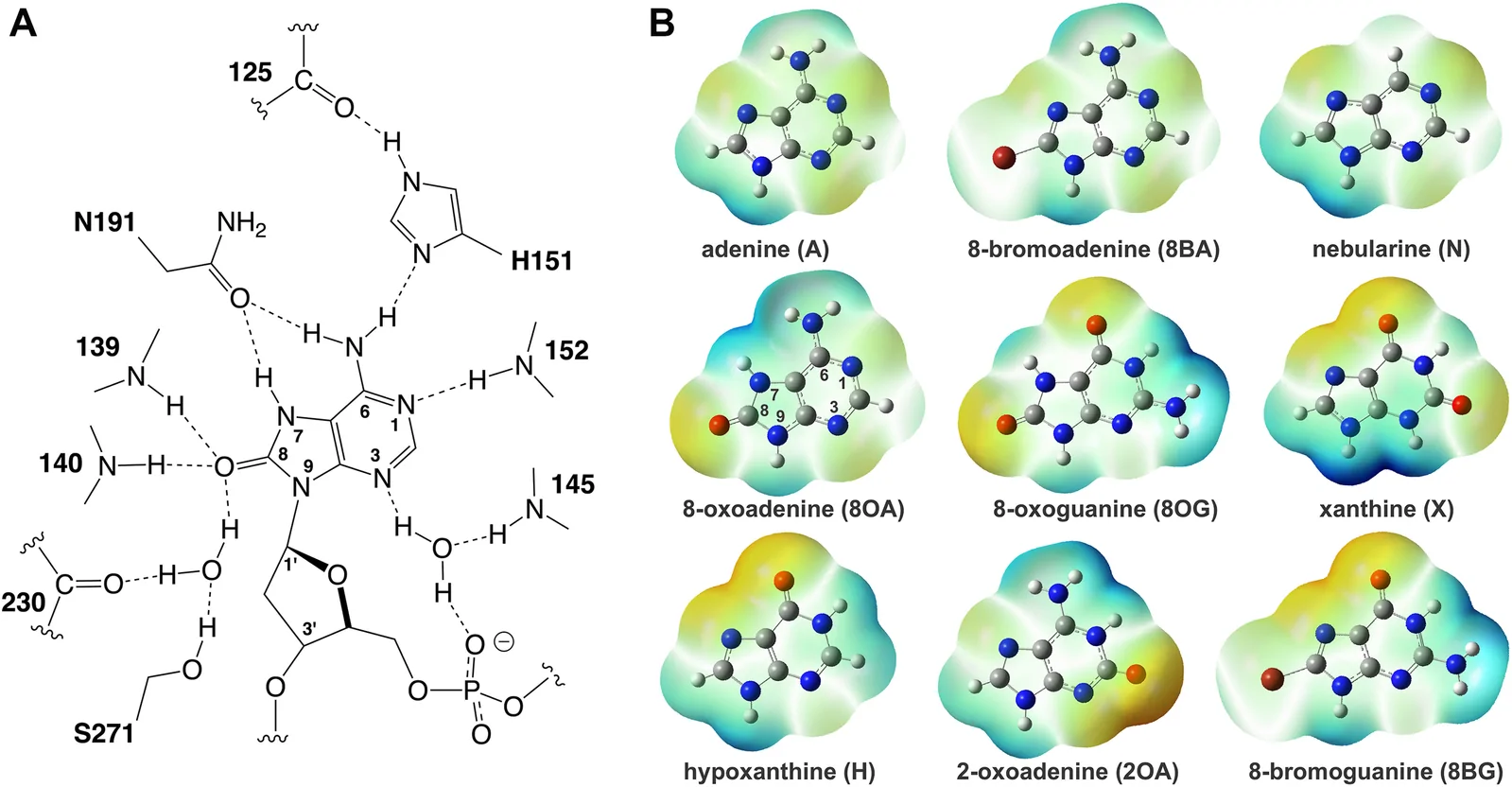
**Specificity of TDG for 8OA.***A*, model of TDG interactions with the flipped 8-oxo-dA nucleotide, as suggested by interactions seen in high resolution structures of TDG with dU (PDBID 5hf7), 5-formyl-dC (PDBID 5t2w), or 5-carboxyl-dC (PDBID 6u17) flipped into its active site (see main text). *B*, ESP maps of purines, rotated to the orientation of 8OA in *panel A*. 8OA, 7,8-dihydro-8-oxoadenine; TDG, thymine DNA glycosylase.

Our findings reveal that TDG activity for excising 8OA exceeds that for nine other purines by four to seven orders of magnitude, even as most of the purines have better LG ability than 8OA. Moreover, the rate enhancement of 10^9.2^ for excision of 8OA is 50,000-fold greater than that for H, the next best purine substrate, at least 500,000-fold above that for other purines (X, A, G) and exceeds that for uracil (10^8.2^) and thymine (10^7.1^). It should be noted that the differences in purine activity could be due in part to differences in the equilibrium for nucleotide flipping (*K*_flip_), the reversible conformational change in which a deoxynucleotide rotates from the DNA duplex into the active site, where *K*_flip_ could be higher for 8OA relative to other purines. Nevertheless, our results indicate that specificity also derives from the ability of TDG to enhance the LG ability of 8OA to a greater extent than other purines. Our prior studies show that TDG excision of 8OA is independent of pH for pH 6.0 to pH 9.0, indicating that LG activation does not involve protonation of the 8OA base (*i.e.*, general or specific acid catalysis) (21). As such, TDG acts on the neutral form of 8OA and stabilizes negative charge developing on the LG in the TS for bond cleavage. Although no structure of TDG bound to DNA with an 8OA substrate has been reported, high-resolution structures of TDG with other substrates (U, fC, caC) together with mutational studies for 8OA excision and the results here suggest a model for active site interactions that could promote 8OA excision (Fig. 9*A*) (20, 21, 65, 66, 91, 92). The backbone amide of residues 139 and 140, and an ordered water molecule, could interact with O8 of 8OA, similar to interactions between these moieties and the O2 oxygen of U, fC, and caC seen in prior TDG structures. The backbone amide of 152 could contact N1 of 8OA, as it does for the formyl oxygen of fC, a carboxyl oxygen of caC, and O4 of uracil. The N3 of 8OA could be contacted by an ordered water molecule that is coordinated by a DNA phosphate and the backbone amide of 145 and is seen in multiple TDG structures (with U, fC, and caC). Previous findings that the H151A mutation causes activity reductions of 3- to 35-fold for 8OA excision, depending on 8OA base pairing, suggest that His151 could contact the exocyclic amine (N6) of 8OA (21). Observation that the N191A mutation causes 8OA activity reductions of 40- to 400-fold indicates that Asn191 is essential for flipping and/or excision of 8OA, perhaps through contacts to N7-H and/or N6H_2_ of 8OA (21).

It is of interest to consider potential mechanisms underlying the specificity of TDG for 8OA *versus* the other purines examined here (Fig. 9*B*). The contribution of the O8 and N7-H moieties to TDG specificity for 8OA is illustrated by the 800,000-fold higher rate enhancement for excision 8OA *versus* A, and the 10,000-fold higher activity for 8OA *versus* N (which also lacks the exocyclic N6H_2_ of 8OA). The 24,000-fold higher activity for 8OA *versus* 8BA, which differ only at positions 7 and 8, could reflect the absence of O8 as a hydrogen bond acceptor and N7-H as a hydrogen bond donor for 8BA or the increased bulk of the 8-bromo *versus* 8-oxo substituent. The 750,000-fold higher activity for 8OA *versus* 8OG, which have similar N9 acidity and differ only at positions 1, 2, and 6, supports the importance of one or more features of 8OA for TDG specificity, including the exocyclic amine (rather than a carbonyl oxygen) at position 6, the presence of N1 as a hydrogen bond acceptor rather than donor (N1-H), or the absence of a substituent at C2. The importance of TDG interactions with positions 1, 2, or 6 of 8AO is also suggested by the 400-fold higher activity for 8BA *versus* 8BG, which have similar N9 acidity and exhibit the same chemical groups at positions 1, 2, and 6 as 8OA and 8OG, respectively.

### Specificity of MUG for xanthine

**Figure 10:**
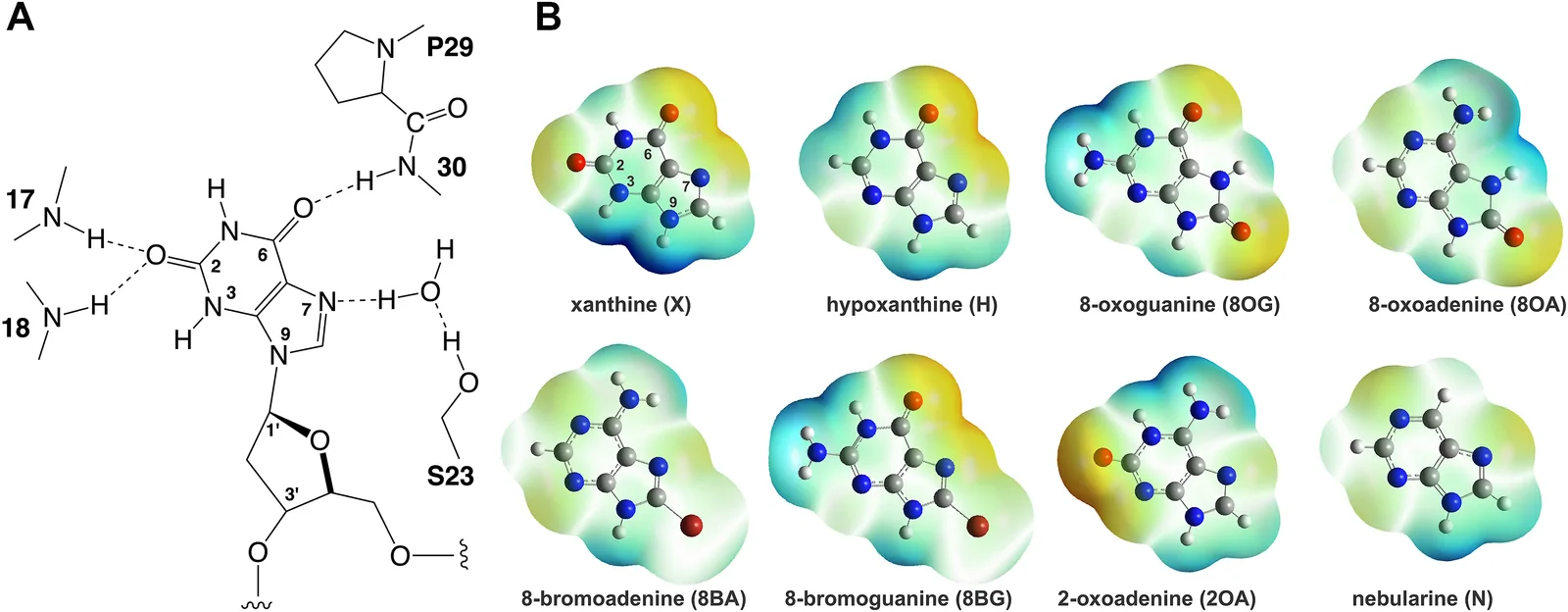
**Specificity of MUG for xanthine.***A*, model of MUG interactions with the flipped dX nucleotide, as suggested by interactions in a structure of DNA-bound MUG with dU flipped into its active site (PDBID 1mwj). *B*, ESP maps of purines, rotated to the orientation of 8OA in panel A. 8OA, 7,8-dihydro-8-oxoadenine; dX, 2′-deoxyxanthosine; MUG, mismatch-specific uracil DNA-glycosylase.

Our results show that MUG exhibits the highest activity for xanthine (X), the purine with the best inherent LG ability (highest N9 acidity), and its activity (log *k*_obs_) depends substantially on purine N9 acidity when data for 8 of 10 purines are fitted (*r*^2^ = 0.829; Fig. 8*B*). These results indicate that the favorable LG ability of X is likely an important factor in its rapid excision by MUG. Nevertheless, the extraordinarily high activity of MUG for X excision (*k*_obs_ = 55 min^−1^) and the higher rate enhancement for excision of X (10^7.8^) *versus* H (10^5.2^) and other purines (≤10^3.5^) suggests that MUG increases the LG ability of X to a greater extent than other purines. As noted for TDG, the differences in purine activity could be due in part to differences in nucleotide flipping (*K*_flip_), where *K*_flip_ could be higher for X relative to other purines (when the bases are paired with guanine). Although no structure of DNA-bound MUG with dX flipped into its active site has been reported, a structure with dU is available (46), suggesting a potential model for the interactions that mediate rapid excision of X (Fig. 10*A*). The interactions could potentially serve to stabilize the flipping of dX into the active site in addition to stabilizing negative charge on the LG during bond cleavage. The model predicts that the backbone amide of residues 17 and 18 contact O2 and the backbone amide of residue 30 contacts O6 of xanthine. Similar contacts are seen in the structure of MUG with dU flipped into its active site, where O2 and O4 of dU are contacted by the backbone amide of residues 17 and 30, respectively (46). Notably, residues 17, 18, and 30 of MUG correspond structurally to 139, 140, and 152 of TDG, for which backbone amides also contact O2 and O4 of dU (92). Our model also suggests that Ser23 of MUG contacts N7 of xanthine through a water molecule, although it could potentially make a direct contact with N7, as previously proposed (49). A prior study finds that the S23A mutation causes modest (3- to 9-fold) reductions in X excision activity, depending on X base pairing (49). Additional studies will be needed to test this model and define the mechanism for recognition and excision of X by MUG.

We next consider potential mechanisms that provide the specificity of MUG for X relative to the other purines examined here (Fig. 10*B*). The 5000-fold higher activity for X *versus* H, which differ at positions 2 and 3, could reflect the importance of MUG interactions with O2 and N3-H for excision of X, in addition to the much higher acidity of X (p*K*_a_ 7.3) *versus* H (p*K*_a_ 8.9). Observation that MUG activity is a million-fold higher for X *versus* N, and 220-fold higher for H *versus* N, points to the importance of the N1-H and O6 moieties for excision of X, as these groups are present on X and H but not N. Our finding that activity is 18,000- and 39 million-fold higher for X *versus* 8BA and 8BG, respectively, even as the brominated bases and X exhibit similar N9 acidity, suggests that the increased steric bulk of the bromo substituent is not tolerated by the MUG active site, which could impair nucleotide flipping. The very low activity observed for 2OA is consistent with its low N9 acidity, but it is excised ∼15-fold faster than predicted by the dependence of activity on N9 acidity (dashed line, Fig. 8*B*). This could reflect the similarity of 2OA with X at positions 1, 2, 7, and 8 that could enable interactions with MUG to enhance the LG ability of 2OA. Taken together, these observations indicate that while LG ability is likely a key factor in the specificity of MUG for xanthine over the other purines considered here, specificity also depends on steric and electrostatic interactions that enable MUG to flip dX into its active site and enhance the LG ability of the xanthine base.

### Divergent specificity of TDG and MUG

**Figure 11:**
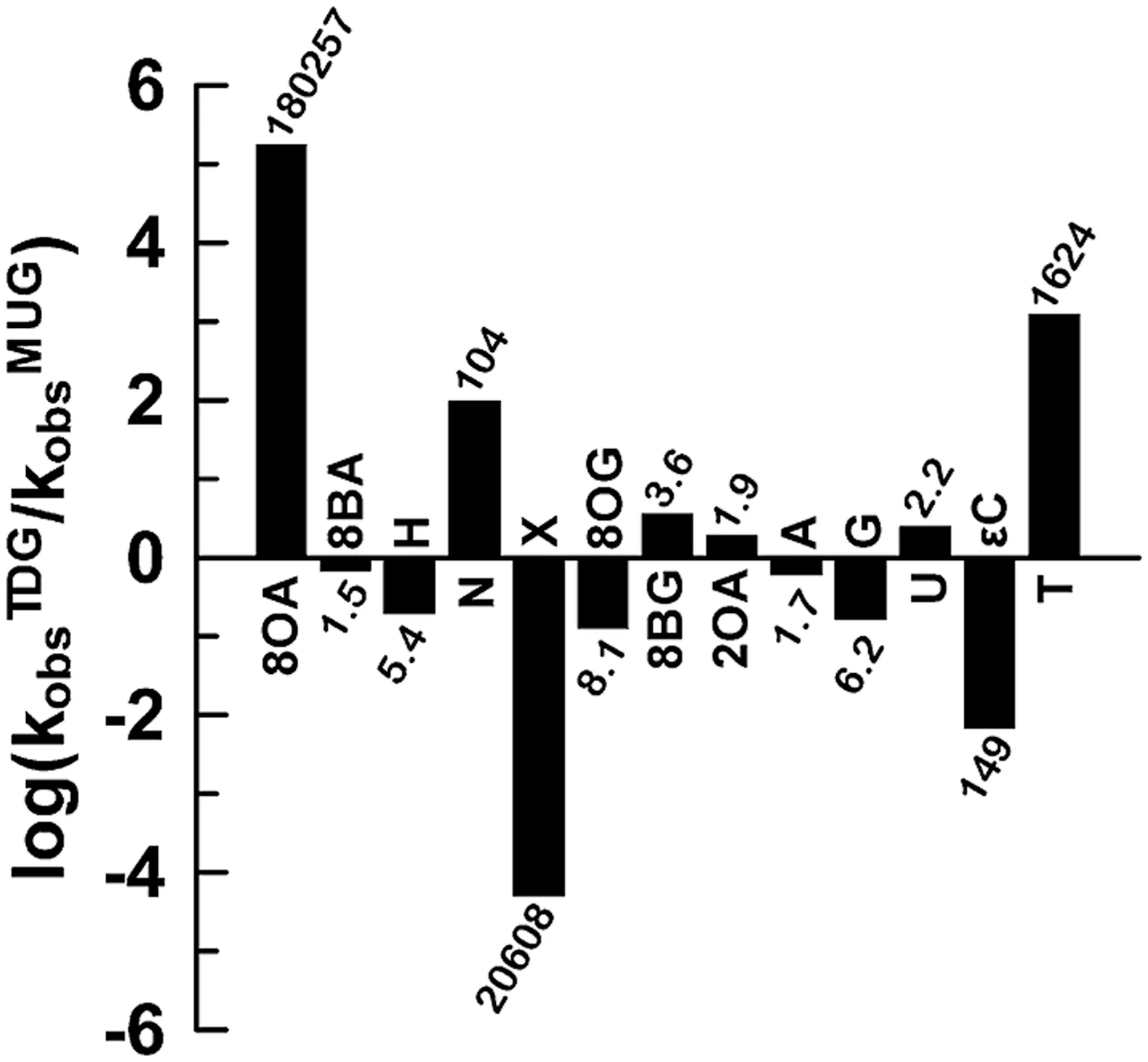
**Activity of TDG and MUG varies dramatically for some purine and pyrimidine lesions.** The ratio of activity for TDG and MUG for purine and pyrimidine substrates (note log format). The labels include only the base that is excised, though this base was paired with guanine for all substrates. The activity ratio for G⋅8BG represents a lower limit because MUG exhibited no detectable activity for this substrate. 8BG, 8-bromoguanine; MUG, mismatch-specific uracil DNA-glycosylase; TDG, thymine DNA glycosylase.

Our results further define the similarities and vast differences in activity for human TDG and *E coli* MUG (Fig. 11). These homologs have similar activity for G·U mispairs, the first common substrate identified for members of the TDG-MUG family (42). Considering the purines examined here, they exhibit similar activity (*k*_obs_ within 2-fold) for G·8BA, G·2OA, and G·A substrates and modest activity differences (up to 8-fold) for G·H, G·8OG, and G·G substrates. By contrast, the activity of TDG exceeds that of MUG by over 100-fold for G·N pairs and by over 1600-fold for G·T pairs, while the activity for a G·εC substrate is 150-fold higher for MUG *versus* TDG. The most striking differences are for G·X and G·8OA, where MUG activity is greater by four orders of magnitude for G·X, and TDG activity predominates by five orders of magnitude for G·8OA.

While TDG and MUG share 32% sequence identity and exhibit similar overall folds, there are major differences in many active site residues. For example, His151, Tyr152, and Asn191 of TDG are replaced by Pro29, Phe30, and Lys68, respectively, in the corresponding positions of *E coli* MUG, as shown by a structure-based alignment (45) (Figs. 9*A* and 10*A*). As noted previously, Asn191 and His151 are important for 8OA excision by TDG, and the Y152F mutation reduces the activity for 8OA by 3- to 16-fold, depending on 8OA base pairing (21). The variance in these residues, and possibly others, can likely explain the vast differences in purine specificity observed here for TDG and MUG.

**Figure 12:**
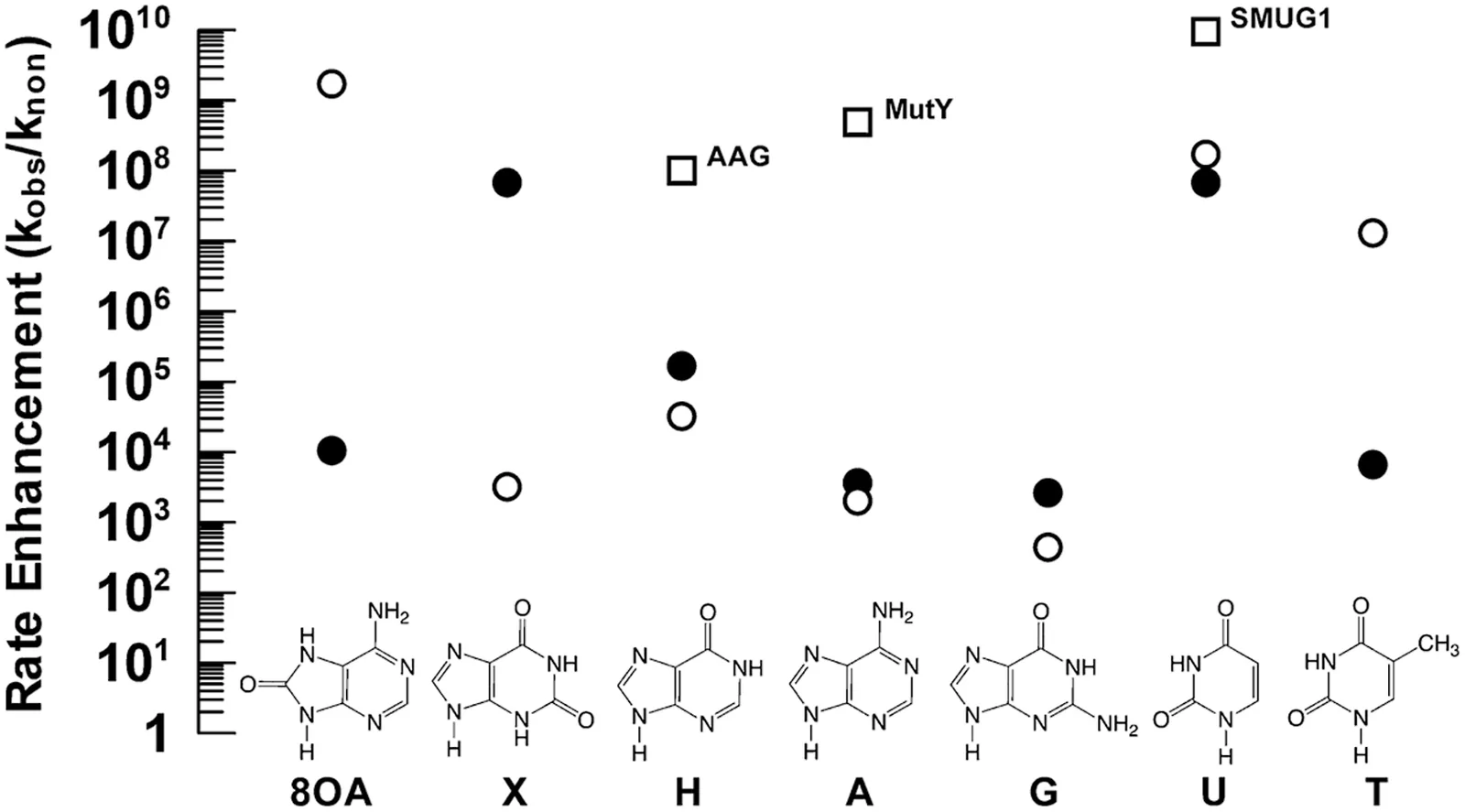
**Rate enhancements exhibited by TDG, MUG, and other DNA glycosylases.** Rate enhancements of TDG (*open circles*) and MUG (*filled circles*) for five purines and two pyrimidines using data from Table 1. Also shown are rate enhancements for three other DNA glycosylases (*squares*) including AAG excision of H, MutY excision of A, and SMUG1 excision of U (see main text). MUG, mismatch-specific uracil DNA-glycosylase; SMUG1, single-strand-selective monofunctional uracil DNA glycosylase; TDG, thymine DNA glycosylase.

Our results indicate that purine excision by TDG is largely independent of LG ability (N9 acidity) while that of MUG shows some dependence on LG ability (Fig. 8). Notably, this difference is also reflected in the range of rate enhancements exhibited by the two enzymes (Fig. 12). A glycosylase that shows a very strong dependence of activity on *N*-glycosyl bond stability, which typically depends largely on inherent LG ability of the nucleobase, can be expected to exhibit a relatively narrow range of rate enhancements. For example, *E*. *coli* AlkA exhibits rate enhancements that vary by no more than threefold (10^5.0^ to 10^5.5^) for excision of six different purines (68). In sharp contrast, the rate enhancements observed here for purine excision by TDG cover a range of over four million-fold, with a high of 10^9.2^ for 8OA and a low of 10^2.6^ for G. The rate enhancements for MUG cover a narrower range of 27,500-fold, with a high of 10^7.8^ for X and a low of 10^3.4^ for G. These observations are consistent with the finding that the specificity of TDG for excision of 8OA over other purines is largely independent of N9 acidity while the specificity of MUG for xanthine exhibits a greater dependence on purine N9 acidity.

### Biological implications

Together, the results presented here and in previous studies suggest that 8OA could be an important biological substrate for TDG. As noted previously, our results reveal that TDG activity is four to seven orders of magnitude greater for 8OA than other purines and its rate enhancement of 10^9.2^ for excising 8OA is at least 50,000-fold higher than that for other purines (H, X, A, G) and higher than that for uracil (10^8.2^) and thymine (10^7.1^) (Fig. 12). Notably, the catalytic power of TDG for 8OA is comparable to or higher than that observed for other DNA glycosylases acting on biological substrates including AlkA for excision of 7-methyl-G (10^5.5^) (68), AAG for hypoxanthine (10^8.0^) (93), MutY for adenine (10^8.4^) (78), and single-strand-selective monofunctional uracil DNA glycosylase for uracil (10^9.7^) (59), though lower than that for uracil excision by UNG (10^11.7^) (78, 94), which has unusually high catalytic power for a DNA glycosylase. Three highly conserved TDG residues (H151, Y152, and N191) are required for efficient excision of 8OA and they are generally much less important for the excision of pyrimidine substrates including T, U, fC, and caC (20, 21). Prior findings show that TDG is required for the excision of 8OA in murine whole cell-free extracts (19), and no other human glycosylases are known to exhibit substantial 8OA excision activity *in vitro* (30, 95, 96). While these observations suggest that 8OA may be an important biological substrate for TDG, additional studies are warranted to define the activity of TDG for excising 8OA in human cells.

Our findings reveal that the activity of MUG for excision of xanthine exceeds that for excision of nine other purines by 10^3.7^- to 10^7.6^-fold and exceeds the uracil excision activity by nearly 60-fold. Moreover, MUG exhibits a rate enhancement of 10^7.8^ for excising X, which is at least 400-fold higher than that for other purines (8OA, H, A, and G) and equivalent to that for uracil (Fig. 12). Notably, the rate enhancement of MUG for X excision is in the range of that observed for other DNA glycosylases acting on biological substrates, including AlkA (10^5.5^, 7 mG), AAG (10^8.0^, H), and MutY (10^8.4^, A), as noted previously (68, 78, 93). These observations, together with prior findings that MUG is required for excision of xanthine from DNA in *E*. *coli* whole cell extracts (49), support the idea that xanthine may be an important biological substrate of MUG enzymes. As such, MUG-initiated BER could protect against the mutagenic effects of X·C pairs that arise *via* spontaneous or nitrosative guanine deamination (50, 51, 52, 53).

## Experimental procedures

### Oligodeoxynucleotides (ODNs) and duplex DNA

Oligodeoxynucleotides (ODNs) were obtained from IDT, or, for those containing a modified base (*e.g.*, 8OA) from the Keck Foundation Biotechnology Resource Laboratory of Yale University using phosphoramidites obtained from Glen Research. ODNs were purified by reverse phase HPLC using an XBridge Oligonucleotide BEH C18 OBD Prep column (Waters Corp.) as described (97). ODN purity was assessed by HPLC using a DNAPac PA200 RS anion-exchange column (Thermo Fisher Scientific) under denaturing (pH 12) conditions (14). ODNs were exchanged into 0.02 M Tris–HCl pH 8, 0.04 M NaCl, 1 mM EDTA and the concentration was determined by absorbance (260 nm) using extinction coefficients calculated with the nearest-neighbor method (15). The 28 bp duplex DNA substrates contained a target strand, 5′-ACC AGT CCA TCG CTC AxG TAC AGA GCT G, where x = T, U, εC, or one of 10 purines (Fig. 1), and a complementary strand that pairs the target base (x) with G. The 28 bp duplexes were prepared by mixing the target and complementary strands, heating to 85 °C, and slowly cooling to RT. Purified ODNs and duplex DNA substrates were stored at −20 °C.

### Enzyme purification

Human TDG was expressed in *E. coli* BL21(DE3) and purified at 4 °C using metal (Ni^2+^) nickel affinity, SP Sepharose, and Capto HiRes Q columns (Cytiva), as described (15, 98). *E coli* MUG was expressed in *E. coli* BL21(DE3) cells at 37 °C and purified using metal affinity, ion exchange where the protein was loaded through tandem Q-SP sepharose and eluted from the SP column, followed by size exclusion (Superdex 200) chromatography (Cytiva), as described (20). The concentration of purified enzyme was determined by absorbance at 280 nm using an extinction coefficient of ε^280^ = 31.5 mM^−1^ cm^−1^ for TDG and ε^280^ = 25.6 mM^−1^ cm^−1^ for MUG (99, 100). Purity was confirmed by SDS-PAGE and purified enzyme was flash-frozen in small aliquots and stored at −80 °C.

### Glycosylase activity assays

Single turnover kinetics reactions were performed at 23 °C in HEN.1 buffer (0.02 M Hepes pH 7.5, 0.1 M NaCl, 0.2 mM EDTA) using a DNA substrate concentration of 0.5 μM and an enzyme concentration of 2.5 μM or 5.0 μM for TDG and 10 μM for MUG. We find that a 10 μM concentration of MUG is saturating for the a range of substrates in single turnover kinetics experiments, consistent with a prior study that employed similar conditions (44). The reactions were initiated by adding concentrated enzyme to samples that contained all other components and were rapidly halted at specific time points by adding quench solution (0.1 M NaOH, 0.01 M EDTA, final). Quenched samples were heated at 85 °C (3 min) to quantitatively cleave the DNA backbone at the glycosylase-generated abasic site. The resulting DNA fragments were resolved by HPLC with a DNAPac PA200 RS anion-exchange column (4.6 mm × 50 mm; Thermo Fisher Scientific) under denaturing (pH 12) conditions using mobile phase comprised of eluent A (0.02 M sodium phosphate, 0.03 M sodium perchlorate, pH 12) and eluent B (0.02 M sodium phosphate, 0.50 M sodium perchlorate, pH 12), as described (14, 21). The elution of DNA fragments was monitored by absorbance (260 nm), and integrals for peaks corresponding to the intact and cleaved target strand were used to determine the fraction product for a given reaction sample. Progress curves (fraction product *versus* time) were fitted to a single exponential equation (Equation 1) by nonlinear regression using Grafit 5 (101).

(1) $$\text{Fraction}\;\text{product}=A\left(1\;–\;\exp\left(–k_{obs}t\right)\right)$$

Where *A* is the amplitude, *k*_obs_ is the rate constant, and *t* is reaction time. For reactions that exhibited very low TDG activity and did not proceed to completion (∼100% product) in a reasonable timeframe, the data (fraction product *versus* time) were fitted to a linear equation, where *k*_obs_ is given by the slope.

### Computational studies

The gas-phase calculations were conducted at B3LYP-D3(BJ)/6-311++G(2d,p) using Gaussian16 (102, 103, 104, 105, 106, 107, 108). All geometries were fully optimized, and frequencies were calculated (no imaginary frequencies were found). All reported values are in Gibbs free energy at 298 K in kcal/mol. The solvation model density method of Truhlar and coworkers was used for all the calculations in water (109, 110).

## Data availability

The data supporting the findings of this study are available within the article and the supporting information.

## Supporting information

This article contains supporting information.

## Conflict of interest

The authors declare that they have no conflicts of interest with the contents of this article.
