# Supplementary material for: Robust excision of different purine lesions by homologs thymine DNA glycosylase and mismatch-specific uracil DNA-glycosylase
Source: J Biol Chem. 2026 Aug 13;302(10):113446. doi: 10.1016/j.jbc.2026.113446 (PMC13579944; doi:10.1016/j.jbc.2026.113446)
Supplement: Supplementary file 1 [file mmc1.pdf]

Supplementary Information

Robust excision of different purine lesions by homologs thymine DNA glycosylase and mismatch-specific uracil DNA-glycosylase

Kurt B. Espinosa[1], Xiao Ding,[2] Hardler W. Servius[1], Lakshmi S. Pidugu,[1] Jeehiun K. Lee,[2,] and Alexander C. Drohat[1,3,*]*

[1]Department of Biochemistry and Molecular Biology, University of Maryland School of Medicine, Baltimore, MD 21201, USA, [2]Department of Chemistry and Chemical Biology, Rutgers, The State University of New Jersey, New Brunswick, NJ 08854, USA, [3]Cancer Biology Program, University of Maryland Marlene and Stewart Greenebaum Comprehensive Cancer Center, Baltimore, MD 21201, USA

* For correspondence, Alex Drohat, adrohat@som.umaryland.edu, Jeehiun Lee, jeehiun@rutgers.edu

Table of Contents

Figure S1. Construct and nomenclature for the 28 bp DNA substrates.............................. 6
Figure S2. Correlation between calculated N9 acidity and N9 pK_a for purines..................... 6
Figure S3. Relative stability of N9-H tautomers for 8-oxoadenine...................................... 7
Figure S4. Relative stabilities and N9 acidities of xanthine N9-H tautomers........................ 7
Cartesian coordinates and energies (in Hartree) for species calculated........................ 8
Calculated Structures (Adenine, A) 8
Calculated Structures (8-bromoadenine, 8BA)... 9
Calculated Structures (Hypoxanthine, H)... 9
Calculated Structures (2-oxoadenine, 2OA)... 10
Calculated Structures (Guanine, G)... 10
Calculated Structures (8-oxoguanine, 8OG).. 10
Calculated Structures (8-bromoguanine, 8BG) 11
Calculated Structures (Xanthine, X)... 11
Calculated Structures (Nebularine, N)... 12
Calculated Structures (Thymine, T).. 13
Calculated Structures (Uracil, U)... 13
Calculated Structures (Ethenocytosine, εC) 13
Result for 2OA.log 14
Result for 2OA-N9H.log 14
Result for 2OA-N9H_water.log 15
Result for 2OA_water.log 16
Result for 8BA.log... 17
Result for 8BA-N9H.log.. 17
Result for 8BA-N9H_water.log... 18
Result for 8BA_water.log.. 19
Result for 8BG.log 19
Result for 8BG-N9H.log 20
Result for 8BG-N9H_water.log 21
Result for 8BG_water.log 22
Result for 8OA_6amino8enol(a).log.. 22
Result for 8OA_6amino8enol(a)-N9H.log... 23
Result for 8OA_6amino8enol(a)-N9H_water.log...................................... 24
Result for 8OA_6amino8enol(a)_water.log... 25

Result for 8OA_6amino8enol(b).log 25
Result for 8OA_6amino8enol(b)-N9H.log 26
Result for 8OA_6amino8enol(b)-N9H_water.log 27
Result for 8OA_6amino8enol(b)_water.log 28
Result for 8OA_6amino8keto.log 28
Result for 8OA_6amino8keto-N9H.log 29
Result for 8OA_6amino8keto-N9H_water.log 30
Result for 8OA_6amino8keto_water.log 31
Result for 8OA_6imino8enol(a).log 31
Result for 8OA_6imino8enol(b).log 32
Result for 8OA_6imino8enol(c).log 33
Result for 8OA_6imino8enol(d).log 34
Result for 8OA_6imino8enol(e).log 34
Result for 8OA_6imino8keto(a).log 35
Result for 8OA_6imino8keto(a)-N9H.log 36
Result for 8OA_6imino8keto(a)-N9H_water.log 37
Result for 8OA_6imino8keto(a)_water.log 37
Result for 8OA_6imino8keto(b).log 38
Result for 8OA_6imino8keto(b)-N9H.log 39
Result for 8OA_6imino8keto(b)-N9H_water.log 40
Result for 8OA_6imino8keto(b)_water.log 40
Result for 8OA_6imino8keto(c).log 41
Result for 8OA_6imino8keto(d).log 42
Result for 8OG.log 43
Result for 8OG-N9H.log 43
Result for 8OG-N9H_water.log 44
Result for 8OG_water.log 45
Result for A.log 46
Result for A-N9H.log 46
Result for A-N9H_water.log 47
Result for A_water.log 48
Result for eC.log 49
Result for eC-N1H.log 49
Result for eC-N1H_water.log 50
Result for eC_water.log 51
Result for G.log 52
Result for G-N9H.log 52
Result for G-N9H_water.log 53
Result for G_water.log 54
Result for H.log 55
Result for H-N9H.log 55
Result for H-N9H_water.log 56
Result for H_water.log 57

Result for N.log 57
Result for N-N9H.log 58
Result for N-N9H_water.log 59
Result for N_water.log 59
Result for T.log 60
Result for T-N1H.log 61
Result for T-N1H_water.log 62
Result for T_water.log 62
Result for U.log 63
Result for U-N1H.log 64
Result for U-N1H_water.log 64
Result for U_water.log 65
Result for X_26diketoN7H.log 66
Result for X_26diketoN7H_water.log 67
Result for X_2enol6enol(a).log 67
Result for X_2enol6enol(a)-N9H.log 68
Result for X_2enol6enol(a)-N9H_water.log 69
Result for X_2enol6enol(a)_water.log 69
Result for X_2enol6enol(b).log 70
Result for X_2enol6enol(b)-N9H.log 71
Result for X_2enol6enol(b)-N9H_water.log 72
Result for X_2enol6enol(b)_water.log 72
Result for X_2enol6enol(c).log 73
Result for X_2enol6enol(c)-N9H.log 74
Result for X_2enol6enol(c)-N9H_water.log 75
Result for X_2enol6enol(c)_water.log 75
Result for X_2enol6enol(d).log 76
Result for X_2enol6enol(d)-N9H.log 77
Result for X_2enol6enol(d)-N9H_water.log 77
Result for X_2enol6enol(d)_water.log 78
Result for X_2enol6keto(a).log 79
Result for X_2enol6keto(a)-N9H.log 80
Result for X_2enol6keto(a)-N9H_water.log 80
Result for X_2enol6keto(a)_water.log 81
Result for X_2enol6keto(b).log 82
Result for X_2enol6keto(b)-N9H.log 83
Result for X_2enol6keto(b)-N9H_water.log 83
Result for X_2enol6keto(b)_water.log 84
Result for X_2keto6keto.log 85
Result for X_2keto6keto-N9H.log 85
Result for X_2keto6keto-N9H_water.log 86
Result for X_2keto6keto_water.log 87
Result for X_N3anion.log 88

Result for X_N3anion-N9H.log 88
Result for X_N3anion-N9H_water.log 89
Result for X_N3anion_water.log 90
Result for 2OA+N7H.log........ 90
Result for 2OA+N7H_water.log 91
Result for 8BA+N7H.log 92
Result for 8BA+N7H_water.log 93
Result for 8BG+N7H.log........ 94
Result for 8BG+N7H_water.log 94
Result for 8OA+N7H.log........ 95
Result for 8OA+N7H_water.log 96
Result for 8OG+N7H.log 97
Result for 8OG+N7H_water.log 97
Result for A+N7H.log 98
Result for A+N7H_water.log 99
Result for G+N7H.log........ 100
Result for G+N7H_water.log 100
Result for H+N7H.log........ 101
Result for H+N7H_water.log 102
Result for N+N7H.log........ 103
Result for N+N7H_water.log 103
Result for X+N7H.log 104
Result for X+N7H_water.log 105

```
                  +1
5´-ACCAGTCCATCGCTCAxGTACAGAGCTG-3´
3´-TGGTCAGGTAGCGAGTGCATGTCTCGAC-5´
```

Figure S1. **Construct and nomenclature for the 28 bp DNA substrates**. The substrate nomenclature used herein is "G·x", where x represents the target base. The "+1" indicates the base (G) positioned immediately 3′ of the target base.

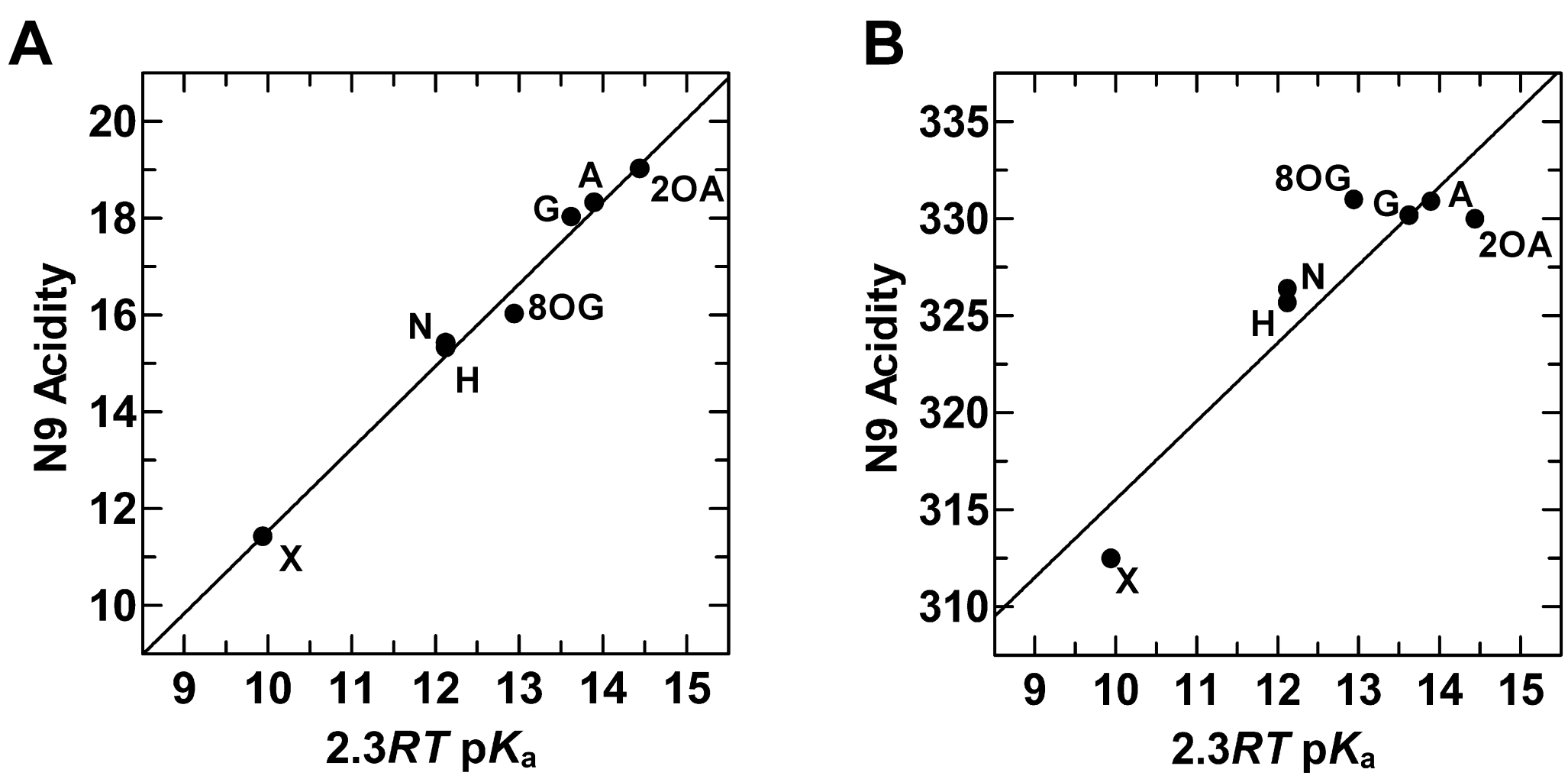

Figure S2. **Correlation between calculated N9 acidity and N9 pK_a for purines**. (A) Linear fitting of the calculated N9 acidity in water (ΔG, kcal/mol) versus 2.3RT pK_a^{N9} gives a slope of m = 1.7 ± 0.1 and a correlation coefficient of r^2 = 0.988. (B) Linear fitting of the calculated N9 acidity in the gas phase (ΔG, kcal/mol) versus 2.3RT pK_a^{N9} gives a slope of m = 4.0 ± 0.8 and a correlation coefficient of r^2 = 0.845. Values used for fitting are provided in Table 2 of the main text. pK_a values for 8OA, 8BA, and 8BG are not included in the fitting as they have not been reported (to our knowledge).

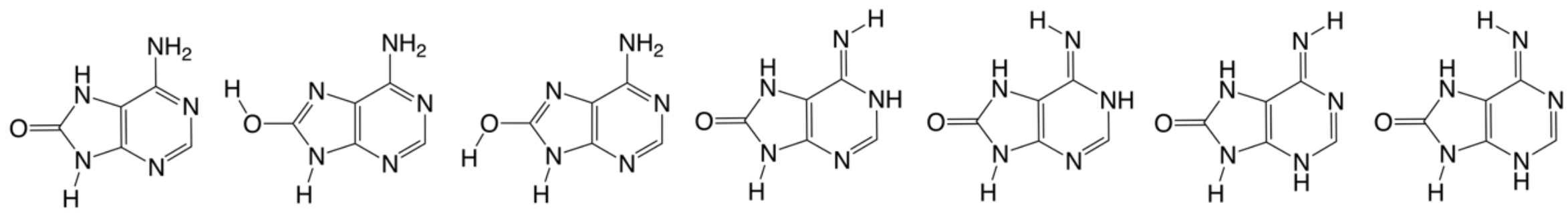

	6-amino-8-keto	6-amino-8-enol (a)	6-amino-8-enol (b)	6-imino-8-keto (a)	6-imino-8-keto (b)	6-imino-8-keto (c)	6-imino-8-keto (d)
ΔΔ***G* gas**	0.0	10.2	15.4	13.4	12.3	22.4	27.7

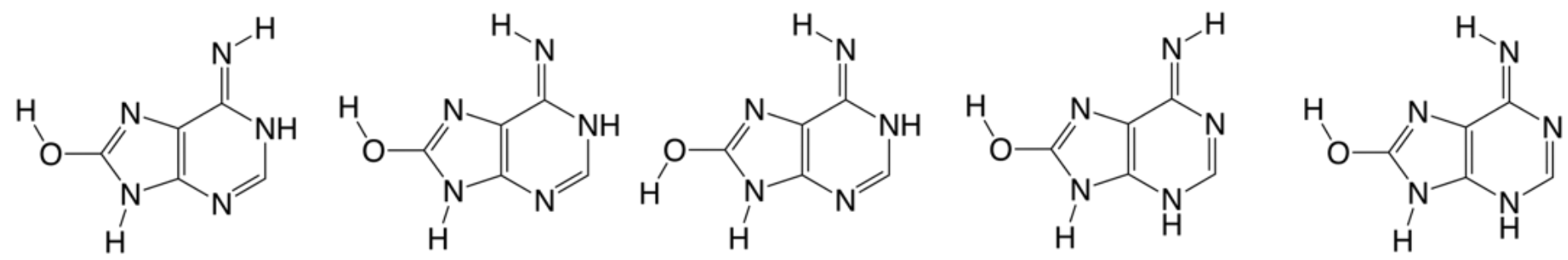

	6-imino-8-enol (a)	6-imino-8-enol (b)	6-imino-8-enol (c)	6-imino-8-enol (d)	6-imino-8-enol (e)
ΔΔ***G* gas**	28.6	23.1	28.6	40.4	41.0

Figure S3. Relative stability of N9-H tautomers for 8-oxoadenine. Calculated relative stabilities of 8-enol and 6-imino tautomers of 8-oxoadenine in the gas phase, with values shown as the difference in free energy (ΔΔ*G*, kcal mol^{-1}) with respect to the most stable species (ΔΔ*G* = 0).

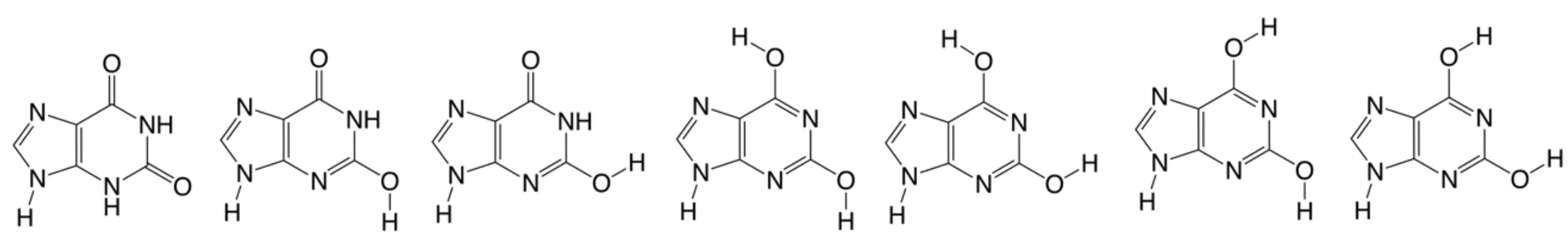

	2-keto-6-keto	2-enol-6-keto (a)	2-enol-6-keto (b)	2-enol-6-enol (a)	2-enol-6-enol (b)	2-enol-6-enol (c)	2-enol-6-enol (d)
ΔΔ*G* water	0.0	6.9	8.8	13.5	13.6	13.2	13.2
acidity, water	11.4	15.9	15.2	15.9	16.1	16.1	16.3
ΔΔ*G* gas	0.0	4.4	10.3	9.2	8.4	8.1	8.3
acidity, gas	312.4	326.2	327.4	323.8	325.2	326.3	327.6

Figure S4. Relative stabilities and N9 acidities of xanthine N9-H tautomers. Calculated relative stabilities of the six most stable N9-H tautomers of xanthine in the gas phase and water, with values shown as the difference in free energy (ΔΔ*G*, kcal mol^{-1}) with respect to the most stable species (ΔΔ*G* = 0). Also shown are the calculated N9 acidities for each tautomer, in water and the gas phase.

Cartesian coordinates and energies (in Hartree) for species calculated.

Calculated Structures (Adenine, A)

Figure S5. Structure of A, Deprotonated N9-anion of A, and Protonated N7-cation of A in the Gas Phase and Water Phase

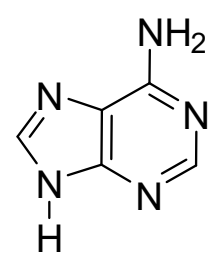
A
-467.410588

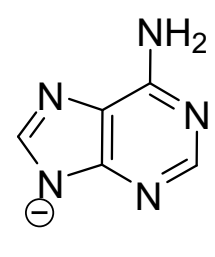
A-N9H
-466.873456

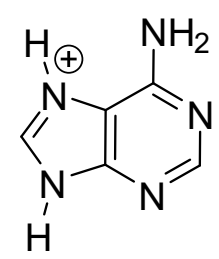
A+N7H
-467.758024

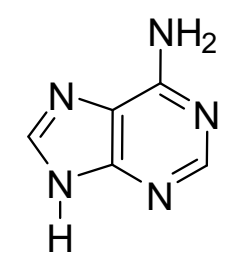
A_water
-467.437599

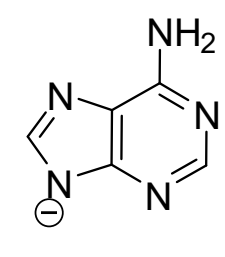
A-N9H_water
-466.975194

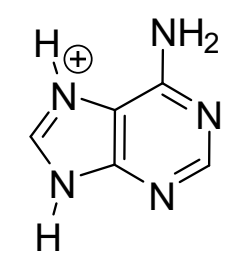
A+N7H_water
-467.758024

Calculated Structures (8-oxoadenine, 8OA)

Figure S6. Structure of Most Stable N9H Neutral 8OA Tautomers in the gas phase.

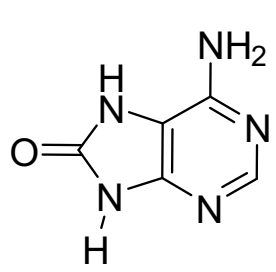
8OA_6amino8keto
-542.683274

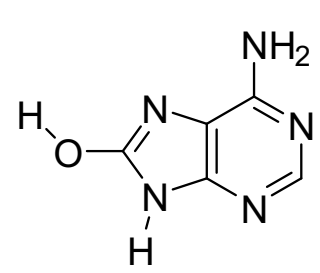
8OA_6amino8enol(a)
-542.667051

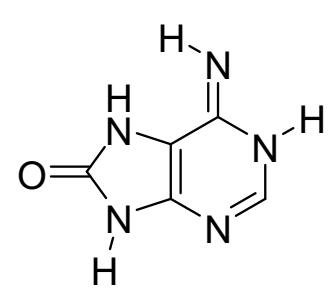
8OA_6imino8keto(b)
-542.663683

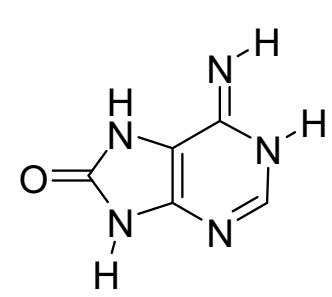
8OA_6imino8keto(a)
-542.661859

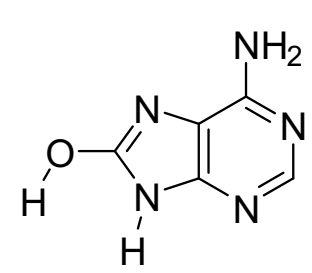
8OA_6amino8enol(b)
-542.658768

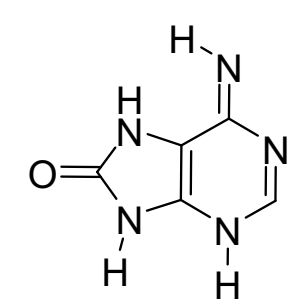
8OA_6imino8keto(c)
-542.639198

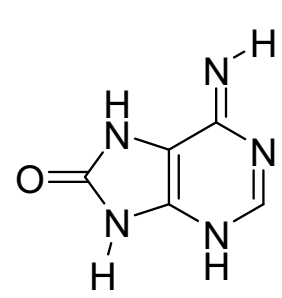
8OA_6imino8keto(d)
-542.647570

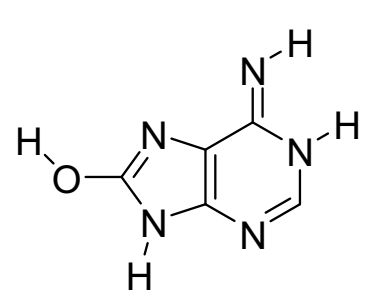
8OA_6imino8enol(a)
-542.637614

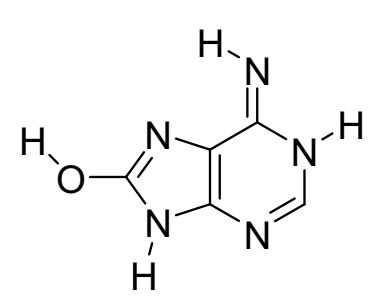
8OA_6imino8enol(b)
-542.646521

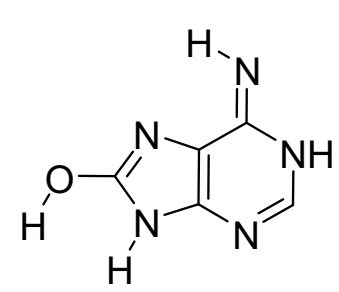
8OA_6imino8enol(c)
-542.637738

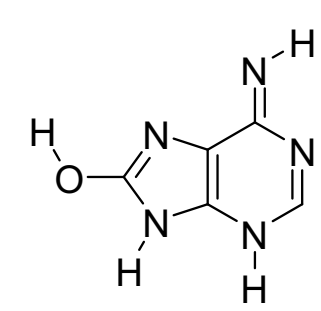
8OA_6imino8enol(d)
-542.618892

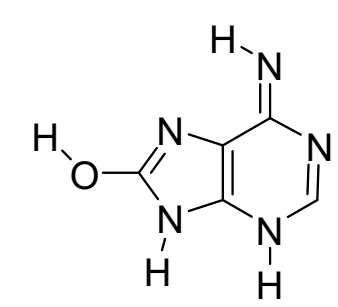
8OA_6imino8enol(e)
-542.617948

Figure S7. Structure of Deprotonated N9-anion of 8OA, and Protonated N7-cation of 8OA in the Gas Phase

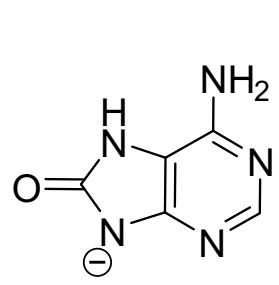
8OA_6amino8keto-N9H
-542.145513

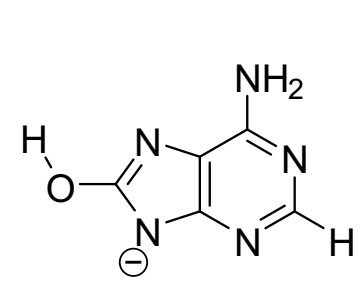
8OA_6amino8enol(a)-N9H
-542.133507

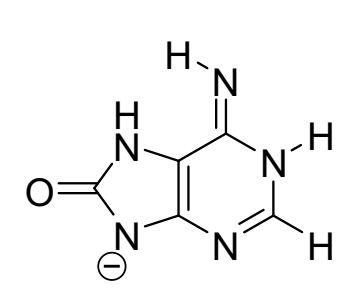
8OA_6imino8keto(b)-N9H
-542.126742

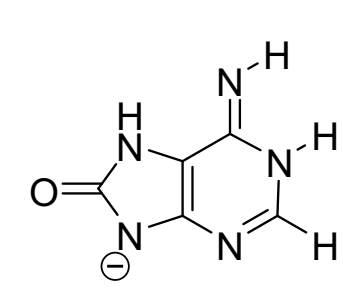
8OA_6imino8keto(a)-N9H
-542.121970

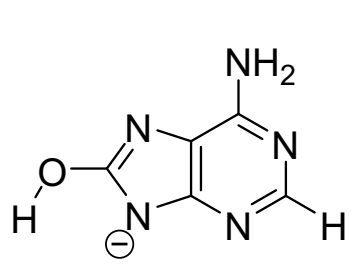
8OA_6amino8enol(b)-N9H
-542.134362

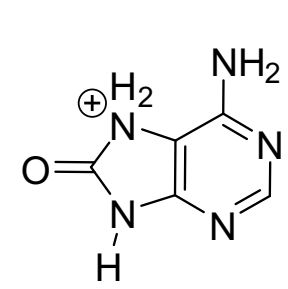
8OA+N7H
-542.985450

Figure S8. Structure of 8OA, Deprotonated N9-anion of 8OA, and Protonated N7-cation of 8OA in the Water Phase

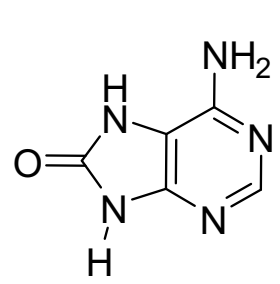

8OA_6amino8keto_water
-542.717658

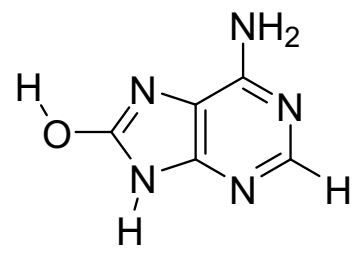

8OA_6amino8enol(a)_water
-542.698118

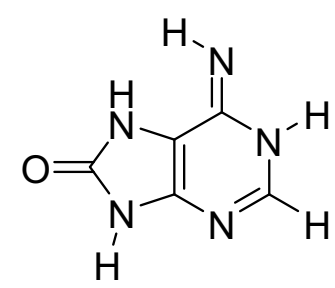

8OA_6imino8keto(b)_water
-542.698786

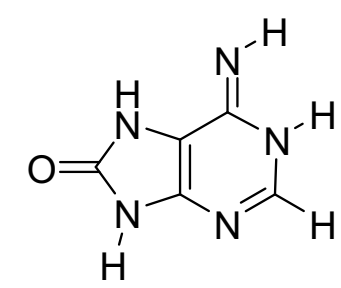

8OA_6imino8keto(a)_water
-542.698016

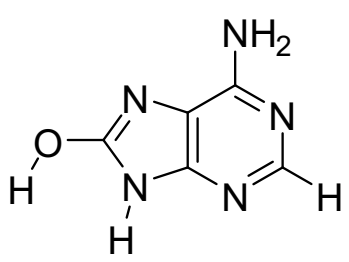

8OA_6amino8enol(b)_water
-542.695836

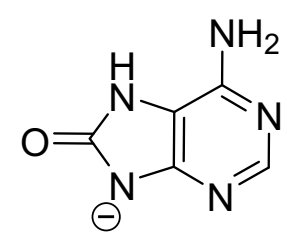

8OA_6amino8keto-N9H_water
-542.257934

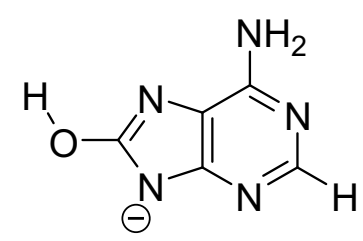

8OA_6amino8enol(a)-N9H_water
-542.240278

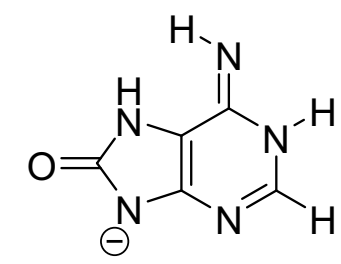

8OA_6imino8keto(b)-N9H_water
-542.239473

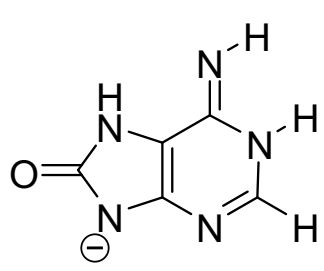

8OA_6imino8keto(a)-N9H_water
-542.238158

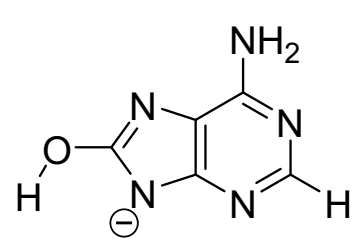

8OA_6amino8enol(b)-N9H_water
-542.239965

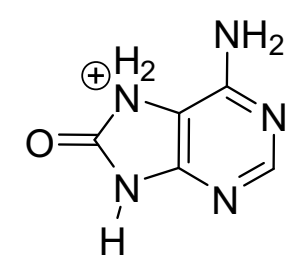

8OA+N7H_water
-618.391589

Calculated Structures (8-bromoadenine, 8BA)

Figure S9. Structure of 8BA, Deprotonated N9-anion of 8BA, and Protonated N7-cation of 8BA in the Gas Phase and Water Phase

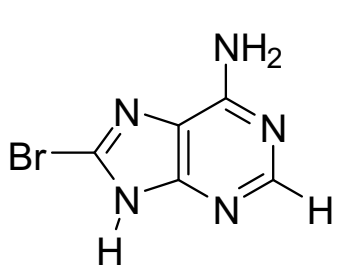

8BA
-3040.963778

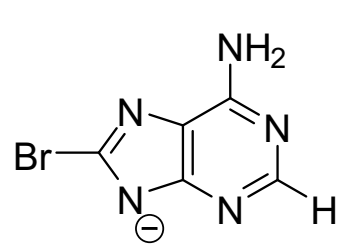

8BA-N9H
-3040.440863

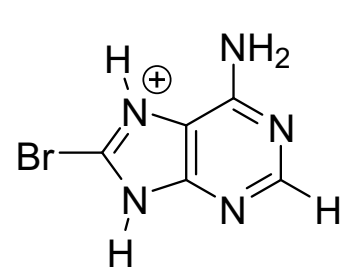

8BA+N7H
-3041.306008

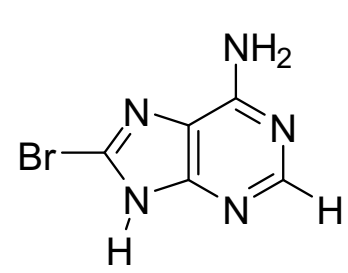

8BA_water
-3040.989059

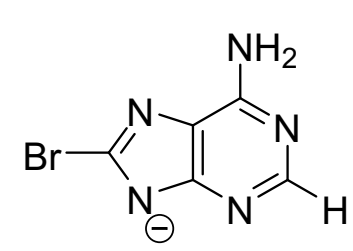

8BA-N9H_water
-3040.536416

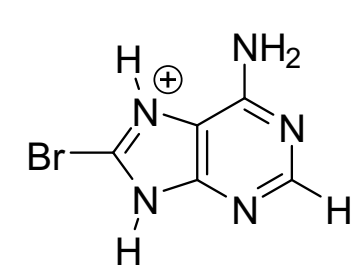

8BA+N7H_water
-3041.410345

Calculated Structures (Hypoxanthine, H)

Figure S10. Structure of H, Deprotonated N9-anion of H, and Protonated N7-cation of H in the Gas Phase and Water Phase

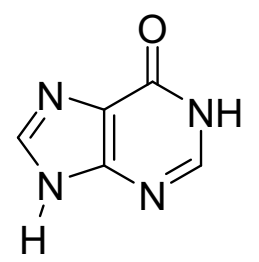
H
-487.294268

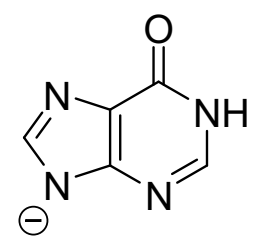
H-N9H
-486.764346

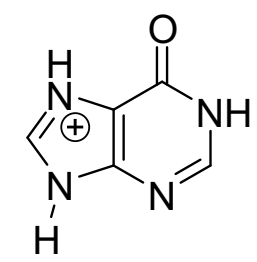
H+N7H
-487.646796

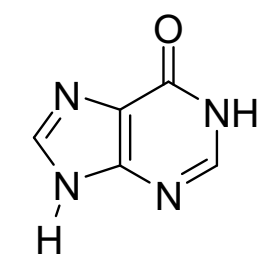
H_water
-487.329018

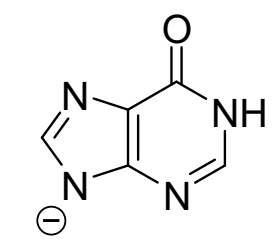
H-N9H_water
-486.871427

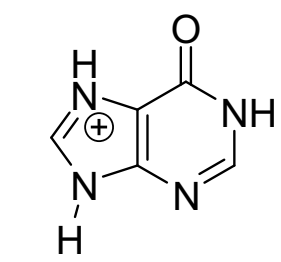
H+N7H_water
-487.761587

Calculated Structures (2-oxoadenine, 2OA)

Figure S11. Structure of 2OA, Deprotonated N9-anion of 2OA, and Protonated N7-cation of 2OA in the Gas Phase and Water Phase

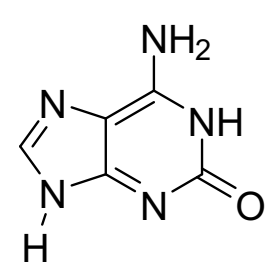
2OA
-542.666274

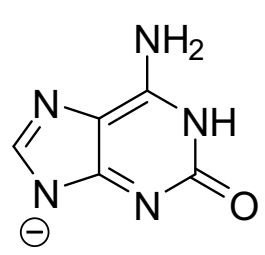
2OA-N9H
-542.130499

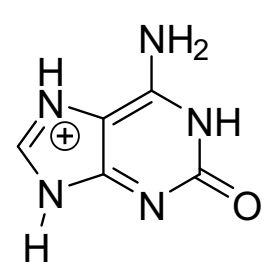
2OA+N7H
-543.009719

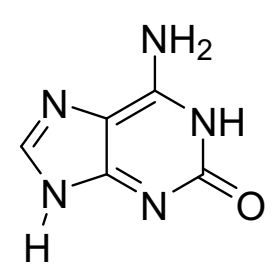
2OA_water
-542.713825

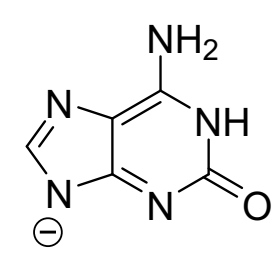
2OA-N9H_water
-542.250343

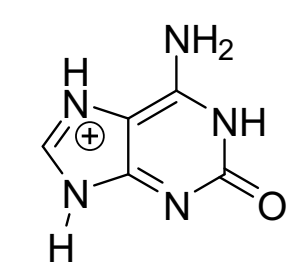
2OA+N7H_water
-543.146585

Calculated Structures (Guanine, G)

Figure S12. Structure of G, Deprotonated N9-anion of G, and Protonated N7-cation of G in the Gas Phase and Water Phase

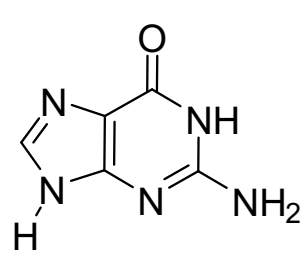
G
-542.671915

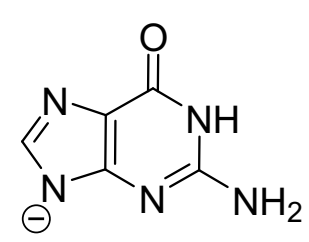
G-N9H
-542.135855

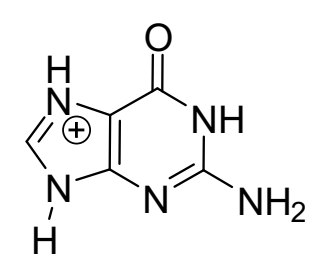
G+N7H
-543.037540

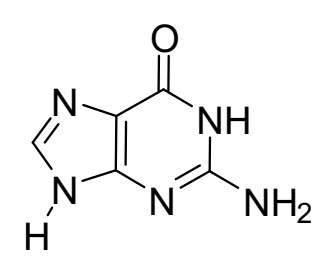
G_water
-542.714305

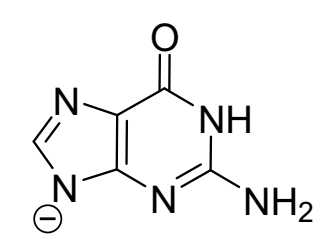
G-N9H_water
-542.252284

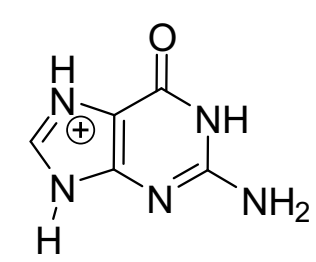
G+N7H_water
-543.151738

Calculated Structures (8-oxoguanine, 8OG)

Figure S13. Structure of 8OG, Deprotonated N9-anion of 8OG, and Protonated N7-cation of 8OG in the Gas Phase and Water Phase

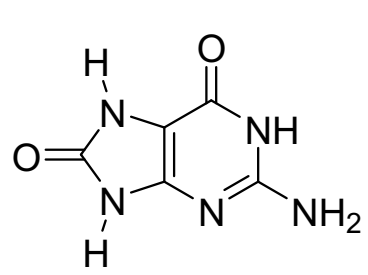
8OG
-617.951282

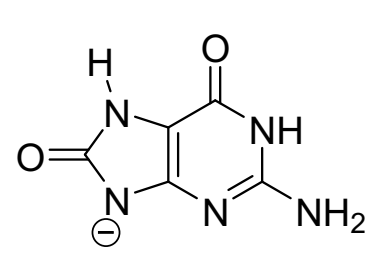
8OG-N9H
-617.413895

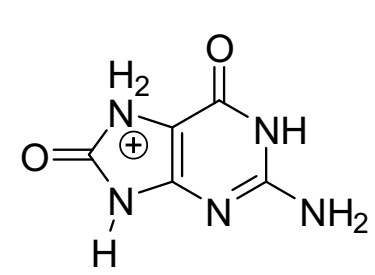
8OG+N7H
-618.266665

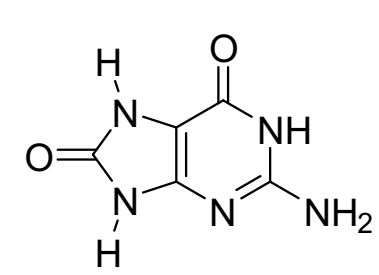
8OG_water
-617.993263

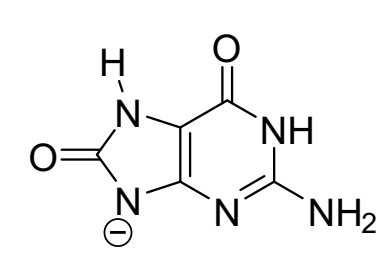
8OG-N9H_water
-617.534536

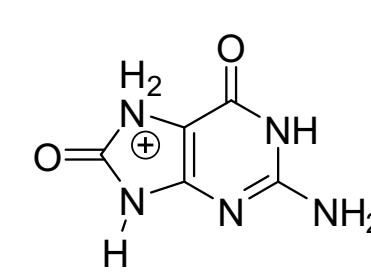
8OG+N7H_water
-467.87352

Calculated Structures (8-bromoguanine, 8BG)

Figure S14. Structure of 8BG, Deprotonated N9-anion of 8BG, and Protonated N7-cation of 8BG in the Gas Phase and Water Phase

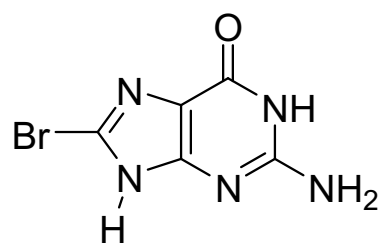

8BrG
-3116.224943

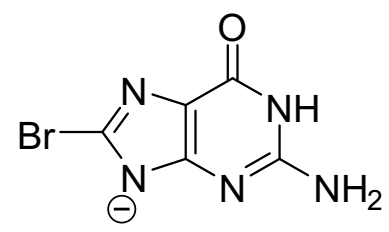

8BrG-9H
-3115.702872

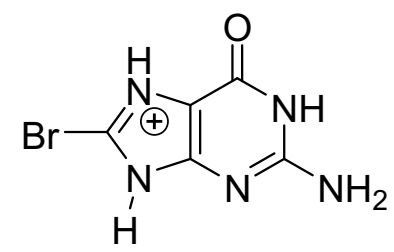

8BG+N7H
-3116.584673

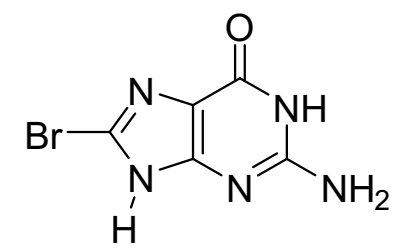

8BG_water
-3116.265114

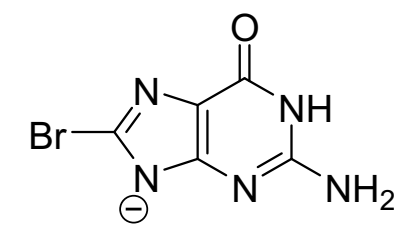

8BG-N9H_water
-3115.813042

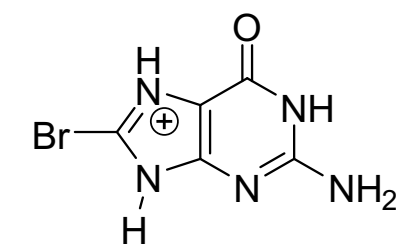

8BG+N7H_water
-3116.689727

Calculated Structures (Xanthine, X)

Figure S15. Structure of X, Deprotonated N9-anion of X, and Protonated N7-cation of X in the Gas Phase

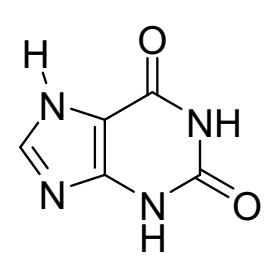

X_26diketoN7H
-562.577421

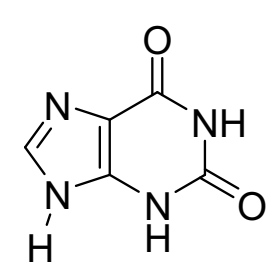

X_2keto6keto
-562.563791

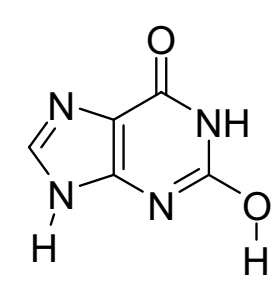

X_2enol6keto(a)
-562.556833

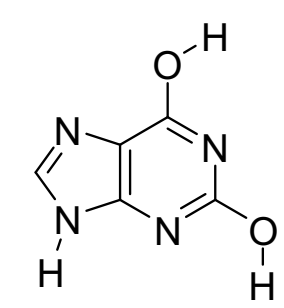

X_2enol6enol(c)
-562.550896

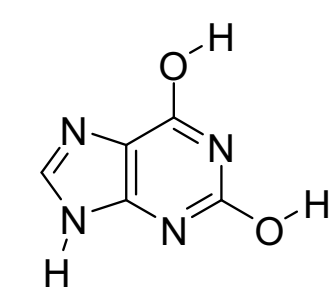

X_2enol6enol(d)
-562.550627

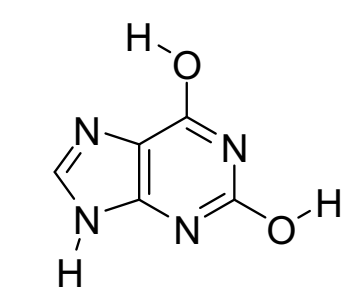

X_2enol6enol(b)
-562.550440

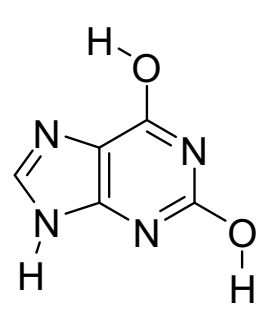

X_2enol6enol(a)
-562.549199

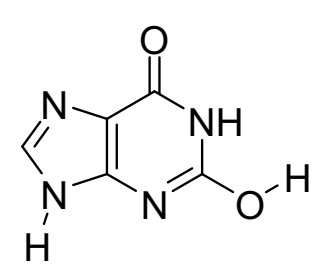

X_2enol6keto(b)
-562.547442

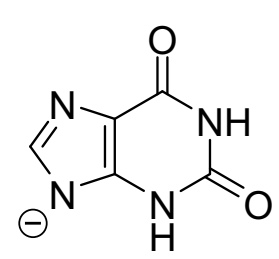

X_2keto6keto
-N9H
-562.055895

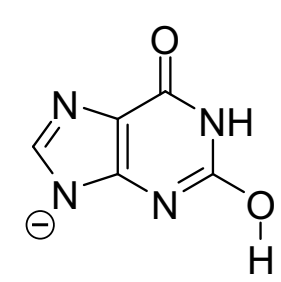

X_2enol6keto(a)
-N9H
-562.026968

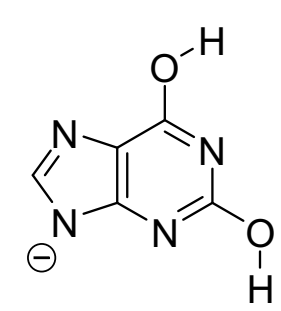

X_2enol6enol(c)
-N9H
-562.020868

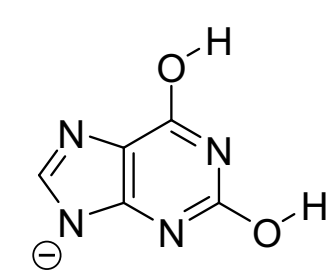

X_2enol6enol(d)
-N9H
-562.018482

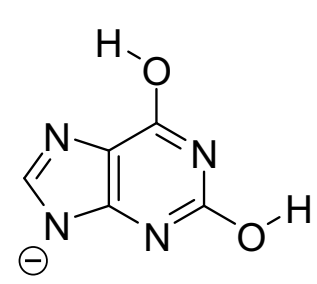

X_2enol6enol(b)
-N9H
-562.022113

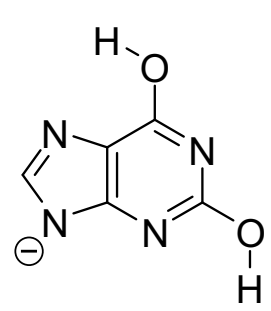

X_2enol6enol(a)
-N9H
-562.023107

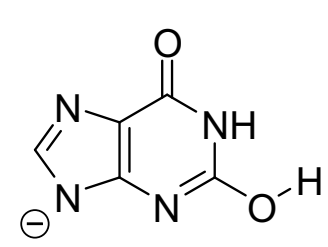

X_2enol6keto(b)
-N9H
-562.015663

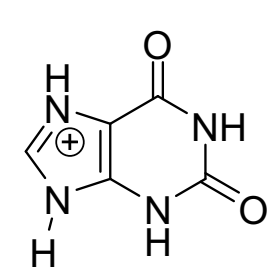

X+N7H
-562.908001

Figure S16. Structure of X, Deprotonated N9-anion of X, and Protonated N7-cation of X in the Water Phase

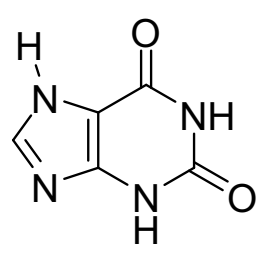

X_26diketoN7H_water
-562.609218

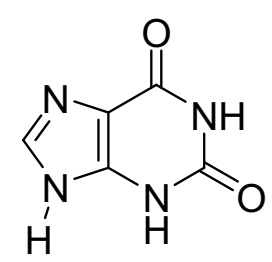

X_2keto6keto_water
-562.605445

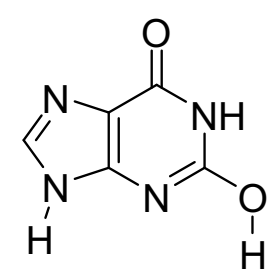

X_2enol6keto(a)_water
-562.594489

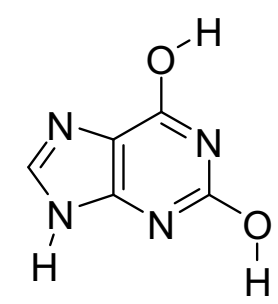

X_2enol6enol(c)_water
-562.584385

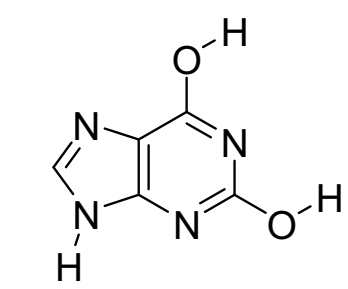

X_2enol6enol(d)_water
-562.584479

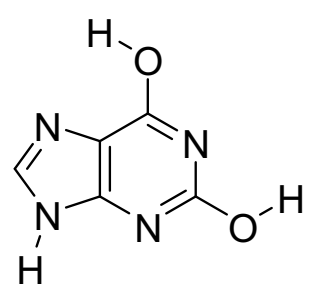

X_2enol6enol(b)_water
-562.583793

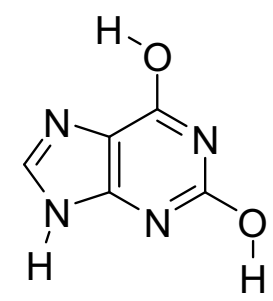

X_2enol6enol(a)_water
-562.583909

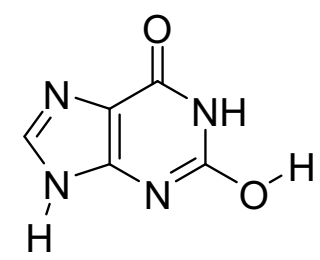

X_2enol6keto(b)_water
-562.591497

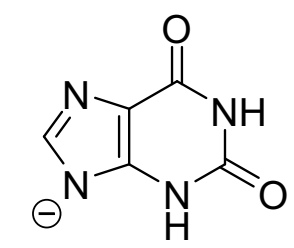

X_2keto6keto
-N9H_water
-562.154061

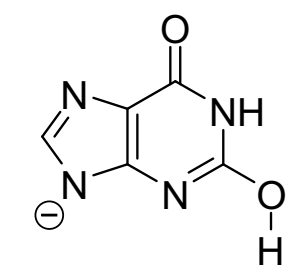

X_2enol6keto(a)
-N9H_water
-562.135831

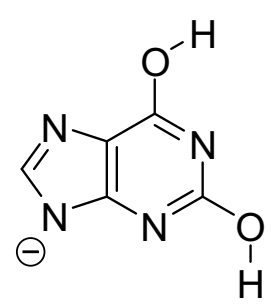

X_2enol6enol(c)
-N9H_water
-562.125396

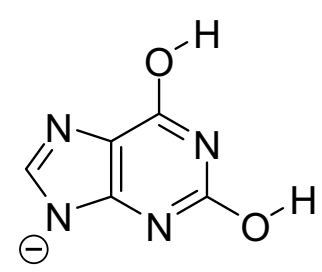

X_2enol6enol(d)
-N9H_water
-562.125158

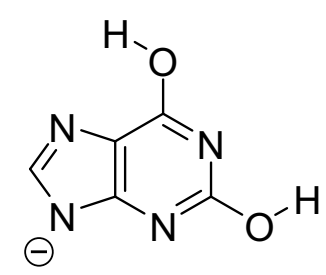

X_2enol6enol(b)
-N9H_water
-562.124854

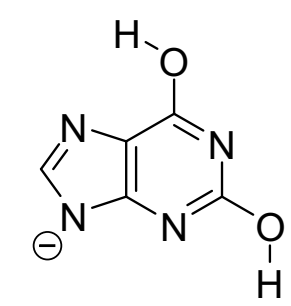

X_2enol6enol(a)
-N9H_water
-562.125357

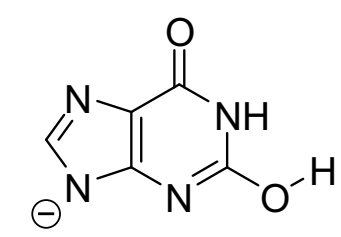

X_2enol6keto(b)
-N9H_water
-562.132401

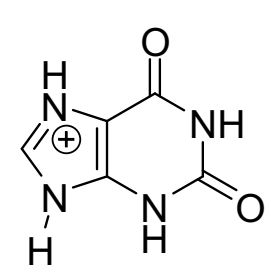

X+N7H_water
-563.035693

Figure S17. Structure of X-N3anion, Deprotonated N9-anion of X-N3anion in the Gas Phase and Water Phase

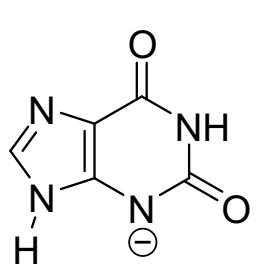

X_N3anion
-562.047146

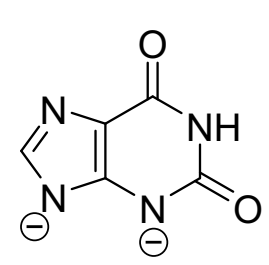

X_N3anion-N9H
-561.373444

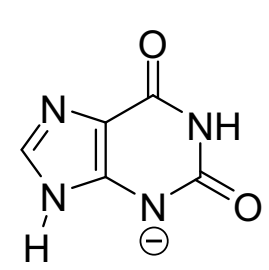

X_N3anion_water
-562.155880

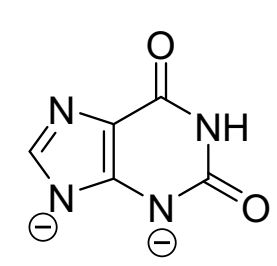

X_N3anion-N9H_water
-561.686613

Calculated Structures (Nebularine, N)

Figure S18. Structure of N, Deprotonated N9-anion of N, and Protonated N7-cation of N in the Gas Phase and Water Phase

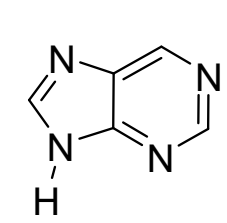

N
-412.025424

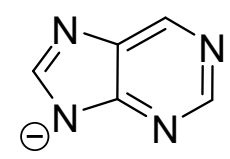

N-N9H
-411.496461

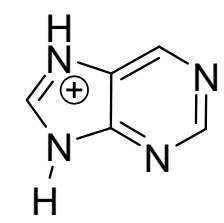

N+N7H
-412.367158

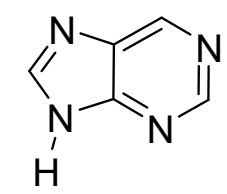

N_water
-412.049118

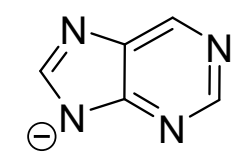

N-N9H_water
-411.591266

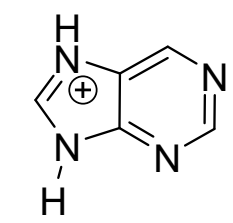

N+N7H_water
-412.481284

Calculated Structures (Thymine, T)

Figure S19. Structure of T and Deprotonated N9-anion of T in the Gas Phase and Water Phase

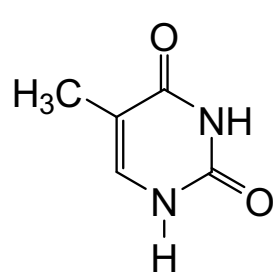

T
-454.229450

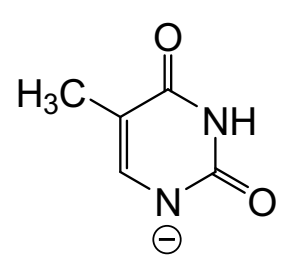

T-1H
-453.696474

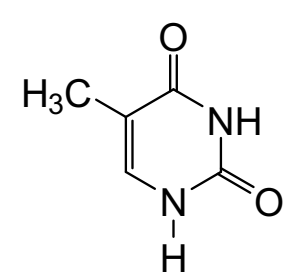

T_water
-454.252290

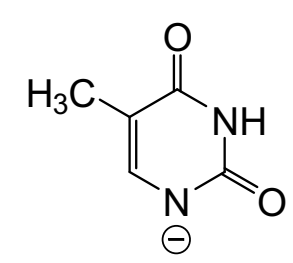

T_SMD_water
-453.793264

Calculated Structures (Uracil, U)

Figure S20. Structure of U and Deprotonated N9-anion of U in the Gas Phase and Water Phase

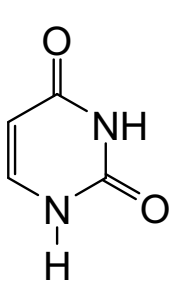

U
-414.919041

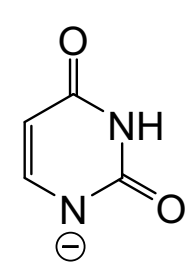

U-1H
-414.388650

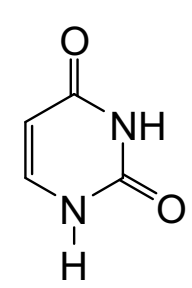

U_water
-414.943265

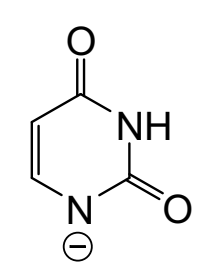

U-N1H_water
-414.485974

Calculated Structures (Ethenocytosine, εC)

Figure S21. Structure of εC and Deprotonated N9-anion of εC in the Gas Phase and Water Phase

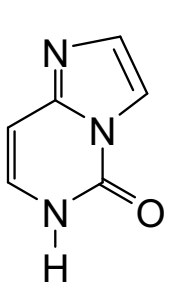

eC
-471.233003

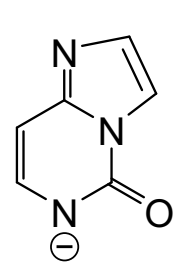

eC-1H
-470.701137

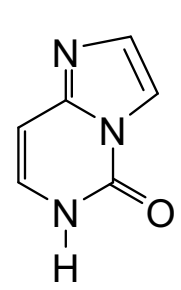

eC_water
-471.253015

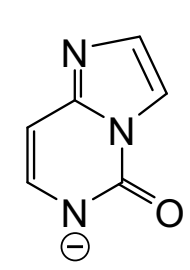

eC-N1H_water
-470.793780

Result for 2OA.log

```
SCF Done:  E(RB3LYP) =  -542.748767109     A.U. after    8 cycles

Zero-point correction=                           0.115703 (Hartree/Particle)
Thermal correction to Energy=                    0.124145
Thermal correction to Enthalpy=                  0.125090
Thermal correction to Gibbs Free Energy=         0.082493
Sum of electronic and zero-point Energies=           -542.633064
Sum of electronic and thermal Energies=              -542.624622
Sum of electronic and thermal Enthalpies=            -542.623677
Sum of electronic and thermal Free Energies=         -542.666274

                     E (Thermal)             CV                S
                      KCal/Mol        Cal/Mol-Kelvin    Cal/Mol-Kelvin
Total                   77.902             33.360            89.653

---------------------------------------------------------------------
Center     Atomic      Atomic             Coordinates (Angstroms)
Number     Number       Type             X           Y           Z
---------------------------------------------------------------------
     1          6           0        0.490345   -0.928231   -0.005043
     2          6           0        0.751286    0.459379   -0.007154
     3          6           0       -0.344915    1.299230   -0.005185
     4          6           0       -1.766473   -0.766049    0.002337
     5          6           0        2.669851   -0.451242    0.006447
     6          7           0       -1.542944    0.672615    0.005401
     7          7           0       -0.661841   -1.562089   -0.000145
     8          7           0       -0.266074    2.652068   -0.054196
     9          7           0        1.748289   -1.488653    0.000889
    10          7           0        2.114774    0.724843    0.003045
    11          1           0        0.651480    3.045179    0.093590
    12          1           0       -1.046061    3.207920    0.258193
    13          1           0        1.940239   -2.477765    0.006918
    14          1           0        3.732209   -0.640046    0.013696
    15          8           0       -2.927601   -1.125952    0.002537
    16          1           0       -2.403065    1.202315   -0.026052
---------------------------------------------------------------------
```

Result for 2OA-N9H.log

```
SCF Done:  E(RB3LYP) =  -542.200266335     A.U. after    9 cycles

Zero-point correction=                           0.102773 (Hartree/Particle)
Thermal correction to Energy=                    0.110825
Thermal correction to Enthalpy=                  0.111769
Thermal correction to Gibbs Free Energy=         0.069768
Sum of electronic and zero-point Energies=           -542.097493
Sum of electronic and thermal Energies=              -542.089441
Sum of electronic and thermal Enthalpies=            -542.088497
```

```
Sum of electronic and thermal Free Energies=        -542.130499

                    E (Thermal)             CV                S
                     KCal/Mol        Cal/Mol-Kelvin    Cal/Mol-Kelvin
Total                  69.544            31.362            88.400

---------------------------------------------------------------------
Center     Atomic      Atomic             Coordinates (Angstroms)
Number     Number       Type             X           Y           Z
---------------------------------------------------------------------
     1          6           0        0.520558   -1.001868   -0.006823
     2          6           0        0.798022    0.412593   -0.024527
     3          6           0       -0.255397    1.271966   -0.017819
     4          6           0       -1.758094   -0.713477    0.004651
     5          6           0        2.617813   -0.626034    0.011512
     6          7           0       -1.492097    0.705326    0.008704
     7          7           0       -0.705484   -1.550153    0.008801
     8          7           0       -0.153990    2.664128   -0.076206
     9          7           0        1.713106   -1.646594    0.014426
    10          7           0        2.166636    0.628137   -0.006699
    11          1           0        0.814919    2.937028    0.046414
    12          1           0       -0.747020    3.139444    0.594833
    13          1           0        3.681886   -0.836218    0.025537
    14          8           0       -2.958223   -1.022506   -0.002641
    15          1           0       -2.318598    1.274820   -0.090797
---------------------------------------------------------------------
```

Result for 2OA-N9H_water.log

```
SCF Done:  E(RB3LYP) =  -542.320497379     A.U. after    1 cycles


Zero-point correction=                           0.103031 (Hartree/Particle)
Thermal correction to Energy=                    0.111056
Thermal correction to Enthalpy=                  0.112000
Thermal correction to Gibbs Free Energy=         0.070154
Sum of electronic and zero-point Energies=           -542.217467
Sum of electronic and thermal Energies=              -542.209441
Sum of electronic and thermal Enthalpies=            -542.208497
Sum of electronic and thermal Free Energies=         -542.250343

                    E (Thermal)             CV                S
                     KCal/Mol        Cal/Mol-Kelvin    Cal/Mol-Kelvin
Total                  69.689            31.445            88.072

---------------------------------------------------------------------
Center     Atomic      Atomic             Coordinates (Angstroms)
Number     Number       Type             X           Y           Z
---------------------------------------------------------------------
     1          6           0        0.556858   -0.964793   -0.000558
     2          6           0        0.794969    0.430152   -0.002966
     3          6           0       -0.294033    1.279559   -0.004103
     4          6           0       -1.714124   -0.733792    0.000446
     5          6           0        2.650460   -0.571015    0.003002
```

6	7	0	-1.507982	0.661248	0.000166
7	7	0	-0.663984	-1.555990	-0.000409
8	7	0	-0.249574	2.628149	-0.053838
9	7	0	1.754591	-1.601916	0.003596
10	7	0	2.160983	0.667497	-0.000802
11	1	0	0.635420	3.066071	0.155645
12	1	0	-1.076378	3.151679	0.199545
13	1	0	3.715317	-0.760058	0.005917
14	8	0	-2.918655	-1.114790	0.002794
15	1	0	-2.348133	1.227043	0.000628

Result for 2OA_water.log

```
 SCF Done:  E(RB3LYP) =  -542.795644211     A.U. after    1 cycles

 Zero-point correction=                           0.115469 (Hartree/Particle)
 Thermal correction to Energy=                    0.124055
 Thermal correction to Enthalpy=                  0.125000
 Thermal correction to Gibbs Free Energy=         0.081819
 Sum of electronic and zero-point Energies=           -542.680175
 Sum of electronic and thermal Energies=              -542.671589
 Sum of electronic and thermal Enthalpies=            -542.670645
 Sum of electronic and thermal Free Energies=         -542.713825
```

	E (Thermal) KCal/Mol	CV Cal/Mol-Kelvin	S Cal/Mol-Kelvin
Total	77.846	33.105	90.882

Center Number	Atomic Number	Atomic Type	Coordinates (Angstroms) X	Y	Z
1	6	0	0.528928	-0.905822	0.000035
2	6	0	0.749417	0.475708	0.000016
3	6	0	-0.375529	1.296520	-0.000008
4	6	0	-1.723980	-0.773241	0.000004
5	6	0	2.685622	-0.408082	-0.000012
6	7	0	-1.560396	0.629913	0.000014
7	7	0	-0.633765	-1.562601	0.000057
8	7	0	-0.362691	2.626532	-0.000095
9	7	0	1.784754	-1.449650	-0.000014
10	7	0	2.109063	0.765545	-0.000022
11	1	0	0.518474	3.117127	0.000210
12	1	0	-1.220328	3.159540	0.000362
13	1	0	2.011758	-2.434991	0.000009
14	1	0	3.748817	-0.586018	-0.000019
15	8	0	-2.897767	-1.199864	-0.000059
16	1	0	-2.422091	1.164583	0.000114

Result for 8BA.log

```
 SCF Done:  E(RB3LYP) =  -3041.02979951     A.U. after    1 cycles


 Zero-point correction=                           0.101122 (Hartree/Particle)
 Thermal correction to Energy=                    0.109975
 Thermal correction to Enthalpy=                  0.110919
 Thermal correction to Gibbs Free Energy=         0.066021
 Sum of electronic and zero-point Energies=          -3040.928677
 Sum of electronic and thermal Energies=             -3040.919825
 Sum of electronic and thermal Enthalpies=           -3040.918881
 Sum of electronic and thermal Free Energies=        -3040.963778

                     E (Thermal)             CV                S
                      KCal/Mol        Cal/Mol-Kelvin    Cal/Mol-Kelvin
 Total                   69.010             33.274             94.495

 ---------------------------------------------------------------------
 Center     Atomic      Atomic             Coordinates (Angstroms)
 Number     Number       Type             X           Y           Z
 ---------------------------------------------------------------------
      1          6           0       -0.968550   -1.011451   -0.004154
      2          6           0       -1.082515    0.378047   -0.005512
      3          6           0       -2.387438    0.895122   -0.003329
      4          6           0       -3.156277   -1.279426    0.005847
      5          6           0        0.996965   -0.014177   -0.002165
      6          7           0       -3.414955    0.033463    0.006694
      7          7           0       -1.969233   -1.889302   -0.000226
      8          7           0       -2.642902    2.222846   -0.034209
      9          7           0        0.391066   -1.249958   -0.003281
     10          7           0        0.168680    0.983011   -0.003294
     11          1           0       -1.889372    2.874470    0.106675
     12          1           0       -3.592065    2.526183    0.109256
     13          1           0       -4.025499   -1.927810    0.012790
     14         35           0        2.871907    0.119018    0.001995
     15          1           0        0.848515   -2.147578   -0.002457
 ---------------------------------------------------------------------
```

Result for 8BA-N9H.log

```
 SCF Done:  E(RB3LYP) =  -3040.49455471     A.U. after    8 cycles


 Zero-point correction=                           0.088344 (Hartree/Particle)
 Thermal correction to Energy=                    0.096634
 Thermal correction to Enthalpy=                  0.097578
 Thermal correction to Gibbs Free Energy=         0.053691
 Sum of electronic and zero-point Energies=          -3040.406211
 Sum of electronic and thermal Energies=             -3040.397921
 Sum of electronic and thermal Enthalpies=           -3040.396976
 Sum of electronic and thermal Free Energies=        -3040.440863
```

	E (Thermal)	CV	S
	KCal/Mol	Cal/Mol-Kelvin	Cal/Mol-Kelvin
Total	60.639	31.264	92.368

Center Number	Atomic Number	Atomic Type	Coordinates (Angstroms) X	Y	Z
1	6	0	-0.914150	-1.041931	-0.013024
2	6	0	-1.069328	0.367284	-0.027014
3	6	0	-2.364387	0.876726	-0.017201
4	6	0	-3.136452	-1.285388	0.016618
5	6	0	0.951621	-0.105181	-0.008736
6	7	0	-3.405268	0.037367	0.012499
7	7	0	-1.961248	-1.896286	0.003588
8	7	0	-2.616798	2.241972	-0.070625
9	7	0	0.420975	-1.334336	-0.004021
10	7	0	0.171151	0.970369	-0.021443
11	1	0	-1.839087	2.806654	0.241887
12	1	0	-3.508575	2.499015	0.328145
13	1	0	-4.012991	-1.928668	0.037632
14	35	0	2.865575	0.103438	0.007100

Result for 8BA-N9H_water.log

```
SCF Done:  E(RB3LYP) =  -3040.58932948     A.U. after    1 cycles

Zero-point correction=                           0.087618 (Hartree/Particle)
Thermal correction to Energy=                    0.096031
Thermal correction to Enthalpy=                  0.096975

Thermal correction to Gibbs Free Energy=         0.052913
Sum of electronic and zero-point Energies=          -3040.501712
Sum of electronic and thermal Energies=             -3040.493299
Sum of electronic and thermal Enthalpies=           -3040.492355
Sum of electronic and thermal Free Energies=        -3040.536416
```

	E (Thermal)	CV	S
	KCal/Mol	Cal/Mol-Kelvin	Cal/Mol-Kelvin
Total	60.260	31.784	92.735

Center Number	Atomic Number	Atomic Type	Coordinates (Angstroms) X	Y	Z
1	6	0	-0.910556	-1.026389	0.000270
2	6	0	-1.067117	0.370808	-0.005951
3	6	0	-2.367033	0.883755	-0.006961
4	6	0	-3.129105	-1.296142	0.003805
5	6	0	0.963639	-0.098373	-0.001473
6	7	0	-3.400349	0.021600	0.000237

```
     7          7           0       -1.946325   -1.897823    0.004187
     8          7           0       -2.635283    2.223517   -0.063396
     9          7           0        0.422034   -1.327124    0.003379
    10          7           0        0.177594    0.971103   -0.007374
    11          1           0       -1.896610    2.844941    0.233536
    12          1           0       -3.564856    2.499850    0.221788
    13          1           0       -3.998803   -1.944147    0.009469
    14         35           0        2.862788    0.104528    0.001081
 ---------------------------------------------------------------------
```

Result for 8BA_water.log

```
 SCF Done:  E(RB3LYP) =  -3041.05448809     A.U. after    1 cycles

 Zero-point correction=                           0.100758 (Hartree/Particle)
 Thermal correction to Energy=                    0.109720
 Thermal correction to Enthalpy=                  0.110664
 Thermal correction to Gibbs Free Energy=         0.065429
 Sum of electronic and zero-point Energies=          -3040.953730
 Sum of electronic and thermal Energies=             -3040.944768
 Sum of electronic and thermal Enthalpies=           -3040.943824
 Sum of electronic and thermal Free Energies=        -3040.989059

                     E (Thermal)             CV                S
                      KCal/Mol        Cal/Mol-Kelvin    Cal/Mol-Kelvin
 Total                   68.851             33.331             95.206

 ---------------------------------------------------------------------
 Center     Atomic      Atomic             Coordinates (Angstroms)
 Number     Number       Type             X           Y           Z
 ---------------------------------------------------------------------
      1          6           0       -0.962866   -1.007931   -0.000338
      2          6           0       -1.082057    0.378149   -0.001353
      3          6           0       -2.385943    0.902696   -0.001763
      4          6           0       -3.154037   -1.280198    0.001787
      5          6           0        0.997948   -0.026067   -0.000380
      6          7           0       -3.416119    0.031576    0.001655
      7          7           0       -1.967775   -1.890421    0.000683
      8          7           0       -2.646207    2.220272   -0.027850
      9          7           0        0.390901   -1.254077    0.000368
     10          7           0        0.172559    0.979215   -0.001403
     11          1           0       -1.900525    2.887585    0.092336
     12          1           0       -3.598140    2.534070    0.087012
     13          1           0       -4.021784   -1.928838    0.004294
     14         35           0        2.870578    0.121788    0.000388
     15          1           0        0.838437   -2.161213    0.000874
 ---------------------------------------------------------------------
```

Result for 8BG.log

```
SCF Done:  E(RB3LYP) =  -3116.29457914     A.U. after    1 cycles


Zero-point correction=                           0.105957 (Hartree/Particle)
Thermal correction to Energy=                    0.115762
Thermal correction to Enthalpy=                  0.116707
Thermal correction to Gibbs Free Energy=         0.069637
Sum of electronic and zero-point Energies=         -3116.188622
Sum of electronic and thermal Energies=            -3116.178817
Sum of electronic and thermal Enthalpies=          -3116.177873
Sum of electronic and thermal Free Energies=       -3116.224943

                    E (Thermal)             CV                S
                     KCal/Mol        Cal/Mol-Kelvin    Cal/Mol-Kelvin
Total                  72.642            37.178             99.067

---------------------------------------------------------------------
Center     Atomic      Atomic             Coordinates (Angstroms)
Number     Number       Type             X           Y           Z
---------------------------------------------------------------------
      1          6           0       -0.665152    0.699831    0.007101
      2          6           0       -0.743015   -0.687786   -0.000181
      3          6           0       -2.957227   -0.797501   -0.003183
      4          6           0       -1.881139    1.460604    0.002733
      5          8           0       -2.074743    2.657219   -0.002941
      6          7           0        0.559216   -1.118952   -0.002747
      7          7           0       -1.839696   -1.477928    0.007333
      8          7           0       -3.004665    0.567087   -0.002757
      9          1           0       -3.895459    1.041173   -0.070677
     10          7           0        0.656978    1.115862    0.007214
     11          7           0       -4.155252   -1.464512   -0.067855
     12          1           0       -4.070489   -2.455305    0.105837
     13          1           0       -4.955497   -1.033020    0.369570
     14          6           0        1.340673    0.019317    0.001400
     15          1           0        0.878029   -2.074703   -0.009790
     16         35           0        3.216013   -0.121531   -0.000198
---------------------------------------------------------------------
```

Result for 8BG-N9H.log

```
SCF Done:  E(RB3LYP) =  -3115.75950657     A.U. after    1 cycles


Zero-point correction=                           0.092803 (Hartree/Particle)
Thermal correction to Energy=                    0.102310
Thermal correction to Enthalpy=                  0.103254
Thermal correction to Gibbs Free Energy=         0.056635
Sum of electronic and zero-point Energies=         -3115.666703
Sum of electronic and thermal Energies=            -3115.657197
Sum of electronic and thermal Enthalpies=          -3115.656252
Sum of electronic and thermal Free Energies=       -3115.702872

                    E (Thermal)             CV                S
                     KCal/Mol        Cal/Mol-Kelvin    Cal/Mol-Kelvin
```

	E (Thermal)	CV	S
Total	64.200	35.516	98.119

Center Number	Atomic Number	Atomic Type	Coordinates (Angstroms) X	Y	Z
1	6	0	-0.650974	0.694433	0.011834
2	6	0	-0.685194	-0.721154	-0.006547
3	6	0	-2.923638	-0.804481	-0.010695
4	6	0	-1.856133	1.448592	0.002953
5	8	0	-2.074932	2.658611	-0.008280
6	7	0	0.579336	-1.202225	-0.012158
7	7	0	-1.829792	-1.488552	0.000544
8	7	0	-2.981365	0.563351	-0.004813
9	1	0	-3.870009	1.027377	-0.124303
10	7	0	0.665621	1.105489	0.016109
11	7	0	-4.172214	-1.457598	-0.073944
12	1	0	-4.015181	-2.452882	0.031615
13	1	0	-4.818366	-1.133909	0.637886
14	6	0	1.289561	-0.056641	0.000939
15	35	0	3.212291	-0.134778	0.001428

Result for 8BG-N9H_water.log

```
SCF Done:  E(RB3LYP) =  -3115.86937189     A.U. after    1 cycles

Zero-point correction=                           0.092311 (Hartree/Particle)
Thermal correction to Energy=                    0.101749
Thermal correction to Enthalpy=                  0.102693
Thermal correction to Gibbs Free Energy=         0.056330
Sum of electronic and zero-point Energies=          -3115.777061
Sum of electronic and thermal Energies=             -3115.767623
Sum of electronic and thermal Enthalpies=           -3115.766679
Sum of electronic and thermal Free Energies=        -3115.813042
```

	E (Thermal) KCal/Mol	CV Cal/Mol-Kelvin	S Cal/Mol-Kelvin
Total	63.848	35.700	97.579

Center Number	Atomic Number	Atomic Type	Coordinates (Angstroms) X	Y	Z
1	6	0	-0.654258	0.676861	-0.002309
2	6	0	-0.690664	-0.726513	-0.001526
3	6	0	-2.936080	-0.802205	-0.003423
4	6	0	-1.855977	1.412258	-0.000154
5	8	0	-2.025896	2.651600	0.003480
6	7	0	0.580510	-1.205238	0.000423
7	7	0	-1.824046	-1.490217	0.002585
8	7	0	-2.975645	0.568679	-0.001974
9	1	0	-3.878541	1.029499	-0.011317

```
    10         7           0        0.664384    1.099793   -0.001073
    11         7           0       -4.150265   -1.435417   -0.074082
    12         1           0       -4.120592   -2.408504    0.198778
    13         1           0       -4.940614   -0.932694    0.309189
    14         6           0        1.295716   -0.059360   -0.000022
    15        35           0        3.203712   -0.133444    0.001114
 ---------------------------------------------------------------------
```

Result for 8BG_water.log

```
 SCF Done:  E(RB3LYP) =  -3116.33475588     A.U. after    1 cycles
```

```
 Zero-point correction=                           0.105816 (Hartree/Particle)
 Thermal correction to Energy=                    0.115547
 Thermal correction to Enthalpy=                  0.116491
 Thermal correction to Gibbs Free Energy=         0.069642
 Sum of electronic and zero-point Energies=          -3116.228940
 Sum of electronic and thermal Energies=             -3116.219209
 Sum of electronic and thermal Enthalpies=           -3116.218265
 Sum of electronic and thermal Free Energies=        -3116.265114

                     E (Thermal)             CV                S
                      KCal/Mol        Cal/Mol-Kelvin    Cal/Mol-Kelvin
 Total                   72.507             37.002             98.604

 ---------------------------------------------------------------------
 Center     Atomic      Atomic             Coordinates (Angstroms)
 Number     Number       Type             X           Y           Z
 ---------------------------------------------------------------------
      1          6           0       -0.671023    0.683347   -0.002032
      2          6           0       -0.742064   -0.704702   -0.000519
      3          6           0       -2.962925   -0.791944   -0.001924
      4          6           0       -1.879440    1.426498    0.000125
      5          8           0       -2.034736    2.654765    0.002321
      6          7           0        0.561238   -1.129877    0.001029
      7          7           0       -1.837529   -1.483306    0.002491
      8          7           0       -2.995686    0.577665    0.001180
      9          1           0       -3.899324    1.039848   -0.002374
     10          7           0        0.652572    1.108758   -0.001774
     11          7           0       -4.151361   -1.429277   -0.055010
     12          1           0       -4.141777   -2.417478    0.152569
     13          1           0       -4.988312   -0.923487    0.199003
     14          6           0        1.335485    0.005512   -0.000167
     15          1           0        0.882232   -2.089198    0.001456
     16         35           0        3.209721   -0.116223    0.000642
 ---------------------------------------------------------------------
```

Result for 8OA_6amino8enol(a).log

```
 SCF Done:  E(RB3LYP) =  -542.750140504     A.U. after    2 cycles
```

```
Zero-point correction=                           0.116108 (Hartree/Particle)
Thermal correction to Energy=                    0.124482
Thermal correction to Enthalpy=                  0.125427
Thermal correction to Gibbs Free Energy=         0.083090
Sum of electronic and zero-point Energies=          -542.634033
Sum of electronic and thermal Energies=             -542.625658
Sum of electronic and thermal Enthalpies=           -542.624714
Sum of electronic and thermal Free Energies=        -542.667051

                    E (Thermal)             CV                S
                     KCal/Mol        Cal/Mol-Kelvin    Cal/Mol-Kelvin
Total                  78.114             33.318            89.106

---------------------------------------------------------------------
Center     Atomic      Atomic             Coordinates (Angstroms)
Number     Number       Type             X           Y           Z
---------------------------------------------------------------------
     1          6           0        0.120339   -0.964645   -0.003673
     2          6           0       -0.099481    0.412314   -0.006463
     3          6           0       -1.436815    0.821225   -0.006043
     4          6           0       -2.040828   -1.403662    0.005039
     5          6           0        2.007505    0.170761    0.001162
     6          7           0       -2.397345   -0.116605    0.005238
     7          7           0       -0.806317   -1.915872   -0.000061
     8          7           0       -1.802729    2.131184   -0.050030
     9          7           0        1.499571   -1.102756   -0.000388
    10          7           0        1.108082    1.111200   -0.001734
    11          1           0       -1.107782    2.826871    0.166340
    12          1           0       -2.764964    2.341636    0.161643
    13          1           0       -2.855254   -2.119471    0.012630
    14          8           0        3.335220    0.332764    0.005692
    15          1           0        3.507733    1.284857    0.001270
    16          1           0        2.025345   -1.962019    0.001280
---------------------------------------------------------------------
```

Result for 8OA_6amino8enol(a)-N9H.log

```
SCF Done:  E(RB3LYP) =  -542.204231143     A.U. after    1 cycles


Zero-point correction=                           0.103415 (Hartree/Particle)
Thermal correction to Energy=                    0.111282
Thermal correction to Enthalpy=                  0.112227
Thermal correction to Gibbs Free Energy=         0.070724
Sum of electronic and zero-point Energies=          -542.100816
Sum of electronic and thermal Energies=             -542.092949
Sum of electronic and thermal Enthalpies=           -542.092005
Sum of electronic and thermal Free Energies=        -542.133507

                    E (Thermal)             CV                S
                     KCal/Mol        Cal/Mol-Kelvin    Cal/Mol-Kelvin
Total                  69.831             31.184            87.350
```

```
 ---------------------------------------------------------------------
 Center     Atomic      Atomic             Coordinates (Angstroms)
 Number     Number       Type             X           Y           Z
 ---------------------------------------------------------------------
      1          6           0        0.183710   -1.000769   -0.008256
      2          6           0       -0.069147    0.399558   -0.024189
      3          6           0       -1.391158    0.813577   -0.020753
      4          6           0       -2.022264   -1.391456    0.010466
      5          6           0        1.996990    0.052006    0.002055
      6          7           0       -2.378784   -0.094818    0.003701
      7          7           0       -0.802742   -1.919585    0.004098
      8          7           0       -1.746326    2.165478   -0.075153
      9          7           0        1.534324   -1.205307    0.005816
     10          7           0        1.134252    1.075961   -0.014498
     11          1           0       -1.014589    2.771636    0.271431
     12          1           0       -2.647034    2.341076    0.349638
     13          1           0       -2.850512   -2.096414    0.027625
     14          8           0        3.341004    0.292307    0.015794
     15          1           0        3.410251    1.255645    0.001260
 ---------------------------------------------------------------------
```

Result for 8OA_6amino8enol(a)-N9H_water.log

```
 SCF Done:  E(RB3LYP) =  -542.309924089     A.U. after    1 cycles


 Zero-point correction=                           0.102511 (Hartree/Particle)
 Thermal correction to Energy=                    0.110593
 Thermal correction to Enthalpy=                  0.111537
 Thermal correction to Gibbs Free Energy=         0.069646
 Sum of electronic and zero-point Energies=           -542.207413
 Sum of electronic and thermal Energies=              -542.199331
 Sum of electronic and thermal Enthalpies=            -542.198387
 Sum of electronic and thermal Free Energies=         -542.240278

                     E (Thermal)             CV                S
                      KCal/Mol        Cal/Mol-Kelvin    Cal/Mol-Kelvin
 Total                   69.398             31.816             88.166

 ---------------------------------------------------------------------
 Center     Atomic      Atomic             Coordinates (Angstroms)
 Number     Number       Type             X           Y           Z
 ---------------------------------------------------------------------
      1          6           0        0.184591   -0.981561    0.001198
      2          6           0       -0.073990    0.404703   -0.006619
      3          6           0       -1.401567    0.817882   -0.008661
      4          6           0       -2.012737   -1.407341    0.002887
      5          6           0        2.001420    0.078122    0.000437
      6          7           0       -2.375739   -0.116320   -0.001957
      7          7           0       -0.784978   -1.921158    0.004845
      8          7           0       -1.773564    2.141350   -0.069751
      9          7           0        1.535943   -1.185217    0.006038
     10          7           0        1.132442    1.088432   -0.007459
```

```
    11          1           0        -1.084651    2.803145    0.260231
    12          1           0        -2.712349    2.334141    0.253428
    13          1           0        -2.831175   -2.119151    0.008361
    14          8           0         3.349118    0.278493    0.002980
    15          1           0         3.510206    1.233475   -0.003329
 ---------------------------------------------------------------------
```

Result for 8OA_6amino8enol(a)_water.log

```
 SCF Done:  E(RB3LYP) =  -542.780144486     A.U. after    1 cycles

 Zero-point correction=                           0.115184 (Hartree/Particle)
 Thermal correction to Energy=                    0.123691
 Thermal correction to Enthalpy=                  0.124635
 Thermal correction to Gibbs Free Energy=         0.082027
 Sum of electronic and zero-point Energies=           -542.664960
 Sum of electronic and thermal Energies=              -542.656454
 Sum of electronic and thermal Enthalpies=            -542.655509
 Sum of electronic and thermal Free Energies=         -542.698118

                     E (Thermal)             CV                S
                      KCal/Mol        Cal/Mol-Kelvin    Cal/Mol-Kelvin
 Total                   77.617             33.587             89.677

 ---------------------------------------------------------------------
 Center     Atomic      Atomic             Coordinates (Angstroms)
 Number     Number       Type             X           Y           Z
 ---------------------------------------------------------------------
      1          6           0         0.129728   -0.958577    0.000928
      2          6           0        -0.103264    0.414511   -0.003129
      3          6           0        -1.440624    0.822573   -0.004801
      4          6           0        -2.034362   -1.412137    0.001801
      5          6           0         2.008550    0.177241    0.000116
      6          7           0        -2.400047   -0.127294   -0.000313
      7          7           0        -0.798125   -1.918341    0.002788
      8          7           0        -1.814630    2.125475   -0.052980
      9          7           0         1.501164   -1.093221    0.003290
     10          7           0         1.101327    1.119316   -0.003866
     11          1           0        -1.130424    2.825133    0.194023
     12          1           0        -2.775454    2.336625    0.176050
     13          1           0        -2.844203   -2.131737    0.005360
     14          8           0         3.334601    0.329976    0.001097
     15          1           0         3.546826    1.277093   -0.003282
     16          1           0         2.038448   -1.950123    0.007152
 ---------------------------------------------------------------------
```

Result for 8OA_6amino8enol(b).log

```
 SCF Done:  E(RB3LYP) =  -542.740181825     A.U. after    1 cycles
```

```
Zero-point correction=                           0.115165 (Hartree/Particle)
Thermal correction to Energy=                    0.124045
Thermal correction to Enthalpy=                  0.124990
Thermal correction to Gibbs Free Energy=         0.081414
Sum of electronic and zero-point Energies=           -542.625017
Sum of electronic and thermal Energies=              -542.616136
Sum of electronic and thermal Enthalpies=            -542.615192
Sum of electronic and thermal Free Energies=         -542.658768

                    E (Thermal)             CV                S
                     KCal/Mol        Cal/Mol-Kelvin    Cal/Mol-Kelvin
Total                  77.840             34.040             91.713

---------------------------------------------------------------------
Center     Atomic      Atomic             Coordinates (Angstroms)
Number     Number       Type             X           Y           Z
---------------------------------------------------------------------
     1          6           0        0.131344   -0.953029   -0.002339
     2          6           0       -0.109107    0.417905   -0.006200
     3          6           0       -1.454132    0.805710   -0.004236
     4          6           0       -2.020821   -1.429471    0.003563
     5          6           0        2.003115    0.228709    0.000211
     6          7           0       -2.398928   -0.148436    0.005713
     7          7           0       -0.777922   -1.921762    0.000173
     8          7           0       -1.836769    2.107888   -0.043118
     9          7           0        1.513541   -1.066232    0.006561
    10          7           0        1.080993    1.140335    0.002037
    11          1           0       -1.147482    2.817226    0.144751
    12          1           0       -2.803972    2.312414    0.148536
    13          1           0       -2.823386   -2.158624    0.007980
    14          8           0        3.317444    0.503095   -0.024024
    15          1           0        3.837896   -0.269279    0.224879
    16          1           0        2.028588   -1.927989   -0.079515
---------------------------------------------------------------------
```

Result for 8OA_6amino8enol(b)-N9H.log

```
SCF Done:  E(RB3LYP) =  -542.205137433     A.U. after    1 cycles
```

```
Zero-point correction=                           0.103456 (Hartree/Particle)
Thermal correction to Energy=                    0.111316
Thermal correction to Enthalpy=                  0.112260
Thermal correction to Gibbs Free Energy=         0.070775
Sum of electronic and zero-point Energies=           -542.101681
Sum of electronic and thermal Energies=              -542.093822
Sum of electronic and thermal Enthalpies=            -542.092878
Sum of electronic and thermal Free Energies=         -542.134362

                    E (Thermal)             CV                S
                     KCal/Mol        Cal/Mol-Kelvin    Cal/Mol-Kelvin
Total                  69.852             31.172             87.311
```

Center Number	Atomic Number	Atomic Type	Coordinates (Angstroms) X	Y	Z
1	6	0	0.214572	-0.973644	-0.010904
2	6	0	-0.087499	0.416060	-0.024304
3	6	0	-1.424286	0.780941	-0.019872
4	6	0	-1.974168	-1.446267	0.012093
5	6	0	1.984039	0.168324	0.000792
6	7	0	-2.377871	-0.163968	0.006291
7	7	0	-0.735794	-1.930080	0.002401
8	7	0	-1.825625	2.117809	-0.075198
9	7	0	1.571469	-1.117265	0.002542
10	7	0	1.085291	1.147309	-0.014071
11	1	0	-1.107505	2.750898	0.251398
12	1	0	-2.726752	2.269191	0.357291
13	1	0	-2.775811	-2.181348	0.031404
14	8	0	3.321540	0.442555	0.016646
15	1	0	3.739511	-0.428302	0.026154

Result for 8OA_6amino8enol(b)-N9H_water.log

```
SCF Done:  E(RB3LYP) =  -542.309878032     A.U. after    7 cycles

Zero-point correction=                           0.102685 (Hartree/Particle)
Thermal correction to Energy=                    0.110688
Thermal correction to Enthalpy=                  0.111633
Thermal correction to Gibbs Free Energy=         0.069913
Sum of electronic and zero-point Energies=           -542.207193
Sum of electronic and thermal Energies=              -542.199190
Sum of electronic and thermal Enthalpies=            -542.198245
Sum of electronic and thermal Free Energies=         -542.239965
```

	E (Thermal) KCal/Mol	CV Cal/Mol-Kelvin	S Cal/Mol-Kelvin
Total	69.458	31.681	87.807

Center Number	Atomic Number	Atomic Type	Coordinates (Angstroms) X	Y	Z
1	6	0	0.207248	-0.962346	0.000820
2	6	0	-0.087071	0.416634	-0.006715
3	6	0	-1.425627	0.793626	-0.008578
4	6	0	-1.977213	-1.447289	0.003248
5	6	0	1.994488	0.151649	0.000136
6	7	0	-2.374265	-0.166389	-0.001546
7	7	0	-0.736016	-1.928104	0.004682
8	7	0	-1.832743	2.106597	-0.069851
9	7	0	1.564245	-1.126610	0.005514
10	7	0	1.098547	1.135257	-0.007521
11	1	0	-1.160886	2.786617	0.258282

12	1	0	-2.775842	2.274983	0.254319
13	1	0	-2.776279	-2.180755	0.009005
14	8	0	3.323022	0.454609	0.002443
15	1	0	3.819499	-0.376617	0.006442

Result for 8OA_6amino8enol(b)_water.log

```
SCF Done:  E(RB3LYP) =  -542.778140750     A.U. after    1 cycles

Zero-point correction=                           0.115393 (Hartree/Particle)
Thermal correction to Energy=                    0.123843
Thermal correction to Enthalpy=                  0.124787
Thermal correction to Gibbs Free Energy=         0.082304
Sum of electronic and zero-point Energies=           -542.662748
Sum of electronic and thermal Energies=              -542.654298
Sum of electronic and thermal Enthalpies=            -542.653353
Sum of electronic and thermal Free Energies=         -542.695836
```

	E (Thermal) KCal/Mol	CV Cal/Mol-Kelvin	S Cal/Mol-Kelvin
Total	77.713	33.473	89.413

Center Number	Atomic Number	Atomic Type	Coordinates (Angstroms) X	Y	Z
1	6	0	0.144342	-0.946066	0.000735
2	6	0	-0.112801	0.422039	-0.003053
3	6	0	-1.458300	0.804661	-0.004725
4	6	0	-2.010082	-1.441048	0.002101
5	6	0	2.003942	0.228175	-0.000050
6	7	0	-2.399500	-0.163019	0.000023
7	7	0	-0.764689	-1.923853	0.002631
8	7	0	-1.856473	2.099963	-0.052636
9	7	0	1.518239	-1.054627	0.002904
10	7	0	1.076520	1.149362	-0.003764
11	1	0	-1.185136	2.813074	0.190972
12	1	0	-2.821459	2.293937	0.173847
13	1	0	-2.806279	-2.175724	0.005750
14	8	0	3.309860	0.513387	0.000902
15	1	0	3.845878	-0.294605	0.001258
16	1	0	2.066820	-1.905127	0.006806

Result for 8OA_6amino8keto.log

```
SCF Done:  E(RB3LYP) =  -542.766249578     A.U. after    8 cycles
```

```
Zero-point correction=                           0.116165 (Hartree/Particle)
Thermal correction to Energy=                    0.124568
Thermal correction to Enthalpy=                  0.125512
Thermal correction to Gibbs Free Energy=         0.082975
Sum of electronic and zero-point Energies=          -542.650084
Sum of electronic and thermal Energies=             -542.641682
Sum of electronic and thermal Enthalpies=           -542.640738
Sum of electronic and thermal Free Energies=        -542.683274

                    E (Thermal)             CV                S
                     KCal/Mol        Cal/Mol-Kelvin    Cal/Mol-Kelvin
Total                  78.167             33.151            89.526

---------------------------------------------------------------------
Center     Atomic      Atomic             Coordinates (Angstroms)
Number     Number       Type             X           Y           Z
---------------------------------------------------------------------
     1          6           0        0.180164   -0.950871    0.016674
     2          6           0       -0.106915    0.413752   -0.005398
     3          6           0       -1.444657    0.779801   -0.007282
     4          6           0       -1.978430   -1.458398   -0.020384
     5          6           0        2.169950    0.179947    0.003726
     6          7           0       -2.376256   -0.190216   -0.024753
     7          7           0       -0.720986   -1.920091    0.011163
     8          7           0       -1.876864    2.086813   -0.048950
     9          7           0        1.552431   -1.074258    0.042410
    10          7           0        1.112325    1.092493    0.025255
    11          1           0       -1.302160    2.777329    0.410489
    12          1           0       -2.865349    2.187565    0.135483
    13          1           0        2.070092   -1.938080    0.034297
    14          1           0       -2.762883   -2.206307   -0.036799
    15          8           0        3.353887    0.421252   -0.038449
    16          1           0        1.273992    2.060909   -0.195772
---------------------------------------------------------------------
```

Result for 8OA_6amino8keto-N9H.log

```
SCF Done:  E(RB3LYP) =  -542.215567769     A.U. after    1 cycles


Zero-point correction=                           0.102937 (Hartree/Particle)
Thermal correction to Energy=                    0.110942
Thermal correction to Enthalpy=                  0.111886
Thermal correction to Gibbs Free Energy=         0.070054
Sum of electronic and zero-point Energies=          -542.112631
Sum of electronic and thermal Energies=             -542.104626
Sum of electronic and thermal Enthalpies=           -542.103682
Sum of electronic and thermal Free Energies=        -542.145513

                    E (Thermal)             CV                S
                     KCal/Mol        Cal/Mol-Kelvin    Cal/Mol-Kelvin
Total                  69.617             31.366            88.042

---------------------------------------------------------------------
```

```
 Center     Atomic      Atomic             Coordinates (Angstroms)
 Number     Number       Type             X           Y           Z
 ---------------------------------------------------------------------
      1          6           0       -0.253871   -0.982376    0.019873
      2          6           0        0.081626    0.400909   -0.016307
      3          6           0        1.400371    0.771315   -0.022820
      4          6           0        1.953392   -1.447668   -0.021066
      5          6           0       -2.166248    0.068603    0.011161
      6          7           0        2.364609   -0.176289   -0.037862
      7          7           0        0.710760   -1.926844    0.017979
      8          7           0        1.822362    2.116780   -0.058773
      9          7           0       -1.589227   -1.172045    0.051771
     10          7           0       -1.129184    1.071182    0.056732
     11          1           0        2.748304   -2.190460   -0.034105
     12          8           0       -3.354336    0.382360   -0.049952
     13          1           0        1.250306    2.718292    0.522436
     14          1           0        2.796655    2.177155    0.212713
     15          1           0       -1.307436    1.981957   -0.335408
 ---------------------------------------------------------------------
```

Result for 8OA_6amino8keto-N9H_water.log

```
 SCF Done:  E(RB3LYP) =  -542.327752862     A.U. after    8 cycles

 Zero-point correction=                           0.102910 (Hartree/Particle)
 Thermal correction to Energy=                    0.111047
 Thermal correction to Enthalpy=                  0.111991
 Thermal correction to Gibbs Free Energy=         0.069819
 Sum of electronic and zero-point Energies=           -542.224843
 Sum of electronic and thermal Energies=              -542.216706
 Sum of electronic and thermal Enthalpies=            -542.215762
 Sum of electronic and thermal Free Energies=         -542.257934

                     E (Thermal)             CV                S
                      KCal/Mol        Cal/Mol-Kelvin    Cal/Mol-Kelvin
 Total                  69.683             31.583             88.759

 ---------------------------------------------------------------------
 Center     Atomic      Atomic             Coordinates (Angstroms)
 Number     Number       Type             X           Y           Z
 ---------------------------------------------------------------------
      1          6           0       -0.235477   -0.973552    0.002901
      2          6           0        0.081255    0.396236   -0.004414
      3          6           0        1.405195    0.788389   -0.007557
      4          6           0        1.960680   -1.447435    0.001670
      5          6           0       -2.139923    0.076378    0.000630
      6          7           0        2.358684   -0.172853   -0.003570
      7          7           0        0.716204   -1.928642    0.005529
      8          7           0        1.810522    2.103724   -0.072113
      9          7           0       -1.583603   -1.167853    0.006836
     10          7           0       -1.139626    1.048194   -0.006141
     11          1           0        2.756766   -2.183998    0.005642
     12          8           0       -3.365575    0.359650    0.000514
```

13	1	0	1.151911	2.783565	0.284791
14	1	0	2.757503	2.262247	0.247059
15	1	0	-1.307230	2.042895	-0.014770

Result for 8OA_6amino8keto_water.log

```
SCF Done:  E(RB3LYP) =  -542.799975659     A.U. after    7 cycles
```

```
Zero-point correction=                           0.115540 (Hartree/Particle)
Thermal correction to Energy=                    0.124018
Thermal correction to Enthalpy=                  0.124962
Thermal correction to Gibbs Free Energy=         0.082318
Sum of electronic and zero-point Energies=          -542.684436
Sum of electronic and thermal Energies=             -542.675958
Sum of electronic and thermal Enthalpies=           -542.675014
Sum of electronic and thermal Free Energies=        -542.717658
```

	E (Thermal) KCal/Mol	CV Cal/Mol-Kelvin	S Cal/Mol-Kelvin
Total	77.822	33.366	89.752

Center Number	Atomic Number	Atomic Type	Coordinates (Angstroms) X	Y	Z
1	6	0	0.177269	-0.952160	0.002253
2	6	0	-0.106742	0.406490	-0.000417
3	6	0	-1.442909	0.792553	-0.004026
4	6	0	-1.983926	-1.452743	0.001340
5	6	0	2.145557	0.173510	0.000113
6	7	0	-2.380135	-0.181951	-0.001019
7	7	0	-0.731332	-1.925325	0.002957
8	7	0	-1.850172	2.087809	-0.058873
9	7	0	1.548308	-1.077723	0.003499
10	7	0	1.112153	1.079601	-0.001346
11	1	0	-1.194225	2.804570	0.217674
12	1	0	-2.816183	2.264341	0.179656
13	1	0	2.071965	-1.942278	0.004468
14	1	0	-2.772660	-2.195084	0.004264
15	8	0	3.353455	0.413299	-0.001400
16	1	0	1.256213	2.079290	-0.006967

Result for 8OA_6imino8enol(a).log

```
SCF Done:  E(RB3LYP) =  -542.720474640     A.U. after    1 cycles
```

```
Zero-point correction=                           0.115910 (Hartree/Particle)
```

```
Thermal correction to Energy=                    0.124144
Thermal correction to Enthalpy=                  0.125088
Thermal correction to Gibbs Free Energy=         0.082861
Sum of electronic and zero-point Energies=           -542.604565
Sum of electronic and thermal Energies=              -542.596330
Sum of electronic and thermal Enthalpies=            -542.595386
Sum of electronic and thermal Free Energies=         -542.637614

                    E (Thermal)             CV                S
                     KCal/Mol        Cal/Mol-Kelvin    Cal/Mol-Kelvin
Total                  77.902             32.815            88.876

---------------------------------------------------------------------
Center     Atomic      Atomic             Coordinates (Angstroms)
Number     Number       Type             X           Y           Z
---------------------------------------------------------------------
     1          6           0        0.119156   -0.904000    0.000238
     2          6           0       -0.062290    0.465326    0.000046
     3          6           0       -1.399878    1.001603   -0.000018
     4          6           0       -2.027877   -1.409408   -0.000104
     5          6           0        2.037252    0.157701   -0.000016
     6          7           0       -2.334303   -0.079457   -0.000402
     7          7           0       -0.823495   -1.885573    0.000152
     8          7           0       -1.716259    2.238504    0.000294
     9          7           0        1.488443   -1.094794    0.000030
    10          7           0        1.158901    1.118936   -0.000112
    11          1           0        1.977958   -1.975240    0.000948
    12          1           0       -2.874753   -2.086060   -0.000219
    13          8           0        3.370434    0.289052   -0.000198
    14          1           0       -3.311341    0.175645   -0.000041
    15          1           0       -2.725313    2.384214    0.000198
    16          1           0        3.558784    1.238382    0.000077
---------------------------------------------------------------------
```

Result for 8OA_6imino8enol(b).log

```
SCF Done:  E(RB3LYP) =  -542.730078711     A.U. after    1 cycles


Zero-point correction=                           0.116537 (Hartree/Particle)
Thermal correction to Energy=                    0.124686
Thermal correction to Enthalpy=                  0.125630
Thermal correction to Gibbs Free Energy=         0.083558
Sum of electronic and zero-point Energies=           -542.613542
Sum of electronic and thermal Energies=              -542.605393
Sum of electronic and thermal Enthalpies=            -542.604449
Sum of electronic and thermal Free Energies=         -542.646521

                    E (Thermal)             CV                S
                     KCal/Mol        Cal/Mol-Kelvin    Cal/Mol-Kelvin
Total                  78.242             32.451            88.548

---------------------------------------------------------------------
Center     Atomic      Atomic             Coordinates (Angstroms)
```

Number	Number	Type	X	Y	Z
1	6	0	0.100406	-0.912775	-0.000019
2	6	0	-0.067011	0.455706	-0.000061
3	6	0	-1.396528	1.005727	-0.000064
4	6	0	-2.056806	-1.377438	-0.000004
5	6	0	2.030318	0.134257	0.000050
6	7	0	-2.343171	-0.049446	-0.000117
7	7	0	-0.857628	-1.878043	0.000021
8	7	0	-1.831803	2.206418	0.000001
9	7	0	1.469637	-1.113033	-0.000068
10	7	0	1.158950	1.103760	-0.000049
11	1	0	1.954678	-1.996194	0.000679
12	1	0	-2.912718	-2.042037	0.000024
13	8	0	3.364600	0.251023	0.000136
14	1	0	-3.308324	0.252316	-0.000030
15	1	0	-1.052331	2.860333	0.000013
16	1	0	3.567722	1.196948	0.000291

Result for 8OA_6imino8enol(c).log

```
SCF Done:  E(RB3LYP) =  -542.719664754     A.U. after    1 cycles

Zero-point correction=                           0.115657 (Hartree/Particle)
Thermal correction to Energy=                    0.124250
Thermal correction to Enthalpy=                  0.125194
Thermal correction to Gibbs Free Energy=         0.081927
Sum of electronic and zero-point Energies=           -542.604008
Sum of electronic and thermal Energies=              -542.595415
Sum of electronic and thermal Enthalpies=            -542.594471
Sum of electronic and thermal Free Energies=         -542.637738

                    E (Thermal)            CV                S
                     KCal/Mol        Cal/Mol-Kelvin    Cal/Mol-Kelvin
Total                   77.968            33.010             91.063
```

Center Number	Atomic Number	Atomic Type	Coordinates (Angstroms) X	Y	Z
1	6	0	-0.112038	-0.901839	0.001006
2	6	0	0.076756	0.463174	0.002370
3	6	0	1.416512	0.991456	-0.001878
4	6	0	2.036765	-1.402796	-0.000020
5	6	0	-2.028290	0.191325	0.000778
6	7	0	2.345529	-0.080146	-0.001284
7	7	0	0.829586	-1.884423	-0.001092
8	7	0	1.870961	2.183773	-0.004361
9	7	0	-1.482996	-1.077122	-0.004111
10	7	0	-1.133258	1.133697	-0.005191
11	1	0	-1.962478	-1.956342	0.109663
12	1	0	2.881539	-2.081614	0.001833

13	8	0	-3.356168	0.406869	0.056961
14	1	0	-3.834271	-0.250267	-0.462831
15	1	0	1.101792	2.850053	-0.004172
16	1	0	3.315767	0.204840	-0.001440

Result for 8OA_6imino8enol(d).log

```
SCF Done:  E(RB3LYP) =  -542.699632202     A.U. after    1 cycles
```

```
Zero-point correction=                           0.114694 (Hartree/Particle)
Thermal correction to Energy=                    0.123640
Thermal correction to Enthalpy=                  0.124584
Thermal correction to Gibbs Free Energy=         0.080740
Sum of electronic and zero-point Energies=          -542.584938
Sum of electronic and thermal Energies=             -542.575992
Sum of electronic and thermal Enthalpies=           -542.575048
Sum of electronic and thermal Free Energies=        -542.618892
```

	E (Thermal) KCal/Mol	CV Cal/Mol-Kelvin	S Cal/Mol-Kelvin
Total	77.585	34.012	92.278

Center Number	Atomic Number	Atomic Type	Coordinates (Angstroms) X	Y	Z
1	6	0	0.116566	-0.877077	0.002959
2	6	0	-0.075587	0.476645	0.008581
3	6	0	-1.425650	1.015255	0.000644
4	6	0	-2.180403	-1.230768	0.003629
5	6	0	2.034680	0.163543	-0.000149
6	7	0	-2.447710	0.019429	0.005103
7	7	0	-0.915213	-1.783590	-0.025943
8	7	0	-1.688121	2.263738	-0.007561
9	7	0	1.477622	-1.097584	-0.005093
10	7	0	1.162272	1.119273	0.007322
11	1	0	1.980567	-1.969425	-0.023651
12	1	0	-2.986457	-1.959069	0.016394
13	8	0	3.371785	0.270423	0.000169
14	1	0	-0.784270	-2.773855	0.101232
15	1	0	3.577478	1.216096	0.005698
16	1	0	-2.701188	2.388417	-0.011799

Result for 8OA_6imino8enol(e).log

```
SCF Done:  E(RB3LYP) =  -542.698961486     A.U. after    1 cycles
```

```
Zero-point correction=                           0.114845 (Hartree/Particle)
Thermal correction to Energy=                    0.123721
Thermal correction to Enthalpy=                  0.124665
Thermal correction to Gibbs Free Energy=         0.081013
Sum of electronic and zero-point Energies=          -542.584116
Sum of electronic and thermal Energies=             -542.575241
Sum of electronic and thermal Enthalpies=           -542.574296
Sum of electronic and thermal Free Energies=        -542.617948

                    E (Thermal)             CV                S
                     KCal/Mol        Cal/Mol-Kelvin    Cal/Mol-Kelvin
Total                   77.636             33.905             91.873
```

Center Number	Atomic Number	Atomic Type	Coordinates (Angstroms) X	Y	Z
1	6	0	0.087017	-0.892583	0.004441
2	6	0	-0.086928	0.460845	0.009752
3	6	0	-1.423331	1.035238	0.000777
4	6	0	-2.218791	-1.185660	0.003761
5	6	0	2.020596	0.125195	-0.000490
6	7	0	-2.462378	0.067091	0.005659
7	7	0	-0.962906	-1.774991	-0.030602
8	7	0	-1.734784	2.273840	-0.008938
9	7	0	1.446936	-1.127581	-0.005496
10	7	0	1.158110	1.092231	0.007460
11	1	0	1.941351	-2.004553	-0.024182
12	1	0	-3.039232	-1.897842	0.018355
13	8	0	3.358625	0.213282	0.000193
14	1	0	-0.857915	-2.765110	0.120939
15	1	0	-0.881681	2.834391	-0.008775
16	1	0	3.582257	1.154525	0.006106

Result for 8OA_6imino8keto(a).log

```
SCF Done:  E(RB3LYP) =  -542.744705724     A.U. after    1 cycles

Zero-point correction=                           0.116038 (Hartree/Particle)
Thermal correction to Energy=                    0.124333
Thermal correction to Enthalpy=                  0.125277
Thermal correction to Gibbs Free Energy=         0.082846
Sum of electronic and zero-point Energies=          -542.628668
Sum of electronic and thermal Energies=             -542.620372
Sum of electronic and thermal Enthalpies=           -542.619428
Sum of electronic and thermal Free Energies=        -542.661859

                    E (Thermal)             CV                S
                     KCal/Mol        Cal/Mol-Kelvin    Cal/Mol-Kelvin
Total                   78.020             32.836             89.304

---------------------------------------------------------------------
```

```
Center     Atomic      Atomic             Coordinates (Angstroms)
Number     Number       Type             X           Y           Z
---------------------------------------------------------------------
     1          6           0       -0.156562   -0.913295   -0.000129
     2          6           0        0.074119    0.436222   -0.000291
     3          6           0        1.394643    0.970693   -0.000017
     4          6           0        2.001601   -1.428031    0.000178
     5          6           0       -2.185538    0.142673   -0.000038
     6          7           0        2.326650   -0.101464    0.000018
     7          7           0        0.790486   -1.889883    0.000088
     8          7           0        1.667151    2.223545    0.000146
     9          7           0       -1.522556   -1.094540   -0.000174
    10          7           0       -1.151937    1.074470   -0.000279
    11          1           0        2.666228    2.418552    0.000388
    12          1           0       -2.009212   -1.975706    0.000702
    13          1           0        2.839284   -2.115440    0.000340
    14          8           0       -3.380313    0.340213    0.000204
    15          1           0       -1.297754    2.070664   -0.000219
    16          1           0        3.305810    0.145752    0.000340
---------------------------------------------------------------------
```

Result for 8OA_6imino8keto(a)-N9H.log

```
SCF Done:  E(RB3LYP) =  -542.190282748     A.U. after    2 cycles

Zero-point correction=                           0.101635 (Hartree/Particle)
Thermal correction to Energy=                    0.109948
Thermal correction to Enthalpy=                  0.110893
Thermal correction to Gibbs Free Energy=         0.068312
Sum of electronic and zero-point Energies=           -542.088647
Sum of electronic and thermal Energies=              -542.080334
Sum of electronic and thermal Enthalpies=            -542.079390
Sum of electronic and thermal Free Energies=         -542.121970

                    E (Thermal)             CV                S
                     KCal/Mol        Cal/Mol-Kelvin    Cal/Mol-Kelvin
Total                  68.994             32.214             89.617

---------------------------------------------------------------------
Center     Atomic      Atomic             Coordinates (Angstroms)
Number     Number       Type             X           Y           Z
---------------------------------------------------------------------
     1          6           0       -0.238517   -0.938073   -0.000123
     2          6           0        0.054175    0.435616   -0.000235
     3          6           0        1.356433    0.959614    0.000078
     4          6           0        1.957682   -1.436927    0.000171
     5          6           0       -2.182172    0.052052   -0.000791
     6          7           0        2.292340   -0.107112    0.000105
     7          7           0        0.753821   -1.901424    0.000105
     8          7           0        1.688565    2.225387    0.000238
     9          7           0       -1.560712   -1.179437    0.000103
    10          7           0       -1.175460    1.051395   -0.000177
    11          1           0        2.702243    2.338857    0.000402
```

```
    12          1           0        2.803734   -2.119278    0.000308
    13          8           0       -3.387815    0.317707    0.000180
    14          1           0       -1.348083    2.041441    0.000187
    15          1           0        3.269145    0.141969    0.000448
 ---------------------------------------------------------------------
```

Result for 8OA_6imino8keto(a)-N9H_water.log

```
 SCF Done:  E(RB3LYP) =  -542.308371785     A.U. after    1 cycles

 Zero-point correction=                           0.103165 (Hartree/Particle)
 Thermal correction to Energy=                    0.111104
 Thermal correction to Enthalpy=                  0.112048
 Thermal correction to Gibbs Free Energy=         0.070214
 Sum of electronic and zero-point Energies=           -542.205207
 Sum of electronic and thermal Energies=              -542.197268
 Sum of electronic and thermal Enthalpies=            -542.196324
 Sum of electronic and thermal Free Energies=         -542.238158

                     E (Thermal)             CV                S
                      KCal/Mol        Cal/Mol-Kelvin    Cal/Mol-Kelvin
 Total                   69.719             31.038             88.049

 ---------------------------------------------------------------------
 Center     Atomic      Atomic             Coordinates (Angstroms)
 Number     Number       Type             X           Y           Z
 ---------------------------------------------------------------------
      1          6           0       -0.222653   -0.928529   -0.000045
      2          6           0        0.049184    0.431840   -0.000042
      3          6           0        1.359808    0.953286    0.000123
      4          6           0        1.966623   -1.422952    0.000142
      5          6           0       -2.160707    0.054865   -0.000164
      6          7           0        2.289949   -0.103212    0.000175
      7          7           0        0.754661   -1.895710    0.000030
      8          7           0        1.684219    2.214213    0.000212
      9          7           0       -1.564248   -1.170370   -0.000151
     10          7           0       -1.188250    1.048998   -0.000104
     11          1           0        2.697987    2.320721    0.000339
     12          1           0        2.812232   -2.098753    0.000202
     13          8           0       -3.399529    0.301020   -0.000244
     14          1           0       -1.374680    2.040084   -0.000104
     15          1           0        3.272851    0.141294    0.000298
 ---------------------------------------------------------------------
```

Result for 8OA_6imino8keto(a)_water.log

```
 SCF Done:  E(RB3LYP) =  -542.781051872     A.U. after    8 cycles

 Zero-point correction=                           0.116132 (Hartree/Particle)
```

```
Thermal correction to Energy=                   0.124337
Thermal correction to Enthalpy=                 0.125281
Thermal correction to Gibbs Free Energy=        0.083036
Sum of electronic and zero-point Energies=          -542.664920
Sum of electronic and thermal Energies=             -542.656715
Sum of electronic and thermal Enthalpies=           -542.655770
Sum of electronic and thermal Free Energies=        -542.698016

                    E (Thermal)             CV                S
                     KCal/Mol        Cal/Mol-Kelvin    Cal/Mol-Kelvin
Total                   78.023             32.447            88.912
```

Center Number	Atomic Number	Atomic Type	Coordinates (Angstroms) X	Y	Z
1	6	0	-0.163303	-0.908490	0.000115
2	6	0	0.073317	0.440103	-0.000053
3	6	0	1.396501	0.958413	-0.000226
4	6	0	1.994531	-1.422993	-0.000008
5	6	0	-2.167999	0.145537	0.000167
6	7	0	2.318011	-0.107524	-0.000180
7	7	0	0.776160	-1.890608	0.000144
8	7	0	1.705939	2.210624	-0.000399
9	7	0	-1.530587	-1.084028	0.000247
10	7	0	-1.158944	1.078057	-0.000021
11	1	0	2.717645	2.336151	-0.000503
12	1	0	-2.022275	-1.967058	0.000397
13	1	0	2.833693	-2.105469	-0.000001
14	8	0	-3.385408	0.350883	0.000251
15	1	0	-1.321109	2.074666	-0.000119
16	1	0	3.302971	0.133578	-0.000286

Result for 8OA_6imino8keto(b).log

```
SCF Done:  E(RB3LYP) =  -542.746284163     A.U. after    1 cycles


Zero-point correction=                          0.116042 (Hartree/Particle)
Thermal correction to Energy=                   0.124461
Thermal correction to Enthalpy=                 0.125405
Thermal correction to Gibbs Free Energy=        0.082601
Sum of electronic and zero-point Energies=          -542.630242
Sum of electronic and thermal Energies=             -542.621823
Sum of electronic and thermal Enthalpies=           -542.620879
Sum of electronic and thermal Free Energies=        -542.663683

                    E (Thermal)             CV                S
                     KCal/Mol        Cal/Mol-Kelvin    Cal/Mol-Kelvin
Total                   78.101             32.849            90.090

---------------------------------------------------------------------
Center     Atomic      Atomic             Coordinates (Angstroms)
```

Number	Number	Type	X	Y	Z
1	6	0	-0.149703	-0.905736	0.000004
2	6	0	0.076781	0.447159	0.000014
3	6	0	1.405089	0.971010	-0.000078
4	6	0	2.006927	-1.418532	0.000019
5	6	0	-2.188238	0.128981	0.000172
6	7	0	2.326820	-0.101059	-0.000062
7	7	0	0.792997	-1.883598	0.000009
8	7	0	1.876601	2.164307	-0.000126
9	7	0	-1.513767	-1.097830	-0.000273
10	7	0	-1.164300	1.073227	-0.000047
11	1	0	-1.989862	-1.985066	0.000696
12	1	0	2.843433	-2.107039	0.000052
13	8	0	-3.383882	0.320460	0.000271
14	1	0	-1.354153	2.060960	-0.000016
15	1	0	3.298205	0.181012	-0.000042
16	1	0	1.139841	2.863829	-0.000152

Result for 8OA_6imino8keto(b)-N9H.log

```
SCF Done:  E(RB3LYP) =  -542.195853998     A.U. after   10 cycles

Zero-point correction=                           0.102315 (Hartree/Particle)
Thermal correction to Energy=                    0.110478
Thermal correction to Enthalpy=                  0.111422
Thermal correction to Gibbs Free Energy=         0.069112
Sum of electronic and zero-point Energies=           -542.093539
Sum of electronic and thermal Energies=              -542.085376
Sum of electronic and thermal Enthalpies=            -542.084432
Sum of electronic and thermal Free Energies=         -542.126742

                    E (Thermal)             CV                S
                     KCal/Mol        Cal/Mol-Kelvin    Cal/Mol-Kelvin
Total                  69.326             31.702             89.049
```

Center Number	Atomic Number	Atomic Type	Coordinates (Angstroms) X	Y	Z
1	6	0	0.224492	-0.937214	-0.004288
2	6	0	-0.058085	0.438555	0.006791
3	6	0	-1.363094	0.960907	0.001832
4	6	0	-1.974894	-1.417591	0.007582
5	6	0	2.179200	0.028829	-0.005997
6	7	0	-2.298619	-0.093127	0.018197
7	7	0	-0.772971	-1.894115	-0.005229
8	7	0	-1.849726	2.176116	-0.009737
9	7	0	1.545336	-1.192045	-0.018884
10	7	0	1.183558	1.046140	-0.039971
11	1	0	-2.823786	-2.095814	0.012618
12	8	0	3.385518	0.287050	0.026534

```
    13          1           0        1.395024    2.011800    0.143912
    14          1           0       -3.265438    0.195921    0.015988
    15          1           0       -1.088705    2.850001   -0.030940
---------------------------------------------------------------------
```

Result for 8OA_6imino8keto(b)-N9H_water.log

```
SCF Done:  E(RB3LYP) =  -542.309905964     A.U. after    1 cycles


Zero-point correction=                           0.103361 (Hartree/Particle)
Thermal correction to Energy=                    0.111271
Thermal correction to Enthalpy=                  0.112215
Thermal correction to Gibbs Free Energy=         0.070433
Sum of electronic and zero-point Energies=           -542.206545
Sum of electronic and thermal Energies=              -542.198635
Sum of electronic and thermal Enthalpies=            -542.197691
Sum of electronic and thermal Free Energies=         -542.239473

                     E (Thermal)             CV                S
                      KCal/Mol        Cal/Mol-Kelvin    Cal/Mol-Kelvin
Total                    69.824            30.917             87.937

---------------------------------------------------------------------
Center     Atomic      Atomic             Coordinates (Angstroms)
Number     Number       Type             X           Y           Z
---------------------------------------------------------------------
     1          6           0        0.201243   -0.933789    0.000023
     2          6           0       -0.054834    0.430895    0.000091
     3          6           0       -1.361511    0.964021    0.000004
     4          6           0       -1.993821   -1.399546   -0.000038
     5          6           0        2.151449    0.025452    0.000000
     6          7           0       -2.300121   -0.078621    0.000070
     7          7           0       -0.787203   -1.888745   -0.000022
     8          7           0       -1.795386    2.192679   -0.000063
     9          7           0        1.539037   -1.191968   -0.000016
    10          7           0        1.191985    1.032277    0.000140
    11          1           0       -2.847406   -2.065003   -0.000063
    12          8           0        3.392616    0.255639   -0.000067
    13          1           0        1.392863    2.021086   -0.000338
    14          1           0       -3.277383    0.186486   -0.000264
    15          1           0       -1.002347    2.830768   -0.000049
---------------------------------------------------------------------
```

Result for 8OA_6imino8keto(b)_water.log

```
SCF Done:  E(RB3LYP) =  -542.782122632     A.U. after    8 cycles


Zero-point correction=                           0.116372 (Hartree/Particle)
Thermal correction to Energy=                    0.124524
```

```
Thermal correction to Enthalpy=                  0.125468
Thermal correction to Gibbs Free Energy=         0.083337
Sum of electronic and zero-point Energies=           -542.665751
Sum of electronic and thermal Energies=              -542.657599
Sum of electronic and thermal Enthalpies=            -542.656654
Sum of electronic and thermal Free Energies=         -542.698786

                    E (Thermal)             CV                S
                     KCal/Mol        Cal/Mol-Kelvin    Cal/Mol-Kelvin
Total                   78.140             32.304            88.674

---------------------------------------------------------------------
Center     Atomic      Atomic             Coordinates (Angstroms)
Number     Number       Type             X           Y           Z
---------------------------------------------------------------------
     1          6           0       -0.144654   -0.910926    0.000161
     2          6           0        0.078345    0.441636   -0.000086
     3          6           0        1.399868    0.968632   -0.000248
     4          6           0        2.017322   -1.402951    0.000103
     5          6           0       -2.161490    0.119430    0.000113
     6          7           0        2.326271   -0.087444   -0.000136
     7          7           0        0.802803   -1.883899    0.000266
     8          7           0        1.832889    2.184648   -0.000461
     9          7           0       -1.508919   -1.102095    0.000281
    10          7           0       -1.164101    1.064025   -0.000120
    11          1           0       -1.989592   -1.991315    0.000483
    12          1           0        2.862963   -2.077145    0.000160
    13          8           0       -3.380890    0.310208    0.000164
    14          1           0       -1.341159    2.058671   -0.000280
    15          1           0        3.306393    0.171705   -0.000242
    16          1           0        1.049564    2.834845   -0.000507
---------------------------------------------------------------------
```

Result for 8OA_6imino8keto(c).log

```
SCF Done:  E(RB3LYP) =  -542.718708984     A.U. after    8 cycles


Zero-point correction=                           0.114260 (Hartree/Particle)
Thermal correction to Energy=                    0.123530
Thermal correction to Enthalpy=                  0.124474
Thermal correction to Gibbs Free Energy=         0.079511
Sum of electronic and zero-point Energies=           -542.604449
Sum of electronic and thermal Energies=              -542.595179
Sum of electronic and thermal Enthalpies=            -542.594235
Sum of electronic and thermal Free Energies=         -542.639198

                    E (Thermal)             CV                S
                     KCal/Mol        Cal/Mol-Kelvin    Cal/Mol-Kelvin
Total                   77.516             34.296            94.633

---------------------------------------------------------------------
Center     Atomic      Atomic             Coordinates (Angstroms)
Number     Number       Type             X           Y           Z
```

1	6	0	0.133990	-0.885601	-0.000000
2	6	0	-0.095876	0.451756	-0.000001
3	6	0	-1.433154	0.999323	-0.000000
4	6	0	-2.169794	-1.237224	-0.000000
5	6	0	2.176219	0.121328	0.000000
6	7	0	-2.444629	0.010441	-0.000001
7	7	0	-0.898672	-1.782546	0.000002
8	7	0	-1.783850	2.233290	0.000001
9	7	0	1.490747	-1.112397	0.000001
10	7	0	1.162944	1.062675	-0.000001
11	1	0	1.981226	-1.990759	0.000001
12	1	0	-2.971027	-1.970080	-0.000001
13	8	0	3.376174	0.283240	0.000000
14	1	0	1.353812	2.050158	-0.000003
15	1	0	-0.758224	-2.779473	-0.000004
16	1	0	-0.969282	2.846507	0.000002

Result for 8OA_6imino8keto(d).log

```
SCF Done:  E(RB3LYP) =  -542.728070856     A.U. after    8 cycles

Zero-point correction=                           0.114872 (Hartree/Particle)
Thermal correction to Energy=                    0.123941
Thermal correction to Enthalpy=                  0.124885
Thermal correction to Gibbs Free Energy=         0.080501
Sum of electronic and zero-point Energies=           -542.613199
Sum of electronic and thermal Energies=              -542.604130
Sum of electronic and thermal Enthalpies=            -542.603186
Sum of electronic and thermal Free Energies=         -542.647570

                    E (Thermal)             CV                S
                     KCal/Mol        Cal/Mol-Kelvin    Cal/Mol-Kelvin
Total                   77.774             33.930             93.415
```

Center Number	Atomic Number	Atomic Type	Coordinates (Angstroms) X	Y	Z
1	6	0	0.150039	-0.889393	-0.000000
2	6	0	-0.086040	0.444722	-0.000000
3	6	0	-1.417150	0.986256	-0.000000
4	6	0	-2.156205	-1.249614	0.000000
5	6	0	2.179906	0.147688	0.000000
6	7	0	-2.436985	0.001250	0.000000
7	7	0	-0.886485	-1.787687	-0.000001
8	7	0	-1.629904	2.249166	-0.000000
9	7	0	1.509636	-1.100094	0.000002
10	7	0	1.154319	1.073374	0.000001
11	1	0	2.013145	-1.970710	-0.000006
12	8	0	3.378738	0.318422	-0.000001
13	1	0	1.294220	2.070542	-0.000000

```
    14          1           0        -2.632890    2.431750   -0.000000
    15          1           0        -0.746284   -2.784619    0.000003
    16          1           0        -2.955470   -1.984359    0.000000
 ---------------------------------------------------------------------
```

Result for 8OG.log

```
 SCF Done:  E(RB3LYP) =  -618.037321330     A.U. after    8 cycles


 Zero-point correction=                           0.120729 (Hartree/Particle)
 Thermal correction to Energy=                    0.130321
 Thermal correction to Enthalpy=                  0.131265
 Thermal correction to Gibbs Free Energy=         0.086039
 Sum of electronic and zero-point Energies=           -617.916592
 Sum of electronic and thermal Energies=              -617.907000
 Sum of electronic and thermal Enthalpies=            -617.906056
 Sum of electronic and thermal Free Energies=         -617.951282

                     E (Thermal)             CV                S
                      KCal/Mol        Cal/Mol-Kelvin    Cal/Mol-Kelvin
 Total                   81.778             37.498             95.187

 ---------------------------------------------------------------------
 Center     Atomic      Atomic             Coordinates (Angstroms)
 Number     Number       Type             X           Y           Z
 ---------------------------------------------------------------------
      1          6           0         0.396511    0.606483    0.006789
      2          6           0         0.245057   -0.762456    0.000311
      3          6           0        -1.982536   -0.688346   -0.002871
      4          6           0        -0.731676    1.457958    0.002511
      5          8           0        -0.793871    2.678242   -0.002534
      6          7           0         1.503964   -1.308532   -0.001966
      7          7           0        -0.916165   -1.449889    0.007978
      8          7           0        -1.927121    0.673812   -0.002967
      9          1           0        -2.775920    1.219100   -0.073318
     10          7           0         1.762957    0.877319    0.006628
     11          7           0        -3.225728   -1.267871   -0.068994
     12          1           0        -3.209559   -2.262723    0.102248
     13          1           0        -3.991031   -0.784115    0.376568
     14          6           0         2.489285   -0.300805    0.000585
     15          1           0         1.728714   -2.289941   -0.008733
     16          8           0         3.690450   -0.455931   -0.001531
     17          1           0         2.189966    1.788324    0.007047
 ---------------------------------------------------------------------
```

Result for 8OG-N9H.log

```
 SCF Done:  E(RB3LYP) =  -617.485990870     A.U. after    1 cycles
```

```
Zero-point correction=                           0.107161 (Hartree/Particle)
Thermal correction to Energy=                    0.116651
Thermal correction to Enthalpy=                  0.117596
Thermal correction to Gibbs Free Energy=         0.072096
Sum of electronic and zero-point Energies=           -617.378830
Sum of electronic and thermal Energies=              -617.369339
Sum of electronic and thermal Enthalpies=            -617.368395
Sum of electronic and thermal Free Energies=         -617.413895

                    E (Thermal)             CV                S
                     KCal/Mol        Cal/Mol-Kelvin    Cal/Mol-Kelvin
Total                  73.200             36.029             95.762
```

Center Number	Atomic Number	Atomic Type	X	Y	Z
1	6	0	-0.418214	0.597889	-0.012686
2	6	0	-0.313907	-0.809468	0.002309
3	6	0	1.940885	-0.687357	0.011077
4	6	0	0.686319	1.446924	-0.003260
5	8	0	0.773493	2.688390	0.008310
6	7	0	-1.509036	-1.409865	0.006214
7	7	0	0.902803	-1.460468	-0.004769
8	7	0	1.895140	0.674441	0.002908
9	7	0	-1.781520	0.838934	-0.022108
10	7	0	3.231222	-1.250301	0.079759
11	6	0	-2.457492	-0.399142	-0.001889
12	8	0	-3.686898	-0.496567	0.004717
13	1	0	2.737396	1.212293	0.143223
14	1	0	3.862568	-0.870754	-0.616946
15	1	0	3.147181	-2.253027	-0.037040
16	1	0	-2.235705	1.734638	-0.000788

(Coordinates (Angstroms))

Result for 8OG-N9H_water.log

```
SCF Done:  E(RB3LYP) =  -617.607008680     A.U. after    1 cycles
```

```
Zero-point correction=                           0.107061 (Hartree/Particle)
Thermal correction to Energy=                    0.116376
Thermal correction to Enthalpy=                  0.117320
Thermal correction to Gibbs Free Energy=         0.072472
Sum of electronic and zero-point Energies=           -617.499948
Sum of electronic and thermal Energies=              -617.490633
Sum of electronic and thermal Enthalpies=            -617.489689
Sum of electronic and thermal Free Energies=         -617.534536

                    E (Thermal)             CV                S
                     KCal/Mol        Cal/Mol-Kelvin    Cal/Mol-Kelvin
Total                  73.027             35.913             94.389

---------------------------------------------------------------------
```

Center Number	Atomic Number	Atomic Type	Coordinates (Angstroms) X	Y	Z
1	6	0	-0.417573	0.590749	-0.003712
2	6	0	-0.301848	-0.804124	-0.000088
3	6	0	1.952022	-0.694753	-0.003450
4	6	0	0.699609	1.418960	0.000247
5	8	0	0.761759	2.675091	0.003954
6	7	0	-1.519843	-1.398600	0.004191
7	7	0	0.888476	-1.465921	0.003055
8	7	0	1.891043	0.668778	0.001422
9	7	0	-1.784691	0.841494	-0.000463
10	7	0	3.200709	-1.248906	-0.075124
11	6	0	-2.436541	-0.380077	0.001654
12	8	0	-3.689866	-0.499519	0.001405
13	1	0	2.753600	1.201275	-0.006683
14	1	0	3.967032	-0.688927	0.275371
15	1	0	3.240769	-2.220188	0.201595
16	1	0	-2.240419	1.740812	-0.012625

Result for 8OG_water.log

```
SCF Done:  E(RB3LYP) =  -618.078682444     A.U. after    1 cycles

Zero-point correction=                           0.119986 (Hartree/Particle)
Thermal correction to Energy=                    0.129527
Thermal correction to Enthalpy=                  0.130471
Thermal correction to Gibbs Free Energy=         0.085420
Sum of electronic and zero-point Energies=           -617.958696
Sum of electronic and thermal Energies=              -617.949156
Sum of electronic and thermal Enthalpies=            -617.948212
Sum of electronic and thermal Free Energies=         -617.993263
```

	E (Thermal) KCal/Mol	CV Cal/Mol-Kelvin	S Cal/Mol-Kelvin
Total	81.279	37.444	94.818

Center Number	Atomic Number	Atomic Type	Coordinates (Angstroms) X	Y	Z
1	6	0	0.392103	0.605208	-0.003731
2	6	0	0.247360	-0.768257	-0.001058
3	6	0	-1.982448	-0.694349	-0.002220
4	6	0	-0.741276	1.432028	0.000304
5	8	0	-0.797728	2.674871	0.003214
6	7	0	1.511389	-1.303829	0.002366
7	7	0	-0.902181	-1.458929	0.001807
8	7	0	-1.921955	0.669288	0.001854
9	1	0	-2.788690	1.197567	0.000653
10	7	0	1.760654	0.879295	-0.002504
11	7	0	-3.208468	-1.259693	-0.058220

12	1	0	-3.256960	-2.243721	0.163491
13	1	0	-4.009848	-0.702583	0.204627
14	6	0	2.466774	-0.291662	0.001345
15	1	0	1.745668	-2.287170	0.003812
16	8	0	3.691053	-0.441971	0.002667
17	1	0	2.192086	1.791973	-0.004596

Result for A.log

```
SCF Done:  E(RB3LYP) =  -467.490304042     A.U. after    1 cycles

Zero-point correction=                           0.111476 (Hartree/Particle)
Thermal correction to Energy=                    0.118800
Thermal correction to Enthalpy=                  0.119744
Thermal correction to Gibbs Free Energy=         0.079716
Sum of electronic and zero-point Energies=           -467.378828
Sum of electronic and thermal Energies=              -467.371504
Sum of electronic and thermal Enthalpies=            -467.370560
Sum of electronic and thermal Free Energies=         -467.410588

                    E (Thermal)             CV                S
                     KCal/Mol        Cal/Mol-Kelvin    Cal/Mol-Kelvin
Total                   74.548             28.941             84.245
```

Center Number	Atomic Number	Atomic Type	Coordinates (Angstroms) X	Y	Z
1	6	0	-0.707174	0.773711	-0.003240
2	6	0	-0.176563	-0.516115	-0.004640
3	6	0	1.224947	-0.609479	-0.004547
4	6	0	1.297406	1.694807	0.003778
5	6	0	-2.289062	-0.772537	0.003756
6	7	0	1.943725	0.521289	0.004533
7	7	0	-0.016996	1.915418	-0.001002
8	7	0	1.871257	-1.798860	-0.037717
9	7	0	-2.070882	0.589448	0.000685
10	7	0	-1.182721	-1.468507	0.000902
11	1	0	1.347442	-2.643963	0.117396
12	1	0	2.865493	-1.800060	0.119949
13	1	0	-2.764709	1.319847	0.002291
14	1	0	1.929665	2.576006	0.009565
15	1	0	-3.285891	-1.185663	0.008346

Result for A-N9H.log

```
SCF Done:  E(RB3LYP) =  -466.940812558     A.U. after    1 cycles
```

```
Zero-point correction=                           0.098707 (Hartree/Particle)
Thermal correction to Energy=                    0.105478
Thermal correction to Enthalpy=                  0.106422
Thermal correction to Gibbs Free Energy=         0.067357
Sum of electronic and zero-point Energies=           -466.842105
Sum of electronic and thermal Energies=              -466.835334
Sum of electronic and thermal Enthalpies=            -466.834390
Sum of electronic and thermal Free Energies=         -466.873456

                    E (Thermal)             CV                S
                     KCal/Mol        Cal/Mol-Kelvin    Cal/Mol-Kelvin
Total                   66.189            26.814            82.220

---------------------------------------------------------------------
Center     Atomic      Atomic             Coordinates (Angstroms)
Number     Number       Type             X           Y           Z
---------------------------------------------------------------------
     1          6           0       -0.778471    0.799174   -0.007772
     2          6           0       -0.216143   -0.505875   -0.022133
     3          6           0        1.171523   -0.610153   -0.020620
     4          6           0        1.281852    1.681247    0.008105
     5          6           0       -2.313413   -0.655244    0.012677
     6          7           0        1.926339    0.493865    0.002723
     7          7           0       -0.019986    1.922045    0.001724
     8          7           0        1.812989   -1.846405   -0.074150
     9          7           0       -2.134130    0.687280    0.010149
    10          7           0       -1.221903   -1.441253   -0.006808
    11          1           0        1.227226   -2.604247    0.249938
    12          1           0        2.734112   -1.829147    0.341266
    13          1           0        1.933034    2.552724    0.023489
    14          1           0       -3.309608   -1.082961    0.028307
---------------------------------------------------------------------
```

Result for A-N9H_water.log

```
SCF Done:  E(RB3LYP) =  -467.042856407     A.U. after    1 cycles


Zero-point correction=                           0.099044 (Hartree/Particle)
Thermal correction to Energy=                    0.105868
Thermal correction to Enthalpy=                  0.106812
Thermal correction to Gibbs Free Energy=         0.067662
Sum of electronic and zero-point Energies=           -466.943812
Sum of electronic and thermal Energies=              -466.936989
Sum of electronic and thermal Enthalpies=            -466.936045
Sum of electronic and thermal Free Energies=         -466.975194

                    E (Thermal)             CV                S
                     KCal/Mol        Cal/Mol-Kelvin    Cal/Mol-Kelvin
Total                   66.433            26.967            82.398

---------------------------------------------------------------------
Center     Atomic      Atomic             Coordinates (Angstroms)
```

```
 Number     Number       Type             X           Y           Z
 ---------------------------------------------------------------------
      1          6           0       -0.770811    0.786476    0.001061
      2          6           0       -0.216074   -0.508582   -0.005307
      3          6           0        1.176702   -0.621685   -0.007628
      4          6           0        1.280148    1.684287    0.002433
      5          6           0       -2.325344   -0.655471    0.001175
      6          7           0        1.920355    0.499828   -0.000929
      7          7           0       -0.024937    1.919353    0.003680
      8          7           0        1.820752   -1.828948   -0.064870
      9          7           0       -2.130294    0.686675    0.005823
     10          7           0       -1.235873   -1.438232   -0.004222
     11          1           0        1.289409   -2.634276    0.233820
     12          1           0        2.788156   -1.826135    0.228056
     13          1           0        1.926647    2.555406    0.007654
     14          1           0       -3.321953   -1.075879    0.003693
 ---------------------------------------------------------------------
```

Result for A_water.log

```
 SCF Done:  E(RB3LYP) =  -467.517394382     A.U. after    1 cycles
```

```
 Zero-point correction=                           0.111521 (Hartree/Particle)
 Thermal correction to Energy=                    0.118806
 Thermal correction to Enthalpy=                  0.119750
 Thermal correction to Gibbs Free Energy=         0.079796
 Sum of electronic and zero-point Energies=           -467.405873
 Sum of electronic and thermal Energies=              -467.398589
 Sum of electronic and thermal Enthalpies=            -467.397645
 Sum of electronic and thermal Free Energies=         -467.437599

                     E (Thermal)             CV                S
                      KCal/Mol        Cal/Mol-Kelvin    Cal/Mol-Kelvin
 Total                  74.552             28.816            84.091

 ---------------------------------------------------------------------
 Center     Atomic      Atomic             Coordinates (Angstroms)
 Number     Number       Type             X           Y           Z
 ---------------------------------------------------------------------
      1          6           0       -0.714139    0.765607   -0.000064
      2          6           0       -0.172393   -0.517947   -0.003083
      3          6           0        1.228844   -0.611368   -0.003931
      4          6           0        1.290439    1.700329    0.001916
      5          6           0       -2.289998   -0.771743    0.001448
      6          7           0        1.943945    0.531210    0.000872
      7          7           0       -0.024545    1.915125    0.001495
      8          7           0        1.882883   -1.790753   -0.042313
      9          7           0       -2.072761    0.580316    0.002197
     10          7           0       -1.179633   -1.473201   -0.002174
     11          1           0        1.372106   -2.639836    0.145636
     12          1           0        2.875382   -1.792198    0.141850
     13          1           0       -2.782815    1.299973    0.005871
     14          1           0        1.917351    2.584079    0.005145
```

```
    15          1           0       -3.287776   -1.180182    0.003239
 ---------------------------------------------------------------------
```

Result for eC.log

```
 SCF Done:  E(RB3LYP) =  -471.312416221     A.U. after    1 cycles

 Zero-point correction=                           0.110947 (Hartree/Particle)
 Thermal correction to Energy=                    0.117852
 Thermal correction to Enthalpy=                  0.118796
 Thermal correction to Gibbs Free Energy=         0.079414
 Sum of electronic and zero-point Energies=           -471.201469
 Sum of electronic and thermal Energies=              -471.194565
 Sum of electronic and thermal Enthalpies=            -471.193620
 Sum of electronic and thermal Free Energies=         -471.233003

                     E (Thermal)             CV                S
                      KCal/Mol        Cal/Mol-Kelvin    Cal/Mol-Kelvin
 Total                   73.953             27.660             82.887

 ---------------------------------------------------------------------
 Center     Atomic      Atomic             Coordinates (Angstroms)
 Number     Number       Type             X           Y           Z
 ---------------------------------------------------------------------
      1          6           0       -0.363539   -1.868037    0.000113
      2          6           0       -1.642733   -1.447002   -0.000389
      3          6           0       -1.032664    0.934542    0.000026
      4          6           0        0.662559   -0.880296   -0.000377
      5          8           0       -1.334284    2.108090   -0.000064
      6          7           0       -1.950001   -0.097073    0.000231
      7          1           0       -2.912287    0.204868    0.001437
      8          7           0        0.281033    0.467077   -0.000110
      9          1           0       -2.490364   -2.115912   -0.000253
     10          1           0       -0.105124   -2.914821    0.000016
     11          7           0        1.971574   -0.991654    0.000439
     12          6           0        2.455455    0.298767   -0.000008
     13          1           0        3.515492    0.495007   -0.000227
     14          6           0        1.442947    1.216516   -0.000164
     15          1           0        1.416158    2.290743    0.000414
 ---------------------------------------------------------------------
```

Result for eC-N1H.log

```
 SCF Done:  E(RB3LYP) =  -470.766767909     A.U. after    1 cycles

 Zero-point correction=                           0.096969 (Hartree/Particle)
 Thermal correction to Energy=                    0.103624
 Thermal correction to Enthalpy=                  0.104568
 Thermal correction to Gibbs Free Energy=         0.065631
```

```
Sum of electronic and zero-point Energies=          -470.669799
Sum of electronic and thermal Energies=             -470.663144
Sum of electronic and thermal Enthalpies=           -470.662200
Sum of electronic and thermal Free Energies=        -470.701137

                    E (Thermal)           CV                S
                     KCal/Mol      Cal/Mol-Kelvin    Cal/Mol-Kelvin
Total                  65.025           26.471            81.949

---------------------------------------------------------------------
Center     Atomic      Atomic             Coordinates (Angstroms)
Number     Number       Type             X           Y           Z
---------------------------------------------------------------------
     1          6           0       -0.394382   -1.856179    0.000071
     2          6           0       -1.682297   -1.374636    0.000083
     3          6           0       -1.122851    0.888487   -0.000050
     4          6           0        0.635248   -0.888491    0.000044
     5          8           0       -1.322829    2.107195    0.000211
     6          7           0       -2.062158   -0.079461   -0.000390
     7          7           0        0.238081    0.440431    0.000109
     8          1           0       -2.505899   -2.087711    0.000365
     9          1           0       -0.163450   -2.911446    0.000250
    10          7           0        1.966242   -0.989951   -0.000086
    11          6           0        2.419323    0.309685   -0.000049
    12          1           0        3.477293    0.529678    0.000095
    13          6           0        1.386032    1.209792    0.000034
    14          1           0        1.333095    2.282834   -0.000634
---------------------------------------------------------------------
```

Result for eC-N1H_water.log

```
SCF Done:  E(RB3LYP) =  -470.859981459     A.U. after    1 cycles

Zero-point correction=                           0.097530 (Hartree/Particle)
Thermal correction to Energy=                    0.104138
Thermal correction to Enthalpy=                  0.105083
Thermal correction to Gibbs Free Energy=         0.066202
Sum of electronic and zero-point Energies=          -470.762452
Sum of electronic and thermal Energies=             -470.755843
Sum of electronic and thermal Enthalpies=           -470.754899
Sum of electronic and thermal Free Energies=        -470.793780

                    E (Thermal)           CV                S
                     KCal/Mol      Cal/Mol-Kelvin    Cal/Mol-Kelvin
Total                  65.348           26.215            81.832

---------------------------------------------------------------------
Center     Atomic      Atomic             Coordinates (Angstroms)
Number     Number       Type             X           Y           Z
---------------------------------------------------------------------
     1          6           0       -0.417944   -1.849413   -0.000040
     2          6           0       -1.698051   -1.368377    0.000064
     3          6           0       -1.097116    0.881696    0.000033
```

```
     4          6           0        0.617463   -0.891920    0.000039
     5          8           0       -1.309186    2.121530   -0.000087
     6          7           0       -2.052746   -0.052953    0.000114
     7          7           0        0.241444    0.445318   -0.000090
     8          1           0       -2.528305   -2.066239   -0.000281
     9          1           0       -0.198086   -2.906949   -0.000290
    10          7           0        1.945159   -1.008965    0.000078
    11          6           0        2.421471    0.287153    0.000059
    12          1           0        3.480083    0.494156   -0.000310
    13          6           0        1.405702    1.199080   -0.000193
    14          1           0        1.393646    2.273679    0.001090
 ---------------------------------------------------------------------
```

Result for eC_water.log

```
 SCF Done:  E(RB3LYP) =  -471.332582049     A.U. after    1 cycles


 Zero-point correction=                           0.111018 (Hartree/Particle)
 Thermal correction to Energy=                    0.117844
 Thermal correction to Enthalpy=                  0.118788
 Thermal correction to Gibbs Free Energy=         0.079567
 Sum of electronic and zero-point Energies=          -471.221564
 Sum of electronic and thermal Energies=             -471.214738
 Sum of electronic and thermal Enthalpies=           -471.213794
 Sum of electronic and thermal Free Energies=        -471.253015

                     E (Thermal)             CV                S
                      KCal/Mol        Cal/Mol-Kelvin    Cal/Mol-Kelvin
 Total                   73.948             27.436             82.549

 ---------------------------------------------------------------------
 Center     Atomic      Atomic             Coordinates (Angstroms)
 Number     Number       Type             X           Y           Z
 ---------------------------------------------------------------------
     1          6           0       -0.378147   -1.863623    0.000059
     2          6           0       -1.654431   -1.428864   -0.000486
     3          6           0       -1.026496    0.923925    0.000065
     4          6           0        0.651433   -0.886993    0.000199
     5          8           0       -1.309619    2.118722   -0.000312
     6          7           0       -1.948989   -0.080371    0.000320
     7          1           0       -2.917811    0.213819    0.000927
     8          7           0        0.282326    0.460752    0.000158
     9          1           0       -2.511372   -2.084930   -0.000301
    10          1           0       -0.138340   -2.915416   -0.000240
    11          7           0        1.966217   -1.005048    0.000097
    12          6           0        2.456125    0.288750   -0.000124
    13          1           0        3.515747    0.485707   -0.000769
    14          6           0        1.449898    1.207093   -0.000091
    15          1           0        1.441560    2.281989    0.001122
 ---------------------------------------------------------------------
```

Result for G.log

```
 SCF Done:  E(RB3LYP) =  -542.755132069     A.U. after    1 cycles


 Zero-point correction=                           0.116258 (Hartree/Particle)
 Thermal correction to Energy=                    0.124528
 Thermal correction to Enthalpy=                  0.125472
 Thermal correction to Gibbs Free Energy=         0.083217
 Sum of electronic and zero-point Energies=           -542.638874
 Sum of electronic and thermal Energies=              -542.630604
 Sum of electronic and thermal Enthalpies=            -542.629660
 Sum of electronic and thermal Free Energies=         -542.671915

                     E (Thermal)             CV                S
                      KCal/Mol        Cal/Mol-Kelvin    Cal/Mol-Kelvin
 Total                  78.143             32.869             88.933

 ---------------------------------------------------------------------
 Center     Atomic      Atomic             Coordinates (Angstroms)
 Number     Number       Type             X           Y           Z
 ---------------------------------------------------------------------
      1          6           0       -0.851479    0.504982   -0.007486
      2          6           0       -0.529241   -0.848026    0.000484
      3          6           0        1.671060   -0.564760    0.003349
      4          6           0        0.213390    1.467128   -0.002908
      5          8           0        0.194484    2.679934    0.003344
      6          7           0       -1.730967   -1.501123    0.003510
      7          7           0        0.693478   -1.431591   -0.007493
      8          7           0        1.477022    0.787378    0.002787
      9          1           0        2.270251    1.410571    0.073574
     10          7           0       -2.221911    0.678957   -0.007493
     11          7           0        2.971659   -1.007027    0.069646
     12          1           0        3.060123   -1.997442   -0.105518
     13          1           0        3.674653   -0.445559   -0.387665
     14          6           0       -2.714992   -0.527886   -0.000944
     15          1           0       -3.763571   -0.780797    0.000629
     16          1           0       -1.854720   -2.501020    0.010550
 ---------------------------------------------------------------------
```

Result for G-N9H.log

```
 SCF Done:  E(RB3LYP) =  -542.206089679     A.U. after    8 cycles


 Zero-point correction=                           0.103134 (Hartree/Particle)
 Thermal correction to Energy=                    0.111103
 Thermal correction to Enthalpy=                  0.112047
 Thermal correction to Gibbs Free Energy=         0.070235
 Sum of electronic and zero-point Energies=           -542.102955
 Sum of electronic and thermal Energies=              -542.094987
 Sum of electronic and thermal Enthalpies=            -542.094042
```

```
Sum of electronic and thermal Free Energies=        -542.135855

                    E (Thermal)             CV                S
                     KCal/Mol        Cal/Mol-Kelvin    Cal/Mol-Kelvin
Total                  69.718             31.116            88.002

---------------------------------------------------------------------
Center     Atomic      Atomic             Coordinates (Angstroms)
Number     Number       Type             X           Y           Z
---------------------------------------------------------------------
     1          6           0        0.890869    0.466520    0.013976
     2          6           0        0.573057   -0.917266   -0.005282
     3          6           0       -1.640220   -0.545498   -0.012351
     4          6           0       -0.139725    1.444433    0.003103
     5          8           0       -0.115128    2.676678   -0.009854
     6          7           0        1.710891   -1.637695   -0.009702
     7          7           0       -0.708097   -1.435456    0.000419
     8          7           0       -1.421784    0.806402   -0.005156
     9          1           0       -2.197836    1.439395   -0.131357
    10          7           0        2.258249    0.603032    0.019353
    11          7           0       -2.999530   -0.931857   -0.075608
    12          1           0       -3.039415   -1.940250    0.017644
    13          1           0       -3.554625   -0.502823    0.657947
    14          6           0        2.668258   -0.667605    0.004649
    15          1           0        3.721359   -0.924232    0.004889
---------------------------------------------------------------------
```

Result for G-N9H_water.log

```
SCF Done:  E(RB3LYP) =  -542.323029872     A.U. after    1 cycles


Zero-point correction=                           0.103506 (Hartree/Particle)
Thermal correction to Energy=                    0.111386
Thermal correction to Enthalpy=                  0.112331
Thermal correction to Gibbs Free Energy=         0.070745
Sum of electronic and zero-point Energies=           -542.219524
Sum of electronic and thermal Energies=              -542.211643
Sum of electronic and thermal Enthalpies=            -542.210699
Sum of electronic and thermal Free Energies=         -542.252284

                    E (Thermal)             CV                S
                     KCal/Mol        Cal/Mol-Kelvin    Cal/Mol-Kelvin
Total                  69.896             31.040            87.524

---------------------------------------------------------------------
Center     Atomic      Atomic             Coordinates (Angstroms)
Number     Number       Type             X           Y           Z
---------------------------------------------------------------------
     1          6           0        0.875478    0.457868   -0.001360
     2          6           0        0.568366   -0.914563   -0.001323
     3          6           0       -1.652435   -0.551248   -0.004701
     4          6           0       -0.162513    1.409943   -0.000657
     5          8           0       -0.090069    2.660401    0.003882
```

6	7	0	1.721361	-1.626679	0.002001
7	7	0	-0.696358	-1.441105	0.002355
8	7	0	-1.425511	0.802216	-0.005569
9	1	0	-2.220698	1.430579	-0.015119
10	7	0	2.249307	0.612955	0.002133
11	7	0	-2.969018	-0.936746	-0.074618
12	1	0	-3.124445	-1.896841	0.201813
13	1	0	-3.642864	-0.292765	0.319882
14	6	0	2.674883	-0.651866	0.003472
15	1	0	3.727420	-0.899464	0.005677

Result for G_water.log

```
SCF Done:  E(RB3LYP) =  -542.797465831     A.U. after    1 cycles

Zero-point correction=                           0.116142 (Hartree/Particle)
Thermal correction to Energy=                    0.124373
Thermal correction to Enthalpy=                  0.125317
Thermal correction to Gibbs Free Energy=         0.083161
Sum of electronic and zero-point Energies=           -542.681324
Sum of electronic and thermal Energies=              -542.673093
Sum of electronic and thermal Enthalpies=            -542.672148
Sum of electronic and thermal Free Energies=         -542.714305

                    E (Thermal)             CV                S
                     KCal/Mol        Cal/Mol-Kelvin    Cal/Mol-Kelvin
Total                   78.045             32.728            88.725
```

Center Number	Atomic Number	Atomic Type	Coordinates (Angstroms) X	Y	Z
1	6	0	-0.835190	0.501630	0.001550
2	6	0	-0.534069	-0.856710	0.001158
3	6	0	1.673649	-0.572094	0.002965
4	6	0	0.234057	1.432482	0.000339
5	8	0	0.185316	2.671773	-0.002389
6	7	0	-1.744665	-1.489312	-0.001697
7	7	0	0.681818	-1.440095	-0.001632
8	7	0	1.477146	0.784226	0.001472
9	1	0	2.289203	1.392606	0.006971
10	7	0	-2.208947	0.697729	-0.000891
11	7	0	2.956857	-1.000858	0.058601
12	1	0	3.106351	-1.975021	-0.162352
13	1	0	3.688708	-0.366229	-0.229594
14	6	0	-2.711546	-0.512027	-0.002621
15	1	0	-3.760697	-0.758838	-0.004728
16	1	0	-1.902965	-2.488213	-0.002500

Result for H.log

```
SCF Done:  E(RB3LYP) =  -487.362520027     A.U. after    1 cycles


Zero-point correction=                           0.099715 (Hartree/Particle)
Thermal correction to Energy=                    0.106499
Thermal correction to Enthalpy=                  0.107443
Thermal correction to Gibbs Free Energy=         0.068252
Sum of electronic and zero-point Energies=           -487.262805
Sum of electronic and thermal Energies=              -487.256021
Sum of electronic and thermal Enthalpies=            -487.255077
Sum of electronic and thermal Free Energies=         -487.294268

                    E (Thermal)             CV                S
                     KCal/Mol        Cal/Mol-Kelvin    Cal/Mol-Kelvin
Total                   66.829             26.831             82.486

---------------------------------------------------------------------
Center     Atomic      Atomic             Coordinates (Angstroms)
Number     Number       Type             X           Y           Z
---------------------------------------------------------------------
     1          6           0        0.244552    0.572647    0.000037
     2          6           0        0.628954   -0.764691   -0.000287
     3          6           0       -1.423228   -1.580693   -0.000072
     4          6           0       -1.159235    0.896973   -0.000139
     5          8           0       -1.725338    1.968865   -0.000174
     6          7           0        1.997521   -0.744326    0.000309
     7          7           0       -0.157822   -1.871574   -0.000454
     8          7           0       -1.920522   -0.314989    0.000533
     9          1           0       -2.922488   -0.175482    0.000815
    10          7           0        1.350000    1.394286   -0.000207
    11          1           0       -2.155409   -2.380161    0.000317
    12          6           0        2.373760    0.581861    0.000253
    13          1           0        3.410740    0.879105    0.000248
    14          1           0        2.596803   -1.554747   -0.000014
---------------------------------------------------------------------
```

Result for H-N9H.log

```
SCF Done:  E(RB3LYP) =  -486.819190829     A.U. after    2 cycles


Zero-point correction=                           0.086170 (Hartree/Particle)
Thermal correction to Energy=                    0.092729
Thermal correction to Enthalpy=                  0.093673
Thermal correction to Gibbs Free Energy=         0.054845
Sum of electronic and zero-point Energies=           -486.733021
Sum of electronic and thermal Energies=              -486.726462
Sum of electronic and thermal Enthalpies=            -486.725518
Sum of electronic and thermal Free Energies=         -486.764346
```

	E (Thermal) KCal/Mol	CV Cal/Mol-Kelvin	S Cal/Mol-Kelvin
Total	58.188	25.587	81.720

Center Number	Atomic Number	Atomic Type	Coordinates (Angstroms) X	Y	Z
1	6	0	0.281081	0.565576	0.000047
2	6	0	0.703431	-0.793331	0.000047
3	6	0	-1.393700	-1.577408	0.000037
4	6	0	-1.105390	0.896926	0.000178
5	8	0	-1.694867	1.980143	-0.000084
6	7	0	2.052863	-0.838897	-0.000143
7	7	0	-0.139862	-1.882963	0.000067
8	7	0	-1.883634	-0.295929	0.000075
9	1	0	-2.881030	-0.140189	-0.000051
10	7	0	1.388497	1.369558	-0.000058
11	1	0	-2.146622	-2.361313	-0.000023
12	6	0	2.387331	0.476339	-0.000089
13	1	0	3.425027	0.789363	-0.000167

Result for H-N9H_water.log

SCF Done: E(RB3LYP) = -486.927393435 A.U. after 1 cycles

Zero-point correction= 0.087162 (Hartree/Particle)
Thermal correction to Energy= 0.093581
Thermal correction to Enthalpy= 0.094525
Thermal correction to Gibbs Free Energy= 0.055966
Sum of electronic and zero-point Energies= -486.840231
Sum of electronic and thermal Energies= -486.833812
Sum of electronic and thermal Enthalpies= -486.832868
Sum of electronic and thermal Free Energies= -486.871427

	E (Thermal) KCal/Mol	CV Cal/Mol-Kelvin	S Cal/Mol-Kelvin
Total	58.723	25.070	81.155

Center Number	Atomic Number	Atomic Type	Coordinates (Angstroms) X	Y	Z
1	6	0	0.281646	0.546930	-0.000156
2	6	0	0.685264	-0.802155	0.000055
3	6	0	-1.426143	-1.548247	0.000037
4	6	0	-1.093070	0.881188	-0.000558
5	8	0	-1.628621	2.012112	0.000230
6	7	0	2.039348	-0.861071	0.000049
7	7	0	-0.169154	-1.875581	0.000006
8	7	0	-1.892434	-0.266193	-0.000085
9	1	0	-2.895337	-0.120061	0.000109

```
    10          7           0        1.399351    1.348404    0.000655
    11          1           0       -2.191529   -2.313483    0.000251
    12          6           0        2.393110    0.449986   -0.000443
    13          1           0        3.431213    0.751520   -0.000182
---------------------------------------------------------------------
```

Result for H_water.log

```
SCF Done:  E(RB3LYP) =  -487.397760090     A.U. after    1 cycles


Zero-point correction=                           0.100100 (Hartree/Particle)
Thermal correction to Energy=                    0.106772
Thermal correction to Enthalpy=                  0.107716
Thermal correction to Gibbs Free Energy=         0.068742
Sum of electronic and zero-point Energies=          -487.297660
Sum of electronic and thermal Energies=             -487.290988
Sum of electronic and thermal Enthalpies=           -487.290044
Sum of electronic and thermal Free Energies=        -487.329018

                    E (Thermal)             CV                S
                     KCal/Mol        Cal/Mol-Kelvin    Cal/Mol-Kelvin
Total                   67.000             26.430             82.027

---------------------------------------------------------------------
Center     Atomic      Atomic             Coordinates (Angstroms)
Number     Number       Type             X           Y           Z
---------------------------------------------------------------------
     1          6           0        0.237279    0.560663   -0.000001
     2          6           0        0.632139   -0.772811   -0.000251
     3          6           0       -1.433510   -1.567349    0.000002
     4          6           0       -1.152789    0.870706   -0.000055
     5          8           0       -1.696430    1.982899   -0.000140
     6          7           0        1.996905   -0.743953    0.000196
     7          7           0       -0.165240   -1.869344   -0.000315
     8          7           0       -1.922569   -0.300530    0.000337
     9          1           0       -2.929859   -0.176986    0.000371
    10          7           0        1.340645    1.392678   -0.000088
    11          1           0       -2.177467   -2.352606    0.000461
    12          6           0        2.368113    0.573721    0.000190
    13          1           0        3.405050    0.867554    0.000178
    14          1           0        2.618143   -1.542697   -0.000107
---------------------------------------------------------------------
```

Result for N.log

```
SCF Done:  E(RB3LYP) =  -412.090048633     A.U. after    1 cycles


Zero-point correction=                           0.094654 (Hartree/Particle)
Thermal correction to Energy=                    0.100410
```

```
Thermal correction to Enthalpy=                  0.101354
Thermal correction to Gibbs Free Energy=         0.064624
Sum of electronic and zero-point Energies=           -411.995395
Sum of electronic and thermal Energies=              -411.989639
Sum of electronic and thermal Enthalpies=            -411.988695
Sum of electronic and thermal Free Energies=         -412.025424

                    E (Thermal)             CV                S
                     KCal/Mol        Cal/Mol-Kelvin    Cal/Mol-Kelvin
Total                   63.008             22.848            77.304

---------------------------------------------------------------------
Center     Atomic      Atomic             Coordinates (Angstroms)
Number     Number       Type             X           Y           Z
---------------------------------------------------------------------
     1          6           0        0.240475    0.739584    0.000000
     2          6           0        0.204599   -0.668273   -0.000000
     3          6           0       -2.007814   -0.687126    0.000000
     4          6           0       -0.993675    1.375642   -0.000000
     5          7           0        1.522599   -1.058508    0.000000
     6          7           0       -0.888266   -1.415782   -0.000000
     7          7           0       -2.113473    0.650290   -0.000000
     8          7           0        1.553273    1.189292    0.000001
     9          6           0        2.272432    0.100718   -0.000001
    10          1           0        1.861832   -2.007332    0.000001
    11          1           0        3.351649    0.070773   -0.000001
    12          1           0       -1.088582    2.456523    0.000000
    13          1           0       -2.939927   -1.240287   -0.000001
---------------------------------------------------------------------
```

Result for N-N9H.log

```
SCF Done:  E(RB3LYP) =  -411.548095748     A.U. after    1 cycles

Zero-point correction=                           0.081463 (Hartree/Particle)
Thermal correction to Energy=                    0.086903
Thermal correction to Enthalpy=                  0.087847
Thermal correction to Gibbs Free Energy=         0.051634
Sum of electronic and zero-point Energies=           -411.466633
Sum of electronic and thermal Energies=              -411.461192
Sum of electronic and thermal Enthalpies=            -411.460248
Sum of electronic and thermal Free Energies=         -411.496461

                    E (Thermal)             CV                S
                     KCal/Mol        Cal/Mol-Kelvin    Cal/Mol-Kelvin
Total                   54.533             21.297            76.217

---------------------------------------------------------------------
Center     Atomic      Atomic             Coordinates (Angstroms)
Number     Number       Type             X           Y           Z
---------------------------------------------------------------------
     1          6           0        0.286850    0.722827   -0.000000
     2          6           0        0.265376   -0.714098    0.000000
```

```
     3          6           0       -1.979615   -0.667523    0.000000
     4          6           0       -0.934074    1.372735   -0.000000
     5          7           0        1.543684   -1.172722   -0.000001
     6          7           0       -0.881023   -1.417510    0.000000
     7          7           0       -2.081405    0.675011   -0.000000
     8          7           0        1.593435    1.147515   -0.000000
     9          6           0        2.256961   -0.020242    0.000000
    10          1           0        3.341662   -0.041832    0.000001
    11          1           0       -1.011784    2.458183   -0.000000
    12          1           0       -2.925701   -1.204610   -0.000000
 ---------------------------------------------------------------------
```

Result for N-N9H_water.log

```
 SCF Done:  E(RB3LYP) =  -411.643734465     A.U. after    1 cycles

 Zero-point correction=                           0.082290 (Hartree/Particle)
 Thermal correction to Energy=                    0.087713
 Thermal correction to Enthalpy=                  0.088657
 Thermal correction to Gibbs Free Energy=         0.052468
 Sum of electronic and zero-point Energies=           -411.561445
 Sum of electronic and thermal Energies=              -411.556022
 Sum of electronic and thermal Enthalpies=            -411.555077
 Sum of electronic and thermal Free Energies=         -411.591266

                     E (Thermal)             CV                S
                      KCal/Mol        Cal/Mol-Kelvin    Cal/Mol-Kelvin
 Total                   55.041             21.153             76.165

 ---------------------------------------------------------------------
 Center     Atomic      Atomic             Coordinates (Angstroms)
 Number     Number       Type             X           Y           Z
 ---------------------------------------------------------------------
      1          6           0        0.283925    0.719800   -0.000000
      2          6           0        0.260495   -0.704264    0.000000
      3          6           0       -1.984411   -0.667869    0.000000
      4          6           0       -0.930603    1.375933   -0.000000
      5          7           0        1.537527   -1.171272   -0.000000
      6          7           0       -0.881049   -1.414009    0.000000
      7          7           0       -2.077467    0.673995   -0.000000
      8          7           0        1.597547    1.139897    0.000000
      9          6           0        2.263084   -0.024202   -0.000000
     10          1           0        3.344421   -0.051033    0.000002
     11          1           0       -1.008756    2.458589   -0.000000
     12          1           0       -2.926506   -1.204227   -0.000001
 ---------------------------------------------------------------------
```

Result for N_water.log

```
 SCF Done:  E(RB3LYP) =  -412.114260444     A.U. after    1 cycles
```

```
Zero-point correction=                           0.095130 (Hartree/Particle)
Thermal correction to Energy=                    0.100824
Thermal correction to Enthalpy=                  0.101769
Thermal correction to Gibbs Free Energy=         0.065142
Sum of electronic and zero-point Energies=           -412.019131
Sum of electronic and thermal Energies=              -412.013436
Sum of electronic and thermal Enthalpies=            -412.012492
Sum of electronic and thermal Free Energies=         -412.049118

                    E (Thermal)             CV                S
                     KCal/Mol        Cal/Mol-Kelvin    Cal/Mol-Kelvin
Total                  63.268             22.614             77.086

---------------------------------------------------------------------
Center     Atomic      Atomic             Coordinates (Angstroms)
Number     Number       Type             X           Y           Z
---------------------------------------------------------------------
     1          6           0        0.236009    0.739148   -0.000000
     2          6           0        0.211403   -0.667979   -0.000000
     3          6           0       -2.007462   -0.690440    0.000000
     4          6           0       -0.992337    1.374325   -0.000000
     5          7           0        1.524648   -1.052494    0.000000
     6          7           0       -0.886129   -1.416011   -0.000000
     7          7           0       -2.117875    0.647333   -0.000000
     8          7           0        1.548735    1.194031    0.000001
     9          6           0        2.269805    0.097838   -0.000001
    10          1           0        1.884961   -1.997738    0.000001
    11          1           0        3.347739    0.062412   -0.000001
    12          1           0       -1.086505    2.454227    0.000000
    13          1           0       -2.936357   -1.246275   -0.000001
---------------------------------------------------------------------
```

Result for T.log

```
SCF Done:  E(RB3LYP) =  -454.311416113     A.U. after    1 cycles


Zero-point correction=                           0.114433 (Hartree/Particle)
Thermal correction to Energy=                    0.122296
Thermal correction to Enthalpy=                  0.123240
Thermal correction to Gibbs Free Energy=         0.081967
Sum of electronic and zero-point Energies=           -454.196983
Sum of electronic and thermal Energies=              -454.189120
Sum of electronic and thermal Enthalpies=            -454.188176
Sum of electronic and thermal Free Energies=         -454.229450

                    E (Thermal)             CV                S
                     KCal/Mol        Cal/Mol-Kelvin    Cal/Mol-Kelvin
Total                  76.742             29.326             86.867

---------------------------------------------------------------------
Center     Atomic      Atomic             Coordinates (Angstroms)
```

Number	Number	Type	X	Y	Z
1	6	0	1.152185	-0.486788	0.000013
2	6	0	0.239768	-1.475578	-0.000072
3	6	0	-1.674332	0.026994	0.000032
4	6	0	0.669929	0.894580	-0.000054
5	8	0	1.381350	1.881492	0.000150
6	8	0	-2.871488	0.224463	0.000511
7	7	0	-1.116290	-1.239197	-0.000361
8	1	0	-1.777086	-2.000149	0.000297
9	7	0	-0.726105	1.030588	-0.000545
10	1	0	-1.085757	1.976274	-0.000500
11	6	0	2.630568	-0.716814	0.000177
12	1	0	3.094088	-0.256883	-0.875173
13	1	0	3.093775	-0.257940	0.876286
14	1	0	2.860939	-1.782891	-0.000301
15	1	0	0.523209	-2.520146	-0.000131

Result for T-N1H.log

```
SCF Done:  E(RB3LYP) =  -453.764688463     A.U. after    1 cycles

Zero-point correction=                           0.100520 (Hartree/Particle)
Thermal correction to Energy=                    0.108103
Thermal correction to Enthalpy=                  0.109048
Thermal correction to Gibbs Free Energy=         0.068214
Sum of electronic and zero-point Energies=           -453.664169
Sum of electronic and thermal Energies=              -453.656585
Sum of electronic and thermal Enthalpies=            -453.655641
Sum of electronic and thermal Free Energies=         -453.696474
```

	E (Thermal) KCal/Mol	CV Cal/Mol-Kelvin	S Cal/Mol-Kelvin
Total	67.836	27.818	85.941

Center Number	Atomic Number	Atomic Type	Coordinates (Angstroms) X	Y	Z
1	6	0	1.123424	-0.466707	0.000007
2	6	0	0.174871	-1.469236	-0.000111
3	6	0	-1.670334	-0.090852	-0.000071
4	6	0	0.644044	0.882664	0.000068
5	8	0	1.337827	1.914390	0.000209
6	8	0	-2.874905	0.192153	-0.000096
7	7	0	-1.159470	-1.351250	-0.000159
8	7	0	-0.743267	0.970453	0.000030
9	6	0	2.599921	-0.724707	0.000073
10	1	0	3.092080	-0.286681	-0.875703
11	1	0	3.091969	-0.286932	0.876039
12	1	0	2.799461	-1.800644	-0.000065
13	1	0	0.536809	-2.500487	-0.000164

```
   14          1           0       -1.136092    1.901006    0.000097
 ---------------------------------------------------------------------
```

Result for T-N1H_water.log

```
 SCF Done:  E(RB3LYP) =  -453.862026725     A.U. after    1 cycles

 Zero-point correction=                           0.100831 (Hartree/Particle)
 Thermal correction to Energy=                    0.108284
 Thermal correction to Enthalpy=                  0.109228
 Thermal correction to Gibbs Free Energy=         0.068763
 Sum of electronic and zero-point Energies=           -453.761195
 Sum of electronic and thermal Energies=              -453.753743
 Sum of electronic and thermal Enthalpies=            -453.752799
 Sum of electronic and thermal Free Energies=         -453.793264

                    E (Thermal)             CV                S
                     KCal/Mol        Cal/Mol-Kelvin    Cal/Mol-Kelvin
 Total                  67.949             27.629            85.166

 ---------------------------------------------------------------------
 Center     Atomic      Atomic             Coordinates (Angstroms)
 Number     Number       Type             X           Y           Z
 ---------------------------------------------------------------------
      1          6           0        1.124760   -0.463738    0.000002
      2          6           0        0.192606   -1.472848   -0.000034
      3          6           0       -1.646334   -0.090885   -0.000545
      4          6           0        0.626646    0.871534    0.000175
      5          8           0        1.321037    1.917249    0.000320
      6          8           0       -2.878159    0.171731    0.000149
      7          7           0       -1.151900   -1.342190   -0.000141
      8          7           0       -0.752910    0.971877   -0.000098
      9          6           0        2.603060   -0.710497    0.000031
     10          1           0        3.086176   -0.272690   -0.878719
     11          1           0        3.086085   -0.272978    0.878976
     12          1           0        2.809460   -1.782034   -0.000131
     13          1           0        0.551542   -2.498799   -0.000056
     14          1           0       -1.147057    1.905456    0.000072
 ---------------------------------------------------------------------
```

Result for T_water.log

```
 SCF Done:  E(RB3LYP) =  -454.334254198     A.U. after    1 cycles

 Zero-point correction=                           0.114187 (Hartree/Particle)
 Thermal correction to Energy=                    0.121899
 Thermal correction to Enthalpy=                  0.122843
 Thermal correction to Gibbs Free Energy=         0.081964
 Sum of electronic and zero-point Energies=           -454.220067
```

```
Sum of electronic and thermal Energies=            -454.212355
Sum of electronic and thermal Enthalpies=          -454.211411
Sum of electronic and thermal Free Energies=       -454.252290

                    E (Thermal)             CV                S
                     KCal/Mol        Cal/Mol-Kelvin    Cal/Mol-Kelvin
Total                  76.493              29.052             86.037

 ---------------------------------------------------------------------
 Center     Atomic      Atomic             Coordinates (Angstroms)
 Number     Number       Type             X           Y           Z
 ---------------------------------------------------------------------
      1          6           0        1.150286   -0.476451   -0.000094
      2          6           0        0.239253   -1.472264    0.000394
      3          6           0       -1.654738    0.015758   -0.000018
      4          6           0        0.658854    0.884884   -0.000108
      5          8           0        1.372092    1.893400    0.000280
      6          8           0       -2.867549    0.226256    0.000405
      7          7           0       -1.111597   -1.238026   -0.000278
      8          1           0       -1.759627   -2.015143   -0.000674
      9          7           0       -0.725037    1.026287   -0.000556
     10          1           0       -1.092294    1.971998   -0.000237
     11          6           0        2.626417   -0.717025    0.000014
     12          1           0        3.096115   -0.268651   -0.879008
     13          1           0        3.095869   -0.270644    0.880162
     14          1           0        2.838952   -1.786017   -0.001265
     15          1           0        0.520648   -2.516040    0.000250
 ---------------------------------------------------------------------
```

Result for U.log

```
SCF Done:  E(RB3LYP) =  -414.975340473     A.U. after    1 cycles

Zero-point correction=                           0.086755 (Hartree/Particle)
Thermal correction to Energy=                    0.092957
Thermal correction to Enthalpy=                  0.093901
Thermal correction to Gibbs Free Energy=         0.056299
Sum of electronic and zero-point Energies=           -414.888585
Sum of electronic and thermal Energies=              -414.882384
Sum of electronic and thermal Enthalpies=            -414.881439
Sum of electronic and thermal Free Energies=         -414.919041

                    E (Thermal)             CV                S
                     KCal/Mol        Cal/Mol-Kelvin    Cal/Mol-Kelvin
Total                  58.331              23.416             79.140

 ---------------------------------------------------------------------
 Center     Atomic      Atomic             Coordinates (Angstroms)
 Number     Number       Type             X           Y           Z
 ---------------------------------------------------------------------
      1          6           0       -1.200394    1.104868    0.000202
      2          6           0        0.002681    1.703428   -0.000173
      3          6           0        1.217211   -0.403570    0.000022
```

```
     4          6           0        -1.285929   -0.347196    0.000035
     5          8           0        -2.308154   -1.003298   -0.000168
     6          8           0         2.258547   -1.023752    0.000065
     7          7           0         1.171108    0.985138   -0.000140
     8          1           0         2.068834    1.443599   -0.000035
     9          7           0        -0.032485   -0.988124    0.000064
    10          1           0        -0.044355   -1.999942    0.000100
    11          1           0         0.119407    2.778904    0.000084
    12          1           0        -2.118808    1.669556    0.000695
 ---------------------------------------------------------------------
```

Result for U-N1H.log

```
 SCF Done:  E(RB3LYP) =  -414.431521701     A.U. after    9 cycles

 Zero-point correction=                           0.073067 (Hartree/Particle)
 Thermal correction to Energy=                    0.078949
 Thermal correction to Enthalpy=                  0.079893
 Thermal correction to Gibbs Free Energy=         0.042871
 Sum of electronic and zero-point Energies=           -414.358455
 Sum of electronic and thermal Energies=              -414.352573
 Sum of electronic and thermal Enthalpies=            -414.351629
 Sum of electronic and thermal Free Energies=         -414.388650

                     E (Thermal)             CV                S
                      KCal/Mol        Cal/Mol-Kelvin    Cal/Mol-Kelvin
 Total                  49.541             21.961             77.918

 ---------------------------------------------------------------------
 Center     Atomic      Atomic             Coordinates (Angstroms)
 Number     Number       Type             X           Y           Z
 ---------------------------------------------------------------------
     1          6           0        -1.174912    1.097725    0.000099
     2          6           0         0.075950    1.679659    0.000046
     3          6           0         1.253025   -0.298820   -0.000080
     4          6           0        -1.261767   -0.327863    0.000061
     5          8           0        -2.287756   -1.027319    0.000099
     6          8           0         2.260581   -1.015314   -0.000158
     7          7           0         1.263849    1.064333   -0.000039
     8          7           0        -0.005771   -0.931916   -0.000029
     9          1           0         0.008912   -1.941968   -0.000059
    10          1           0         0.132170    2.769801    0.000076
    11          1           0        -2.084008    1.682109    0.000168
 ---------------------------------------------------------------------
```

Result for U-N1H_water.log

```
 SCF Done:  E(RB3LYP) =  -414.528946741     A.U. after    1 cycles
```

```
Zero-point correction=                           0.073097 (Hartree/Particle)
Thermal correction to Energy=                    0.078937
Thermal correction to Enthalpy=                  0.079881
Thermal correction to Gibbs Free Energy=         0.042972
Sum of electronic and zero-point Energies=           -414.455850
Sum of electronic and thermal Energies=              -414.450009
Sum of electronic and thermal Enthalpies=            -414.449065
Sum of electronic and thermal Free Energies=         -414.485974

                    E (Thermal)             CV                S
                     KCal/Mol        Cal/Mol-Kelvin    Cal/Mol-Kelvin
Total                  49.534             21.834             77.682

---------------------------------------------------------------------
Center     Atomic      Atomic             Coordinates (Angstroms)
Number     Number       Type             X           Y           Z
---------------------------------------------------------------------
     1          6           0       -1.176966    1.088703    0.000123
     2          6           0        0.058343    1.687837    0.000025
     3          6           0        1.230773   -0.291122    0.000259
     4          6           0       -1.239105   -0.328887    0.000090
     5          8           0       -2.276379   -1.034994    0.000005
     6          8           0        2.270501   -0.995499   -0.000428
     7          7           0        1.249851    1.058931   -0.000016
     8          7           0        0.002636   -0.941402    0.000067
     9          1           0        0.020768   -1.954614   -0.000102
    10          1           0        0.111505    2.772469   -0.000010
    11          1           0       -2.090927    1.664207    0.000148
---------------------------------------------------------------------
```

Result for U_water.log

```
SCF Done:  E(RB3LYP) =  -414.999294283     A.U. after    1 cycles


Zero-point correction=                           0.086373 (Hartree/Particle)
Thermal correction to Energy=                    0.092495
Thermal correction to Enthalpy=                  0.093439
Thermal correction to Gibbs Free Energy=         0.056029
Sum of electronic and zero-point Energies=           -414.912922
Sum of electronic and thermal Energies=              -414.906799
Sum of electronic and thermal Enthalpies=            -414.905855
Sum of electronic and thermal Free Energies=         -414.943265

                    E (Thermal)             CV                S
                     KCal/Mol        Cal/Mol-Kelvin    Cal/Mol-Kelvin
Total                  58.041             23.232             78.735

---------------------------------------------------------------------
Center     Atomic      Atomic             Coordinates (Angstroms)
Number     Number       Type             X           Y           Z
---------------------------------------------------------------------
     1          6           0       -1.193985    1.102543   -0.000062
     2          6           0        0.014425    1.700215   -0.000028
```

3	6	0	1.201958	-0.393627	0.000013
4	6	0	-1.270999	-0.333391	-0.000027
5	8	0	-2.310711	-0.999730	0.000030
6	8	0	2.246841	-1.039475	-0.000088
7	7	0	1.173848	0.977857	0.000105
8	1	0	2.069584	1.449444	0.000031
9	7	0	-0.038180	-0.983909	0.000104
10	1	0	-0.049564	-1.998323	-0.000210
11	1	0	0.139696	2.773549	0.000036
12	1	0	-2.106825	1.676897	-0.000227

Result for X_26diketoN7H.log

```
SCF Done:  E(RB3LYP) =  -562.648947225     A.U. after    1 cycles

Zero-point correction=                           0.104614 (Hartree/Particle)
Thermal correction to Energy=                    0.112588
Thermal correction to Enthalpy=                  0.113532
Thermal correction to Gibbs Free Energy=         0.071526
Sum of electronic and zero-point Energies=          -562.544334
Sum of electronic and thermal Energies=             -562.536359
Sum of electronic and thermal Enthalpies=           -562.535415
Sum of electronic and thermal Free Energies=        -562.577421
```

	E (Thermal) KCal/Mol	CV Cal/Mol-Kelvin	S Cal/Mol-Kelvin
Total	70.650	31.043	88.409

Center Number	Atomic Number	Atomic Type	Coordinates (Angstroms) X	Y	Z
1	6	0	0.769563	0.460096	-0.000147
2	6	0	0.542073	-0.896823	0.000114
3	6	0	-1.837860	-0.570521	-0.000111
4	6	0	-0.282355	1.431931	0.000014
5	6	0	2.649883	-0.672029	-0.000105
6	8	0	-0.174871	2.644937	0.000504
7	8	0	-2.977520	-0.984284	-0.000141
8	7	0	1.703470	-1.601542	0.000168
9	7	0	-0.731821	-1.403839	0.000364
10	1	0	-0.903126	-2.398124	0.000837
11	7	0	-1.536719	0.793025	-0.000439
12	1	0	-2.345593	1.400344	-0.000275
13	7	0	2.144538	0.585622	-0.000264
14	1	0	2.654392	1.455216	-0.000459
15	1	0	3.709359	-0.871451	-0.000391

Result for X_26diketoN7H_water.log

```
SCF Done:  E(RB3LYP) =  -562.680477714     A.U. after    1 cycles

Zero-point correction=                           0.104181 (Hartree/Particle)
Thermal correction to Energy=                    0.112067
Thermal correction to Enthalpy=                  0.113011
Thermal correction to Gibbs Free Energy=         0.071260
Sum of electronic and zero-point Energies=          -562.576296
Sum of electronic and thermal Energies=             -562.568411
Sum of electronic and thermal Enthalpies=           -562.567467
Sum of electronic and thermal Free Energies=        -562.609218

                    E (Thermal)             CV                S
                     KCal/Mol        Cal/Mol-Kelvin    Cal/Mol-Kelvin
Total                   70.323            30.881            87.873

---------------------------------------------------------------------
Center     Atomic      Atomic             Coordinates (Angstroms)
Number     Number       Type             X           Y           Z
---------------------------------------------------------------------
     1          6           0        0.771309    0.464154    0.000157
     2          6           0        0.541281   -0.895145    0.000243
     3          6           0       -1.817833   -0.568680   -0.000005
     4          6           0       -0.284406    1.414311    0.000044
     5          6           0        2.648967   -0.660277   -0.000287
     6          8           0       -0.194882    2.644497    0.000074
     7          8           0       -2.975456   -0.985864   -0.000526
     8          7           0        1.700867   -1.599542    0.000039
     9          7           0       -0.731177   -1.401085    0.000533
    10          1           0       -0.902317   -2.399326    0.000071
    11          7           0       -1.534726    0.786075    0.000131
    12          1           0       -2.345916    1.394915   -0.000003
    13          7           0        2.146525    0.587883   -0.000224
    14          1           0        2.675878    1.450053   -0.000412
    15          1           0        3.708729   -0.854205   -0.000305
---------------------------------------------------------------------
```

Result for X_2enol6enol(a).log

```
SCF Done:  E(RB3LYP) =  -562.620948265     A.U. after    1 cycles

Zero-point correction=                           0.104385 (Hartree/Particle)
Thermal correction to Energy=                    0.112172
Thermal correction to Enthalpy=                  0.113116
Thermal correction to Gibbs Free Energy=         0.071749
Sum of electronic and zero-point Energies=          -562.516563
Sum of electronic and thermal Energies=             -562.508776
Sum of electronic and thermal Enthalpies=           -562.507832
Sum of electronic and thermal Free Energies=        -562.549199
```

```
                    E (Thermal)             CV                S
                     KCal/Mol        Cal/Mol-Kelvin    Cal/Mol-Kelvin
Total                   70.389             31.058            87.064

 ---------------------------------------------------------------------
 Center     Atomic      Atomic             Coordinates (Angstroms)
 Number     Number       Type             X           Y           Z
 ---------------------------------------------------------------------
      1          6           0       -0.780892    0.436658    0.000009
      2          6           0       -0.454594   -0.919309    0.000143
      3          6           0        1.707424   -0.467396   -0.000039
      4          6           0        0.302561    1.321985   -0.000016
      5          6           0       -2.643760   -0.587825   -0.000097
      6          8           0        0.141728    2.651478    0.000077
      7          8           0        2.990404   -0.867188   -0.000040
      8          7           0       -1.665000   -1.567498   -0.000195
      9          7           0        0.777856   -1.424842    0.000173
     10          7           0        1.538214    0.858767   -0.000145
     11          7           0       -2.153816    0.621633   -0.000021
     12          1           0       -3.692997   -0.838704   -0.000143
     13          1           0       -0.807878    2.843175    0.000378
     14          1           0        2.982434   -1.834529   -0.000117
     15          1           0       -1.803824   -2.565358    0.000913
 ---------------------------------------------------------------------
```

Result for X_2enol6enol(a)-N9H.log

```
 SCF Done:  E(RB3LYP) =  -562.081952752     A.U. after    1 cycles

 Zero-point correction=                           0.091307 (Hartree/Particle)
 Thermal correction to Energy=                    0.098809
 Thermal correction to Enthalpy=                  0.099753
 Thermal correction to Gibbs Free Energy=         0.058846
 Sum of electronic and zero-point Energies=           -561.990646
 Sum of electronic and thermal Energies=              -561.983144
 Sum of electronic and thermal Enthalpies=            -561.982199
 Sum of electronic and thermal Free Energies=         -562.023107

                    E (Thermal)             CV                S
                     KCal/Mol        Cal/Mol-Kelvin    Cal/Mol-Kelvin
Total                   62.004             29.570            86.097

 ---------------------------------------------------------------------
 Center     Atomic      Atomic             Coordinates (Angstroms)
 Number     Number       Type             X           Y           Z
 ---------------------------------------------------------------------
      1          6           0       -0.819580    0.381007    0.000020
      2          6           0       -0.473526   -0.992807   -0.000074
      3          6           0        1.690154   -0.410366    0.000007
      4          6           0        0.215836    1.298842    0.000090
      5          6           0       -2.577237   -0.769264   -0.000025
      6          8           0       -0.028443    2.640882    0.000137
```

7	8	0	3.014910	-0.773386	-0.000036
8	7	0	-1.611554	-1.727784	-0.000088
9	7	0	0.820838	-1.403729	-0.000075
10	7	0	1.485286	0.913011	0.000076
11	7	0	-2.190616	0.514809	-0.000039
12	1	0	-3.626772	-1.040116	-0.000059
13	1	0	-0.994309	2.721457	0.000125
14	1	0	2.987786	-1.739946	-0.000107

Result for X_2enol6enol(a)-N9H_water.log

SCF Done: E(RB3LYP) = -562.183074597 A.U. after 1 cycles

```
Zero-point correction=                           0.090357 (Hartree/Particle)
Thermal correction to Energy=                    0.098090
Thermal correction to Enthalpy=                  0.099034
Thermal correction to Gibbs Free Energy=         0.057718
Sum of electronic and zero-point Energies=           -562.092717
Sum of electronic and thermal Energies=              -562.084985
Sum of electronic and thermal Enthalpies=            -562.084041
Sum of electronic and thermal Free Energies=         -562.125357
```

	E (Thermal) KCal/Mol	CV Cal/Mol-Kelvin	S Cal/Mol-Kelvin
Total	61.552	30.215	86.957

Center Number	Atomic Number	Atomic Type	Coordinates (Angstroms) X	Y	Z
1	6	0	-0.828150	0.403198	-0.000028
2	6	0	-0.495501	-0.969441	-0.000028
3	6	0	1.677860	-0.455012	0.000051
4	6	0	0.228790	1.298764	0.000029
5	6	0	-2.602732	-0.739345	0.000178
6	8	0	0.075254	2.649267	0.000129
7	8	0	2.998699	-0.811791	0.000042
8	7	0	-1.637744	-1.700432	-0.000382
9	7	0	0.782431	-1.423151	-0.000040
10	7	0	1.489018	0.871223	0.000083
11	7	0	-2.204112	0.537091	-0.000055
12	1	0	-3.651275	-1.003377	0.000049
13	1	0	-0.870614	2.861307	0.000194
14	1	0	3.041516	-1.779839	-0.000068

Result for X_2enol6enol(a)_water.log

SCF Done: E(RB3LYP) = -562.654480372 A.U. after 1 cycles

```
Zero-point correction=                           0.103328 (Hartree/Particle)
Thermal correction to Energy=                    0.111278
Thermal correction to Enthalpy=                  0.112222
Thermal correction to Gibbs Free Energy=         0.070572
Sum of electronic and zero-point Energies=           -562.551152
Sum of electronic and thermal Energies=              -562.543202
Sum of electronic and thermal Enthalpies=            -562.542258
Sum of electronic and thermal Free Energies=         -562.583909

                    E (Thermal)             CV                S
                     KCal/Mol        Cal/Mol-Kelvin    Cal/Mol-Kelvin
Total                   69.828             31.522             87.661

 ---------------------------------------------------------------------
 Center     Atomic      Atomic             Coordinates (Angstroms)
 Number     Number       Type             X           Y           Z
 ---------------------------------------------------------------------
      1          6           0       -0.785685    0.452635    0.000007
      2          6           0       -0.473781   -0.909467    0.000042
      3          6           0        1.694831   -0.492542    0.000023
      4          6           0        0.306502    1.318260    0.000047
      5          6           0       -2.649903   -0.576933   -0.000034
      6          8           0        0.201438    2.658653    0.000004
      7          8           0        2.986615   -0.893906    0.000021
      8          7           0       -1.679978   -1.550856   -0.000124
      9          7           0        0.756545   -1.431417    0.000047
     10          7           0        1.543050    0.836684   -0.000042
     11          7           0       -2.161931    0.639309    0.000056
     12          1           0       -3.697530   -0.831304   -0.000033
     13          1           0       -0.732869    2.919348   -0.000254
     14          1           0        3.007237   -1.863687   -0.000392
     15          1           0       -1.836854   -2.550082    0.000411
 ---------------------------------------------------------------------
```

Result for X_2enol6enol(b).log

```
SCF Done:  E(RB3LYP) =  -562.622249383     A.U. after    1 cycles


Zero-point correction=                           0.104429 (Hartree/Particle)
Thermal correction to Energy=                    0.112203
Thermal correction to Enthalpy=                  0.113147
Thermal correction to Gibbs Free Energy=         0.071810
Sum of electronic and zero-point Energies=           -562.517820
Sum of electronic and thermal Energies=              -562.510046
Sum of electronic and thermal Enthalpies=            -562.509102
Sum of electronic and thermal Free Energies=         -562.550440

                    E (Thermal)             CV                S
                     KCal/Mol        Cal/Mol-Kelvin    Cal/Mol-Kelvin
Total                   70.408             31.020             87.002
```

```
 ---------------------------------------------------------------------
 Center     Atomic      Atomic             Coordinates (Angstroms)
 Number     Number       Type             X           Y           Z
 ---------------------------------------------------------------------
      1          6           0       -0.770209    0.452348    0.000023
      2          6           0       -0.491729   -0.915988   -0.000038
      3          6           0        1.676581   -0.562737   -0.000023
      4          6           0        0.343144    1.296135   -0.000009
      5          6           0       -2.667774   -0.504255    0.000069
      6          8           0        0.239456    2.631734   -0.000068
      7          8           0        2.932939   -1.038680    0.000065
      8          7           0       -1.725924   -1.518094   -0.000230
      9          7           0        0.715065   -1.476439   -0.000074
     10          7           0        1.558385    0.777862    0.000047
     11          7           0       -2.135225    0.687590    0.000141
     12          1           0       -3.725551   -0.716415    0.000031
     13          1           0       -0.701319    2.862946   -0.000155
     14          1           0        3.520074   -0.269537    0.000128
     15          1           0       -1.898561   -2.510879    0.000695
 ---------------------------------------------------------------------
```

Result for X_2enol6enol(b)-N9H.log

```
 SCF Done:  E(RB3LYP) =  -562.080841623     A.U. after    1 cycles

 Zero-point correction=                           0.091208 (Hartree/Particle)
 Thermal correction to Energy=                    0.098743
 Thermal correction to Enthalpy=                  0.099688
 Thermal correction to Gibbs Free Energy=         0.058729
 Sum of electronic and zero-point Energies=           -561.989633
 Sum of electronic and thermal Energies=              -561.982098
 Sum of electronic and thermal Enthalpies=            -561.981154
 Sum of electronic and thermal Free Energies=         -562.022113

                     E (Thermal)             CV                S
                      KCal/Mol        Cal/Mol-Kelvin    Cal/Mol-Kelvin
 Total                  61.962             29.674             86.206

 ---------------------------------------------------------------------
 Center     Atomic      Atomic             Coordinates (Angstroms)
 Number     Number       Type             X           Y           Z
 ---------------------------------------------------------------------
      1          6           0       -0.807988    0.400362   -0.000007
      2          6           0       -0.522946   -0.989371   -0.000021
      3          6           0        1.656373   -0.527794    0.000037
      4          6           0        0.266018    1.269792    0.000018
      5          6           0       -2.615523   -0.667177    0.000020
      6          8           0        0.090909    2.622996   -0.000003
      7          8           0        2.966441   -0.944312    0.000026
      8          7           0       -1.695785   -1.668308   -0.000031
      9          7           0        0.747229   -1.471563    0.000005
     10          7           0        1.512708    0.815163    0.000034
     11          7           0       -2.170532    0.598544   -0.000074
```

12	1	0	-3.676600	-0.888852	-0.000002
13	1	0	-0.869791	2.751699	-0.000027
14	1	0	3.476659	-0.124030	0.000032

Result for X_2enol6enol(b)-N9H_water.log

```
SCF Done:  E(RB3LYP) =  -562.182907769     A.U. after    1 cycles

Zero-point correction=                           0.090608 (Hartree/Particle)
Thermal correction to Energy=                    0.098255
Thermal correction to Enthalpy=                  0.099199
Thermal correction to Gibbs Free Energy=         0.058054
Sum of electronic and zero-point Energies=          -562.092300
Sum of electronic and thermal Energies=             -562.084653
Sum of electronic and thermal Enthalpies=           -562.083709
Sum of electronic and thermal Free Energies=        -562.124854

                     E (Thermal)             CV                S
                      KCal/Mol        Cal/Mol-Kelvin    Cal/Mol-Kelvin
Total                    61.656             30.030            86.597
```

Center Number	Atomic Number	Atomic Type	Coordinates (Angstroms) X	Y	Z
1	6	0	-0.818216	0.418334	-0.000042
2	6	0	-0.533308	-0.965516	-0.000017
3	6	0	1.653883	-0.534579	0.000053
4	6	0	0.268575	1.276351	-0.000008
5	6	0	-2.631663	-0.660241	0.000285
6	8	0	0.164994	2.631067	0.000009
7	8	0	2.942476	-0.995593	0.000102
8	7	0	-1.701582	-1.654870	-0.000307
9	7	0	0.725784	-1.468308	-0.000010
10	7	0	1.511684	0.800025	0.000056
11	7	0	-2.188169	0.601472	-0.000135
12	1	0	-3.688968	-0.886840	0.000162
13	1	0	-0.772904	2.875996	-0.000037
14	1	0	3.532458	-0.227273	0.000133

Result for X_2enol6enol(b)_water.log

```
SCF Done:  E(RB3LYP) =  -562.654560822     A.U. after    1 cycles

Zero-point correction=                           0.103476 (Hartree/Particle)
Thermal correction to Energy=                    0.111363
Thermal correction to Enthalpy=                  0.112307
```

```
Thermal correction to Gibbs Free Energy=        0.070767
Sum of electronic and zero-point Energies=          -562.551085
Sum of electronic and thermal Energies=             -562.543198
Sum of electronic and thermal Enthalpies=           -562.542254
Sum of electronic and thermal Free Energies=        -562.583793

                   E (Thermal)             CV                S
                    KCal/Mol        Cal/Mol-Kelvin    Cal/Mol-Kelvin
Total                 69.881             31.356             87.427
```

Center Number	Atomic Number	Atomic Type	X	Y	Z
1	6	0	-0.775656	0.465788	0.000058
2	6	0	-0.507167	-0.906428	0.000037
3	6	0	1.670325	-0.566745	0.000011
4	6	0	0.343171	1.295537	0.000055
5	6	0	-2.671980	-0.502841	-0.000032
6	8	0	0.286027	2.638151	-0.000042
7	8	0	2.924675	-1.073384	-0.000052
8	7	0	-1.734294	-1.507452	-0.000124
9	7	0	0.702743	-1.472764	-0.000004
10	7	0	1.562081	0.768626	0.000111
11	7	0	-2.145076	0.696995	0.000019
12	1	0	-3.727336	-0.723144	-0.000252
13	1	0	-0.638513	2.931623	-0.000185
14	1	0	3.553798	-0.335316	-0.000318
15	1	0	-1.923899	-2.500988	0.000717

Result for X_2enol6enol(c).log

```
SCF Done:  E(RB3LYP) =  -562.622611670     A.U. after    1 cycles

Zero-point correction=                          0.104333 (Hartree/Particle)
Thermal correction to Energy=                   0.112101
Thermal correction to Enthalpy=                 0.113045
Thermal correction to Gibbs Free Energy=        0.071716
Sum of electronic and zero-point Energies=          -562.518279
Sum of electronic and thermal Energies=             -562.510511
Sum of electronic and thermal Enthalpies=           -562.509567
Sum of electronic and thermal Free Energies=        -562.550896

                   E (Thermal)             CV                S
                    KCal/Mol        Cal/Mol-Kelvin    Cal/Mol-Kelvin
Total                 70.344             31.041             86.985
```

Center Number	Atomic Number	Atomic Type	X	Y	Z
1	6	0	-0.791352	0.490573	0.000059

2	6	0	-0.520388	-0.884173	-0.000001
3	6	0	1.664075	-0.558001	-0.000075
4	6	0	0.343177	1.308433	-0.000009
5	6	0	-2.690876	-0.467216	-0.000020
6	8	0	0.237283	2.639234	-0.000043
7	8	0	2.926420	-1.021049	0.000090
8	7	0	-1.750651	-1.485932	-0.000255
9	7	0	0.684251	-1.456734	0.000058
10	7	0	1.557098	0.774142	-0.000084
11	7	0	-2.158580	0.721082	0.000158
12	1	0	-3.749067	-0.678425	0.000089
13	1	0	1.140642	2.992462	0.000103
14	1	0	2.869473	-1.986793	-0.000017
15	1	0	-1.923308	-2.478330	0.000587

Result for X_2enol6enol(c)-N9H.log

```
SCF Done:  E(RB3LYP) =  -562.079494052     A.U. after    1 cycles

Zero-point correction=                           0.091087 (Hartree/Particle)
Thermal correction to Energy=                    0.098607
Thermal correction to Enthalpy=                  0.099551
Thermal correction to Gibbs Free Energy=         0.058626
Sum of electronic and zero-point Energies=          -561.988407
Sum of electronic and thermal Energies=             -561.980887
Sum of electronic and thermal Enthalpies=           -561.979943
Sum of electronic and thermal Free Energies=        -562.020868
```

	E (Thermal) KCal/Mol	CV Cal/Mol-Kelvin	S Cal/Mol-Kelvin
Total	61.877	29.702	86.134

Center Number	Atomic Number	Atomic Type	Coordinates (Angstroms) X	Y	Z
1	6	0	-0.838166	0.454228	0.000017
2	6	0	-0.562910	-0.949410	-0.000028
3	6	0	1.636208	-0.527535	0.000004
4	6	0	0.265467	1.288192	0.000033
5	6	0	-2.647479	-0.618312	-0.000008
6	8	0	0.157081	2.645449	0.000034
7	8	0	2.935268	-0.975077	-0.000035
8	7	0	-1.729759	-1.626342	0.000011
9	7	0	0.699686	-1.450829	-0.000025
10	7	0	1.510707	0.803080	0.000020
11	7	0	-2.204449	0.641461	-0.000014
12	1	0	-3.709116	-0.839123	-0.000018
13	1	0	1.073220	2.959465	0.000045
14	1	0	2.845097	-1.937893	-0.000068

Result for X_2enol6enol(c)-N9H_water.log

```
SCF Done:  E(RB3LYP) =  -562.183332819     A.U. after    1 cycles

Zero-point correction=                           0.090480 (Hartree/Particle)
Thermal correction to Energy=                    0.098120
Thermal correction to Enthalpy=                  0.099064
Thermal correction to Gibbs Free Energy=         0.057936
Sum of electronic and zero-point Energies=           -562.092853
Sum of electronic and thermal Energies=              -562.085213
Sum of electronic and thermal Enthalpies=            -562.084268
Sum of electronic and thermal Free Energies=         -562.125396

                    E (Thermal)             CV                S
                     KCal/Mol        Cal/Mol-Kelvin    Cal/Mol-Kelvin
Total                   61.571             30.051             86.561

---------------------------------------------------------------------
Center     Atomic      Atomic             Coordinates (Angstroms)
Number     Number       Type             X           Y           Z
---------------------------------------------------------------------
     1          6           0       -0.830745    0.438190   -0.000004
     2          6           0       -0.547536   -0.946961    0.000003
     3          6           0        1.645870   -0.521642   -0.000155
     4          6           0        0.262267    1.289364   -0.000018
     5          6           0       -2.644226   -0.643068    0.000105
     6          8           0        0.091852    2.633744    0.000079
     7          8           0        2.951635   -0.929410    0.000109
     8          7           0       -1.713860   -1.637789   -0.000144
     9          7           0        0.712014   -1.450847   -0.000081
    10          7           0        1.506311    0.811221   -0.000056
    11          7           0       -2.202059    0.618253    0.000079
    12          1           0       -3.701397   -0.870449    0.000091
    13          1           0        0.967117    3.053201    0.000121
    14          1           0        2.955766   -1.898586    0.000117
---------------------------------------------------------------------
```

Result for X_2enol6enol(c)_water.log

```
SCF Done:  E(RB3LYP) =  -562.655180615     A.U. after    1 cycles

Zero-point correction=                           0.103465 (Hartree/Particle)
Thermal correction to Energy=                    0.111329
Thermal correction to Enthalpy=                  0.112273
Thermal correction to Gibbs Free Energy=         0.070795
Sum of electronic and zero-point Energies=           -562.551716
Sum of electronic and thermal Energies=              -562.543852
Sum of electronic and thermal Enthalpies=            -562.542907
Sum of electronic and thermal Free Energies=         -562.584385
```

	E (Thermal) KCal/Mol	CV Cal/Mol-Kelvin	S Cal/Mol-Kelvin
Total	69.860	31.348	87.298

Center Number	Atomic Number	Atomic Type	X	Y	Z
1	6	0	-0.786016	0.482927	-0.000007
2	6	0	-0.518684	-0.889121	0.000100
3	6	0	1.664726	-0.553496	-0.000570
4	6	0	0.337841	1.307259	-0.000510
5	6	0	-2.681490	-0.487785	-0.000360
6	8	0	0.197378	2.640459	0.001048
7	8	0	2.941320	-0.999792	0.000009
8	7	0	-1.743691	-1.492138	0.000585
9	7	0	0.692949	-1.455349	0.000314
10	7	0	1.557777	0.781665	-0.000672
11	7	0	-2.156045	0.712591	-0.000693
12	1	0	-3.736573	-0.709153	-0.000127
13	1	0	1.076930	3.053370	0.000500
14	1	0	2.926394	-1.969804	0.001164
15	1	0	-1.931524	-2.485847	0.001344

Result for X_2enol6enol(d).log

SCF Done: E(RB3LYP) = -562.622281247 A.U. after 1 cycles

Zero-point correction=	0.104275 (Hartree/Particle)
Thermal correction to Energy=	0.112060
Thermal correction to Enthalpy=	0.113004
Thermal correction to Gibbs Free Energy=	0.071654
Sum of electronic and zero-point Energies=	-562.518007
Sum of electronic and thermal Energies=	-562.510221
Sum of electronic and thermal Enthalpies=	-562.509277
Sum of electronic and thermal Free Energies=	-562.550627

	E (Thermal) KCal/Mol	CV Cal/Mol-Kelvin	S Cal/Mol-Kelvin
Total	70.319	31.113	87.028

Center Number	Atomic Number	Atomic Type	X	Y	Z
1	6	0	-0.781138	0.503055	0.000069
2	6	0	-0.551858	-0.880920	-0.000044
3	6	0	1.632584	-0.641540	0.000000
4	6	0	0.376564	1.283953	-0.000003
5	6	0	-2.708515	-0.395394	0.000073
6	8	0	0.315780	2.618557	-0.000098
7	8	0	2.865907	-1.177598	0.000063

8	7	0	-1.801264	-1.442223	-0.000263
9	7	0	0.627459	-1.500297	-0.000080
10	7	0	1.570518	0.702338	0.000010
11	7	0	-2.140260	0.776652	0.000158
12	1	0	-3.772907	-0.572936	0.000024
13	1	0	1.227955	2.946062	-0.000065
14	1	0	3.492950	-0.441574	0.000286
15	1	0	-2.002496	-2.429445	0.000686

Result for X_2enol6enol(d)-N9H.log

```
SCF Done:  E(RB3LYP) =  -562.076834801     A.U. after    1 cycles
```

```
Zero-point correction=                           0.090862 (Hartree/Particle)
Thermal correction to Energy=                    0.098460
Thermal correction to Enthalpy=                  0.099404
Thermal correction to Gibbs Free Energy=         0.058353
Sum of electronic and zero-point Energies=           -561.985972
Sum of electronic and thermal Energies=              -561.978375
Sum of electronic and thermal Enthalpies=            -561.977431
Sum of electronic and thermal Free Energies=         -562.018482
```

	E (Thermal) KCal/Mol	CV Cal/Mol-Kelvin	S Cal/Mol-Kelvin
Total	61.784	29.937	86.399

Center Number	Atomic Number	Atomic Type	Coordinates (Angstroms) X	Y	Z
1	6	0	-0.826547	0.470292	-0.000013
2	6	0	-0.605744	-0.945027	-0.000024
3	6	0	1.598934	-0.631168	0.000036
4	6	0	0.307257	1.259674	0.000018
5	6	0	-2.676405	-0.527804	0.000010
6	8	0	0.256777	2.621413	0.000003
7	8	0	2.881859	-1.127833	0.000041
8	7	0	-1.800963	-1.571306	-0.000045
9	7	0	0.631036	-1.508133	0.000006
10	7	0	1.527887	0.714770	0.000036
11	7	0	-2.183217	0.713774	-0.000081
12	1	0	-3.746239	-0.705065	-0.000020
13	1	0	1.183315	2.901044	0.000026
14	1	0	3.445667	-0.344158	0.000067

Result for X_2enol6enol(d)-N9H_water.log

```
SCF Done:  E(RB3LYP) =  -562.183004078     A.U. after    1 cycles
```

```
Zero-point correction=                           0.090412 (Hartree/Particle)
Thermal correction to Energy=                    0.098081
Thermal correction to Enthalpy=                  0.099025
Thermal correction to Gibbs Free Energy=         0.057847
Sum of electronic and zero-point Energies=           -562.092592
Sum of electronic and thermal Energies=              -562.084923
Sum of electronic and thermal Enthalpies=            -562.083979
Sum of electronic and thermal Free Energies=         -562.125158

                    E (Thermal)             CV                S
                     KCal/Mol        Cal/Mol-Kelvin    Cal/Mol-Kelvin
Total                  61.547            30.114            86.668

---------------------------------------------------------------------
Center     Atomic      Atomic             Coordinates (Angstroms)
Number     Number       Type             X           Y           Z
---------------------------------------------------------------------
     1          6           0       -0.821101    0.452398   -0.000063
     2          6           0       -0.583489   -0.942035   -0.000015
     3          6           0        1.619661   -0.596958    0.000136
     4          6           0        0.298979    1.266774   -0.000014
     5          6           0       -2.669470   -0.567365    0.000406
     6          8           0        0.176942    2.616196   -0.000021
     7          8           0        2.890006   -1.105358    0.000112
     8          7           0       -1.773142   -1.592587   -0.000452
     9          7           0        0.656162   -1.492291    0.000019
    10          7           0        1.524565    0.742115    0.000098
    11          7           0       -2.185427    0.678576   -0.000222
    12          1           0       -3.733717   -0.759091    0.000211
    13          1           0        1.066112    3.005379    0.000067
    14          1           0        3.509447   -0.360560    0.000179
---------------------------------------------------------------------
```

Result for X_2enol6enol(d)_water.log

```
SCF Done:  E(RB3LYP) =  -562.655103003     A.U. after    1 cycles

Zero-point correction=                           0.103347 (Hartree/Particle)
Thermal correction to Energy=                    0.111256
Thermal correction to Enthalpy=                  0.112200
Thermal correction to Gibbs Free Energy=         0.070624
Sum of electronic and zero-point Energies=           -562.551756
Sum of electronic and thermal Energies=              -562.543847
Sum of electronic and thermal Enthalpies=            -562.542903
Sum of electronic and thermal Free Energies=         -562.584479

                    E (Thermal)             CV                S
                     KCal/Mol        Cal/Mol-Kelvin    Cal/Mol-Kelvin
Total                  69.814            31.431            87.503

---------------------------------------------------------------------
```

```
 Center     Atomic      Atomic             Coordinates (Angstroms)
 Number     Number       Type             X           Y           Z
 ---------------------------------------------------------------------
      1          6           0       -0.776234    0.494487    0.000049
      2          6           0       -0.550008   -0.885778    0.000024
      3          6           0        1.639008   -0.622836   -0.000014
      4          6           0        0.371142    1.284099    0.000035
      5          6           0       -2.700778   -0.417633   -0.000022
      6          8           0        0.275417    2.620862   -0.000036
      7          8           0        2.875673   -1.170185    0.000003
      8          7           0       -1.794010   -1.450035   -0.000101
      9          7           0        0.640995   -1.493088   -0.000010
     10          7           0        1.572982    0.716475    0.000075
     11          7           0       -2.138733    0.765965    0.000017
     12          1           0       -3.762200   -0.606499   -0.000128
     13          1           0        1.168287    3.004006   -0.000075
     14          1           0        3.530341   -0.454663   -0.000326
     15          1           0       -2.012567   -2.437513    0.000502
 ---------------------------------------------------------------------
```

Result for X_2enol6keto(a).log

```
 SCF Done:  E(RB3LYP) =  -562.628254204     A.U. after    1 cycles
```

```
 Zero-point correction=                           0.104208 (Hartree/Particle)
 Thermal correction to Energy=                    0.112079
 Thermal correction to Enthalpy=                  0.113024
 Thermal correction to Gibbs Free Energy=         0.071421
 Sum of electronic and zero-point Energies=           -562.524046
 Sum of electronic and thermal Energies=              -562.516175
 Sum of electronic and thermal Enthalpies=            -562.515231
 Sum of electronic and thermal Free Energies=         -562.556833

                     E (Thermal)             CV                S
                      KCal/Mol        Cal/Mol-Kelvin    Cal/Mol-Kelvin
 Total                  70.331             31.182             87.560

 ---------------------------------------------------------------------
 Center     Atomic      Atomic             Coordinates (Angstroms)
 Number     Number       Type             X           Y           Z
 ---------------------------------------------------------------------
      1          6           0        0.828822    0.510525    0.000020
      2          6           0        0.528933   -0.846085   -0.000020
      3          6           0       -1.661353   -0.575640    0.000021
      4          6           0       -0.247846    1.464637   -0.000015
      5          6           0        2.706955   -0.495988    0.000058
      6          8           0       -0.237150    2.675162   -0.000097
      7          8           0       -2.942672   -0.973370    0.000007
      8          7           0        1.737342   -1.483347    0.000020
      9          7           0       -0.694115   -1.440731   -0.000102
     10          7           0       -1.505053    0.769275    0.000115
     11          7           0        2.195824    0.703537    0.000035
     12          1           0        3.759039   -0.733667   -0.000068
```

```
    13          1           0       -2.939035   -1.941588   -0.000011
    14          1           0        1.876530   -2.481377   -0.000194
    15          1           0       -2.329014    1.356463    0.000128
 ---------------------------------------------------------------------
```

Result for X_2enol6keto(a)-N9H.log

```
 SCF Done:  E(RB3LYP) =  -562.085106504     A.U. after    1 cycles


 Zero-point correction=                           0.090815 (Hartree/Particle)
 Thermal correction to Energy=                    0.098479
 Thermal correction to Enthalpy=                  0.099424
 Thermal correction to Gibbs Free Energy=         0.058139
 Sum of electronic and zero-point Energies=           -561.994292
 Sum of electronic and thermal Energies=              -561.986627
 Sum of electronic and thermal Enthalpies=            -561.985683
 Sum of electronic and thermal Free Energies=         -562.026968

                     E (Thermal)             CV                S
                      KCal/Mol        Cal/Mol-Kelvin    Cal/Mol-Kelvin
 Total                   61.797             29.953             86.892

 ---------------------------------------------------------------------
 Center     Atomic      Atomic             Coordinates (Angstroms)
 Number     Number       Type             X           Y           Z
 ---------------------------------------------------------------------
      1          6           0        0.869778    0.474006   -0.000000
      2          6           0        0.571589   -0.913826   -0.000007
      3          6           0       -1.633508   -0.552886    0.000012
      4          6           0       -0.168940    1.443399    0.000016
      5          6           0        2.661653   -0.639403   -0.000011
      6          8           0       -0.157831    2.674084    0.000013
      7          8           0       -2.952860   -0.921358    0.000009
      8          7           0        1.714121   -1.621319   -0.000049
      9          7           0       -0.709438   -1.441795   -0.000004
     10          7           0       -1.449917    0.792100    0.000031
     11          7           0        2.236875    0.625061   -0.000012
     12          1           0        3.717295   -0.884587   -0.000021
     13          1           0       -2.925465   -1.888527    0.000005
     14          1           0       -2.251227    1.405238    0.000014
 ---------------------------------------------------------------------
```

Result for X_2enol6keto(a)-N9H_water.log

```
 SCF Done:  E(RB3LYP) =  -562.193811874     A.U. after    1 cycles


 Zero-point correction=                           0.090694 (Hartree/Particle)
 Thermal correction to Energy=                    0.098412
 Thermal correction to Enthalpy=                  0.099357
```

```
Thermal correction to Gibbs Free Energy=         0.057980
Sum of electronic and zero-point Energies=           -562.103118
Sum of electronic and thermal Energies=              -562.095399
Sum of electronic and thermal Enthalpies=            -562.094455
Sum of electronic and thermal Free Energies=         -562.135831

                   E (Thermal)             CV                S
                    KCal/Mol        Cal/Mol-Kelvin    Cal/Mol-Kelvin
Total                 61.755             29.921            87.084

---------------------------------------------------------------------
Center     Atomic      Atomic             Coordinates (Angstroms)
Number     Number       Type             X           Y           Z
---------------------------------------------------------------------
     1          6           0        0.857566    0.462410   -0.000023
     2          6           0        0.564050   -0.912503   -0.000033
     3          6           0       -1.641425   -0.552387    0.000022
     4          6           0       -0.184880    1.412631   -0.000027
     5          6           0        2.664342   -0.635860    0.000071
     6          8           0       -0.120744    2.659836    0.000049
     7          8           0       -2.949526   -0.889099    0.000043
     8          7           0        1.717549   -1.616742   -0.000105
     9          7           0       -0.704699   -1.443749   -0.000029
    10          7           0       -1.447458    0.794272    0.000042
    11          7           0        2.228998    0.626431   -0.000046
    12          1           0        3.718485   -0.875303    0.000067
    13          1           0       -3.014351   -1.857256    0.000048
    14          1           0       -2.260621    1.399438    0.000064
---------------------------------------------------------------------
```

Result for X_2enol6keto(a)_water.log

```
SCF Done:  E(RB3LYP) =  -562.665150747     A.U. after    1 cycles


Zero-point correction=                           0.103554 (Hartree/Particle)
Thermal correction to Energy=                    0.111540
Thermal correction to Enthalpy=                  0.112485
Thermal correction to Gibbs Free Energy=         0.070661
Sum of electronic and zero-point Energies=           -562.561597
Sum of electronic and thermal Energies=              -562.553610
Sum of electronic and thermal Enthalpies=            -562.552666
Sum of electronic and thermal Free Energies=         -562.594489

                    E (Thermal)             CV                S
                    KCal/Mol        Cal/Mol-Kelvin    Cal/Mol-Kelvin
Total                 69.993             31.294            88.024

---------------------------------------------------------------------
Center     Atomic      Atomic             Coordinates (Angstroms)
Number     Number       Type             X           Y           Z
---------------------------------------------------------------------
     1          6           0        0.816452    0.506125    0.000117
     2          6           0        0.531762   -0.853658   -0.000399
```

3	6	0	-1.660773	-0.577481	-0.000183
4	6	0	-0.260638	1.433784	0.000002
5	6	0	2.700722	-0.491130	0.000293
6	8	0	-0.220461	2.669365	-0.000055
7	8	0	-2.941906	-0.955962	0.000042
8	7	0	1.744098	-1.476635	0.000222
9	7	0	-0.688715	-1.443394	-0.000575
10	7	0	-1.500247	0.771486	0.000381
11	7	0	2.185973	0.714709	-0.000360
12	1	0	3.752139	-0.727822	0.000704
13	1	0	-2.984387	-1.926414	0.001327
14	1	0	1.910920	-2.474583	0.000869
15	1	0	-2.332654	1.352587	0.000549

Result for X_2enol6keto(b).log

```
SCF Done:  E(RB3LYP) =  -562.617347619     A.U. after    1 cycles

Zero-point correction=                           0.103291 (Hartree/Particle)
Thermal correction to Energy=                    0.111609
Thermal correction to Enthalpy=                  0.112553
Thermal correction to Gibbs Free Energy=         0.069905
Sum of electronic and zero-point Energies=          -562.514056
Sum of electronic and thermal Energies=             -562.505739
Sum of electronic and thermal Enthalpies=           -562.504795
Sum of electronic and thermal Free Energies=        -562.547442

                    E (Thermal)             CV                S
                     KCal/Mol        Cal/Mol-Kelvin    Cal/Mol-Kelvin
Total                   70.035             31.920            89.759
```

Center Number	Atomic Number	Atomic Type	Coordinates (Angstroms) X	Y	Z
1	6	0	0.830092	0.520283	0.000096
2	6	0	0.545353	-0.840752	-0.000002
3	6	0	-1.649989	-0.626972	0.000020
4	6	0	-0.259739	1.452594	0.000036
5	6	0	2.719393	-0.462489	-0.000008
6	8	0	-0.283226	2.663557	-0.000118
7	8	0	-2.895546	-1.137757	-0.000135
8	7	0	1.762723	-1.461204	-0.000109
9	7	0	-0.661275	-1.459799	0.000033
10	7	0	-1.508132	0.730518	0.000027
11	7	0	2.194475	0.731465	0.000121
12	1	0	3.774463	-0.686709	-0.000036
13	1	0	-3.565615	-0.444479	0.001161
14	1	0	1.911813	-2.458009	-0.000079
15	1	0	-2.315689	1.339954	-0.000384

Result for X_2enol6keto(b)-N9H.log

```
 SCF Done:  E(RB3LYP) =  -562.072627886     A.U. after    1 cycles

 Zero-point correction=                           0.089979 (Hartree/Particle)
 Thermal correction to Energy=                    0.097964
 Thermal correction to Enthalpy=                  0.098908
 Thermal correction to Gibbs Free Energy=         0.056965
 Sum of electronic and zero-point Energies=           -561.982649
 Sum of electronic and thermal Energies=              -561.974664
 Sum of electronic and thermal Enthalpies=            -561.973720
 Sum of electronic and thermal Free Energies=         -562.015663

                    E (Thermal)             CV                S
                     KCal/Mol        Cal/Mol-Kelvin    Cal/Mol-Kelvin
 Total                 61.473             30.561             88.276

 ---------------------------------------------------------------------
 Center     Atomic      Atomic             Coordinates (Angstroms)
 Number     Number       Type             X           Y           Z
 ---------------------------------------------------------------------
      1          6           0       -0.868594    0.484007    0.017525
      2          6           0       -0.596950   -0.909914   -0.003970
      3          6           0        1.615428   -0.620565   -0.006918
      4          6           0        0.192321    1.426846    0.005358
      5          6           0       -2.681657   -0.590413    0.001207
      6          8           0        0.215681    2.657821   -0.017574
      7          8           0        2.923554   -1.059463   -0.046209
      8          7           0       -1.756693   -1.591381   -0.013382
      9          7           0        0.667153   -1.474121    0.001317
     10          7           0        1.455690    0.741803    0.006937
     11          7           0       -2.229974    0.665901    0.019701
     12          1           0       -3.742444   -0.812277   -0.001657
     13          1           0        3.466460   -0.460838    0.479816
     14          1           0        2.245577    1.351069   -0.149114
 ---------------------------------------------------------------------
```

Result for X_2enol6keto(b)-N9H_water.log

```
 SCF Done:  E(RB3LYP) =  -562.190641971     A.U. after    1 cycles

 Zero-point correction=                           0.090940 (Hartree/Particle)
 Thermal correction to Energy=                    0.098640
 Thermal correction to Enthalpy=                  0.099585
 Thermal correction to Gibbs Free Energy=         0.058241
 Sum of electronic and zero-point Energies=           -562.099702
 Sum of electronic and thermal Energies=              -562.092001
 Sum of electronic and thermal Enthalpies=            -562.091057
 Sum of electronic and thermal Free Energies=         -562.132401
```

	E (Thermal)	CV	S
	KCal/Mol	Cal/Mol-Kelvin	Cal/Mol-Kelvin
Total	61.898	29.845	87.014

Center Number	Atomic Number	Atomic Type	Coordinates (Angstroms) X	Y	Z
1	6	0	0.855236	0.471322	0.000003
2	6	0	0.584566	-0.908369	0.000037
3	6	0	-1.630507	-0.597677	0.000057
4	6	0	-0.204143	1.399634	-0.000021
5	6	0	2.680551	-0.594220	-0.000044
6	8	0	-0.169095	2.647751	-0.000090
7	8	0	-2.900916	-1.065680	-0.000054
8	7	0	1.751531	-1.591623	-0.000013
9	7	0	-0.671222	-1.464531	0.000091
10	7	0	-1.456759	0.755491	0.000049
11	7	0	2.223350	0.660427	-0.000061
12	1	0	3.738857	-0.814720	-0.000091
13	1	0	-3.553932	-0.349482	0.000724
14	1	0	-2.267357	1.365145	-0.000133

Result for X_2enol6keto(b)_water.log

SCF Done: E(RB3LYP) = -562.662205991   A.U. after   1 cycles

```
Zero-point correction=                           0.103627 (Hartree/Particle)
Thermal correction to Energy=                    0.111633
Thermal correction to Enthalpy=                  0.112577
Thermal correction to Gibbs Free Energy=         0.070709
Sum of electronic and zero-point Energies=           -562.558579
Sum of electronic and thermal Energies=              -562.550573
Sum of electronic and thermal Enthalpies=            -562.549629
Sum of electronic and thermal Free Energies=         -562.591497
```

	E (Thermal)	CV	S
	KCal/Mol	Cal/Mol-Kelvin	Cal/Mol-Kelvin
Total	70.051	31.301	88.120

Center Number	Atomic Number	Atomic Type	Coordinates (Angstroms) X	Y	Z
1	6	0	0.814069	0.513758	-0.000193
2	6	0	0.549530	-0.850540	-0.000156
3	6	0	-1.650159	-0.620005	-0.000090
4	6	0	-0.277443	1.420539	-0.000057
5	6	0	2.713482	-0.452763	0.000127
6	8	0	-0.264870	2.656415	-0.000100
7	8	0	-2.888404	-1.127356	0.000451
8	7	0	1.772730	-1.453303	-0.000008

```
    9          7           0        -0.657574   -1.462692   -0.000464
   10          7           0        -1.507659    0.734052    0.000113
   11          7           0         2.180045    0.744690    0.000260
   12          1           0         3.768597   -0.672553   -0.000053
   13          1           0        -3.572227   -0.438652   -0.001165
   14          1           0         1.956187   -2.448261    0.000680
   15          1           0        -2.336027    1.321828    0.000638
 ---------------------------------------------------------------------
```

Result for X_2keto6keto.log

```
 SCF Done:  E(RB3LYP) =  -562.634132576     A.U. after    1 cycles
```

```
 Zero-point correction=                           0.103694 (Hartree/Particle)
 Thermal correction to Energy=                    0.111934
 Thermal correction to Enthalpy=                  0.112878
 Thermal correction to Gibbs Free Energy=         0.070345
 Sum of electronic and zero-point Energies=           -562.530438
 Sum of electronic and thermal Energies=              -562.522199
 Sum of electronic and thermal Enthalpies=            -562.521255
 Sum of electronic and thermal Free Energies=         -562.563787

                     E (Thermal)             CV                S
                      KCal/Mol        Cal/Mol-Kelvin    Cal/Mol-Kelvin
 Total                   70.240             31.777             89.517

 ---------------------------------------------------------------------
 Center     Atomic      Atomic             Coordinates (Angstroms)
 Number     Number       Type             X           Y           Z
 ---------------------------------------------------------------------
      1          6           0         0.793367    0.525287   -0.000280
      2          6           0         0.560723   -0.831894   -0.000038
      3          6           0        -1.821919   -0.609088    0.000112
      4          6           0        -0.322551    1.447273   -0.000055
      5          6           0         2.718887   -0.399687   -0.000163
      6          8           0        -0.308110    2.656352   -0.000113
      7          8           0        -2.936875   -1.086017   -0.000841
      8          7           0         1.782759   -1.432889    0.000859
      9          7           0        -0.679115   -1.408562    0.001319
     10          1           0        -0.838778   -2.403767   -0.002315
     11          7           0        -1.563224    0.748624    0.000529
     12          1           0        -2.387647    1.334928    0.000520
     13          7           0         2.153722    0.769573   -0.000361
     14          1           0         3.778033   -0.601110    0.000038
     15          1           0         1.978222   -2.421305   -0.004481
 ---------------------------------------------------------------------
```

Result for X_2keto6keto-N9H.log

```
 SCF Done:  E(RB3LYP) =  -562.114208736     A.U. after    1 cycles
```

```
Zero-point correction=                           0.091242 (Hartree/Particle)
Thermal correction to Energy=                    0.098959
Thermal correction to Enthalpy=                  0.099903
Thermal correction to Gibbs Free Energy=         0.058313
Sum of electronic and zero-point Energies=           -562.022967
Sum of electronic and thermal Energies=              -562.015250
Sum of electronic and thermal Enthalpies=            -562.014306
Sum of electronic and thermal Free Energies=         -562.055895

                    E (Thermal)             CV                S
                     KCal/Mol        Cal/Mol-Kelvin    Cal/Mol-Kelvin
Total                  62.098             29.731            87.533

---------------------------------------------------------------------
Center     Atomic      Atomic             Coordinates (Angstroms)
Number     Number       Type             X           Y           Z
---------------------------------------------------------------------
     1          6           0        0.844591    0.506451    0.000190
     2          6           0        0.600456   -0.872999    0.000182
     3          6           0       -1.788206   -0.596036    0.000015
     4          6           0       -0.237559    1.432939    0.000055
     5          6           0        2.673861   -0.557979   -0.000356
     6          8           0       -0.249917    2.660092   -0.000362
     7          8           0       -2.941560   -1.030483   -0.000539
     8          7           0        1.739516   -1.568623    0.000439
     9          7           0       -0.682970   -1.400879   -0.000200
    10          1           0       -0.834134   -2.396644   -0.000240
    11          7           0       -1.502426    0.754865    0.000906
    12          1           0       -2.309474    1.360438    0.000880
    13          7           0        2.215009    0.687342   -0.000312
    14          1           0        3.732651   -0.783859    0.000222
---------------------------------------------------------------------
```

Result for X_2keto6keto-N9H_water.log

```
SCF Done:  E(RB3LYP) =  -562.212486934     A.U. after    1 cycles
```

```
Zero-point correction=                           0.091167 (Hartree/Particle)
Thermal correction to Energy=                    0.098783
Thermal correction to Enthalpy=                  0.099728
Thermal correction to Gibbs Free Energy=         0.058426
Sum of electronic and zero-point Energies=           -562.121320
Sum of electronic and thermal Energies=              -562.113703
Sum of electronic and thermal Enthalpies=            -562.112759
Sum of electronic and thermal Free Energies=         -562.154061

                    E (Thermal)             CV                S
                     KCal/Mol        Cal/Mol-Kelvin    Cal/Mol-Kelvin
Total                  61.988             29.526            86.926

---------------------------------------------------------------------
```

```
 Center     Atomic      Atomic             Coordinates (Angstroms)
 Number     Number       Type             X           Y           Z
 ---------------------------------------------------------------------
      1          6           0        0.826986    0.490821   -0.000281
      2          6           0        0.590686   -0.883404   -0.000117
      3          6           0       -1.775145   -0.583295    0.000038
      4          6           0       -0.248962    1.407416   -0.000157
      5          6           0        2.669714   -0.554167    0.000211
      6          8           0       -0.209880    2.650952    0.000121
      7          8           0       -2.938256   -1.011335    0.000105
      8          7           0        1.742997   -1.566650    0.000111
      9          7           0       -0.687306   -1.402288   -0.000289
     10          1           0       -0.853770   -2.400514    0.000442
     11          7           0       -1.503558    0.768258    0.000041
     12          1           0       -2.316805    1.372372    0.000642
     13          7           0        2.197567    0.688906   -0.000106
     14          1           0        3.728096   -0.770600    0.000655
 ---------------------------------------------------------------------
```

Result for X_2keto6keto_water.log

```
 SCF Done:  E(RB3LYP) =  -562.676448442     A.U. after    1 cycles

 Zero-point correction=                           0.103955 (Hartree/Particle)
 Thermal correction to Energy=                    0.111888
 Thermal correction to Enthalpy=                  0.112832
 Thermal correction to Gibbs Free Energy=         0.071003
 Sum of electronic and zero-point Energies=           -562.572493
 Sum of electronic and thermal Energies=              -562.564561
 Sum of electronic and thermal Enthalpies=            -562.563617
 Sum of electronic and thermal Free Energies=         -562.605445

                     E (Thermal)             CV                S
                      KCal/Mol        Cal/Mol-Kelvin    Cal/Mol-Kelvin
 Total                  70.211             31.063             88.036


 ---------------------------------------------------------------------
 Center     Atomic      Atomic             Coordinates (Angstroms)
 Number     Number       Type             X           Y           Z
 ---------------------------------------------------------------------
      1          6           0        0.782027    0.527570   -0.000041
      2          6           0        0.559612   -0.833163   -0.000030
      3          6           0       -1.796000   -0.612270   -0.000023
      4          6           0       -0.328780    1.423571   -0.000030
      5          6           0        2.705137   -0.401825   -0.000134
      6          8           0       -0.304328    2.655595    0.000079
      7          8           0       -2.932892   -1.078396   -0.000188
      8          7           0        1.777189   -1.424257    0.000305
      9          7           0       -0.675235   -1.407105    0.000289
     10          1           0       -0.808370   -2.411463    0.000019
     11          7           0       -1.558059    0.746966   -0.000031
     12          1           0       -2.389874    1.327524    0.000315
```

```
    13          7           0        2.145708    0.779819   -0.000089
    14          1           0        3.762656   -0.607577   -0.000166
    15          1           0        1.974144   -2.417337   -0.001071
 ---------------------------------------------------------------------
```

Result for X_N3anion.log

```
 SCF Done:  E(RB3LYP) =  -562.104927410     A.U. after    1 cycles


 Zero-point correction=                           0.090637 (Hartree/Particle)
 Thermal correction to Energy=                    0.098403
 Thermal correction to Enthalpy=                  0.099347
 Thermal correction to Gibbs Free Energy=         0.057781
 Sum of electronic and zero-point Energies=          -562.014290
 Sum of electronic and thermal Energies=             -562.006525
 Sum of electronic and thermal Enthalpies=           -562.005581
 Sum of electronic and thermal Free Energies=        -562.047146

                     E (Thermal)             CV                S
                      KCal/Mol        Cal/Mol-Kelvin    Cal/Mol-Kelvin
 Total                   61.749             30.066             87.482

 ---------------------------------------------------------------------
 Center     Atomic      Atomic             Coordinates (Angstroms)
 Number     Number       Type             X           Y           Z
 ---------------------------------------------------------------------
      1          6           0        0.767482    0.532838   -0.000050
      2          6           0        0.506459   -0.844491    0.000091
      3          6           0       -1.748215   -0.725045    0.000133
      4          6           0       -0.356909    1.421870   -0.000175
      5          6           0        2.693474   -0.397216    0.000028
      6          8           0       -0.381593    2.654240   -0.000216
      7          8           0       -2.915579   -1.130793    0.000076
      8          7           0        1.760652   -1.423887   -0.000003
      9          7           0       -0.643988   -1.516521    0.000143
     10          7           0       -1.548445    0.683729   -0.000011
     11          1           0       -2.398272    1.228748   -0.000045
     12          7           0        2.134191    0.780028    0.000010
     13          1           0        3.755532   -0.591927    0.000028
     14          1           0        1.929498   -2.415575    0.000007
 ---------------------------------------------------------------------
```

Result for X_N3anion-N9H.log

```
 SCF Done:  E(RB3LYP) =  -561.418558299     A.U. after    1 cycles


 Zero-point correction=                           0.077662 (Hartree/Particle)
 Thermal correction to Energy=                    0.084998
 Thermal correction to Enthalpy=                  0.085942
```

```
Thermal correction to Gibbs Free Energy=        0.045114
Sum of electronic and zero-point Energies=          -561.340896
Sum of electronic and thermal Energies=             -561.333560
Sum of electronic and thermal Enthalpies=           -561.332616
Sum of electronic and thermal Free Energies=        -561.373444

                    E (Thermal)             CV                S
                     KCal/Mol        Cal/Mol-Kelvin    Cal/Mol-Kelvin
Total                  53.337             28.230             85.930

---------------------------------------------------------------------
Center     Atomic      Atomic             Coordinates (Angstroms)
Number     Number       Type             X           Y           Z
---------------------------------------------------------------------
     1          6           0       -0.810556    0.492424    0.000024
     2          6           0       -0.557284   -0.908676   -0.000095
     3          6           0        1.724724   -0.706669    0.000072
     4          6           0        0.282270    1.396536    0.000084
     5          6           0       -2.657892   -0.534302    0.000156
     6          8           0        0.307059    2.651151    0.000143
     7          8           0        2.937918   -1.061820   -0.000122
     8          7           0       -1.759770   -1.559306   -0.000144
     9          7           0        0.666817   -1.519279   -0.000097
    10          7           0        1.497696    0.697313    0.000059
    11          1           0        2.330035    1.266622    0.000097
    12          7           0       -2.183167    0.711971   -0.000087
    13          1           0       -3.728454   -0.732030    0.000171
---------------------------------------------------------------------
```

Result for X_N3anion-N9H_water.log

```
SCF Done:  E(RB3LYP) =  -561.732524788     A.U. after    1 cycles


Zero-point correction=                          0.078378 (Hartree/Particle)
Thermal correction to Energy=                   0.085643
Thermal correction to Enthalpy=                 0.086587
Thermal correction to Gibbs Free Energy=        0.045912
Sum of electronic and zero-point Energies=          -561.654147
Sum of electronic and thermal Energies=             -561.646882
Sum of electronic and thermal Enthalpies=           -561.645938
Sum of electronic and thermal Free Energies=        -561.686613

                    E (Thermal)             CV                S
                     KCal/Mol        Cal/Mol-Kelvin    Cal/Mol-Kelvin
Total                  53.742             27.927             85.608

---------------------------------------------------------------------
Center     Atomic      Atomic             Coordinates (Angstroms)
Number     Number       Type             X           Y           Z
---------------------------------------------------------------------
     1          6           0       -0.799397    0.478998    0.000078
     2          6           0       -0.552441   -0.906627    0.000005
     3          6           0        1.717429   -0.674340    0.000198
```

4	6	0	0.284521	1.375666	0.000123
5	6	0	-2.653092	-0.541483	0.000040
6	8	0	0.262002	2.637206	0.000059
7	8	0	2.929911	-1.061554	-0.000333
8	7	0	-1.747034	-1.561034	-0.000171
9	7	0	0.675199	-1.502796	-0.000018
10	7	0	1.507555	0.712803	0.000078
11	1	0	2.338145	1.290761	-0.000021
12	7	0	-2.169834	0.701030	0.000051
13	1	0	-3.716778	-0.739283	-0.000013

Result for X_N3anion_water.log

```
SCF Done:  E(RB3LYP) =  -562.214494360     A.U. after    1 cycles

Zero-point correction=                           0.091207 (Hartree/Particle)
Thermal correction to Energy=                    0.098731
Thermal correction to Enthalpy=                  0.099675
Thermal correction to Gibbs Free Energy=         0.058614
Sum of electronic and zero-point Energies=           -562.123287
Sum of electronic and thermal Energies=              -562.115764
Sum of electronic and thermal Enthalpies=            -562.114819
Sum of electronic and thermal Free Energies=         -562.155880
```

	E (Thermal) KCal/Mol	CV Cal/Mol-Kelvin	S Cal/Mol-Kelvin
Total	61.954	29.392	86.420

Center Number	Atomic Number	Atomic Type	Coordinates (Angstroms) X	Y	Z
1	6	0	0.755173	0.522820	-0.000122
2	6	0	0.518233	-0.851516	0.000099
3	6	0	-1.732998	-0.703301	0.000356
4	6	0	-0.360515	1.394463	-0.000528
5	6	0	2.683876	-0.393992	0.000081
6	8	0	-0.363773	2.644245	-0.000211
7	8	0	-2.914940	-1.133902	0.000080
8	7	0	1.766828	-1.419270	-0.000088
9	7	0	-0.653523	-1.505720	0.000147
10	7	0	-1.558373	0.688534	-0.000039
11	1	0	-2.407496	1.240650	0.000007
12	7	0	2.121313	0.788056	0.000238
13	1	0	3.744358	-0.587487	0.000108
14	1	0	1.976525	-2.407947	-0.000182

Result for 2OA+N7H.log

```
SCF Done:  E(RB3LYP) =  -543.103687162     A.U. after    1 cycles


Zero-point correction=                           0.128064 (Hartree/Particle)
Thermal correction to Energy=                    0.137152
Thermal correction to Enthalpy=                  0.138096
Thermal correction to Gibbs Free Energy=         0.093968
Sum of electronic and zero-point Energies=           -542.975623
Sum of electronic and thermal Energies=              -542.966535
Sum of electronic and thermal Enthalpies=            -542.965591
Sum of electronic and thermal Free Energies=         -543.009719

                    E (Thermal)             CV                S
                     KCal/Mol        Cal/Mol-Kelvin    Cal/Mol-Kelvin
Total                   86.064             35.150             92.875

---------------------------------------------------------------------
Center     Atomic      Atomic             Coordinates (Angstroms)
Number     Number       Type             X           Y           Z
---------------------------------------------------------------------
     1          6           0        0.474564   -0.910293   -0.000036
     2          6           0        0.695986    0.482923   -0.000213
     3          6           0       -0.427240    1.315261    0.000026
     4          6           0       -1.779926   -0.820577   -0.000229
     5          6           0        2.682699   -0.506790   -0.000150
     6          7           0       -1.589119    0.637998    0.000037
     7          7           0       -0.632021   -1.576588    0.000078
     8          7           0       -0.417628    2.655606   -0.000038
     9          7           0        1.761786   -1.473843    0.000187
    10          7           0        2.081047    0.675829   -0.000118
    11          1           0        1.946591   -2.469586    0.000432
    12          1           0        3.750111   -0.656425   -0.000254
    13          8           0       -2.909878   -1.215550    0.000092
    14          1           0       -1.276734    3.183624    0.000910
    15          1           0        0.438539    3.182929    0.001258
    16          1           0       -2.471085    1.137638    0.000076
    17          1           0        2.586647    1.550066   -0.000557
---------------------------------------------------------------------
```

Result for 2OA+N7H_water.log

```
SCF Done:  E(RB3LYP) =  -543.242081216     A.U. after    1 cycles


Zero-point correction=                           0.128833 (Hartree/Particle)
Thermal correction to Energy=                    0.137391
Thermal correction to Enthalpy=                  0.138335
Thermal correction to Gibbs Free Energy=         0.095496
Sum of electronic and zero-point Energies=           -543.113248
Sum of electronic and thermal Energies=              -543.104691
Sum of electronic and thermal Enthalpies=            -543.103746
Sum of electronic and thermal Free Energies=         -543.146585
```

	E (Thermal) KCal/Mol	CV Cal/Mol-Kelvin	S Cal/Mol-Kelvin
Total	86.214	33.996	90.162

Center Number	Atomic Number	Atomic Type	Coordinates (Angstroms) X	Y	Z
1	6	0	0.486522	-0.913240	-0.000034
2	6	0	0.699036	0.462413	-0.000156
3	6	0	-0.414457	1.305808	-0.000004
4	6	0	-1.761617	-0.772061	0.000041
5	6	0	2.675116	-0.506576	-0.000095
6	7	0	-1.591925	0.634998	0.000048
7	7	0	-0.663878	-1.564333	0.000049
8	7	0	-0.390474	2.628519	-0.000090
9	7	0	1.752222	-1.478311	0.000034
10	7	0	2.072242	0.668647	-0.000122
11	1	0	1.961699	-2.470463	0.000123
12	1	0	3.740623	-0.661749	-0.000122
13	8	0	-2.924552	-1.196571	0.000050
14	1	0	-1.248866	3.163106	0.000846
15	1	0	0.489665	3.124181	0.000919
16	1	0	-2.454602	1.170606	0.000141
17	1	0	2.552981	1.562175	-0.000245

Result for 8BA+N7H.log

```
SCF Done:  E(RB3LYP) =  -3041.38401768     A.U. after    7 cycles

Zero-point correction=                           0.113814 (Hartree/Particle)
Thermal correction to Energy=                    0.123057
Thermal correction to Enthalpy=                  0.124001
Thermal correction to Gibbs Free Energy=         0.078010
Sum of electronic and zero-point Energies=          -3041.270204
Sum of electronic and thermal Energies=             -3041.260960
Sum of electronic and thermal Enthalpies=           -3041.260016
Sum of electronic and thermal Free Energies=        -3041.306008
```

	E (Thermal) KCal/Mol	CV Cal/Mol-Kelvin	S Cal/Mol-Kelvin
Total	77.220	34.495	96.797

Center Number	Atomic Number	Atomic Type	Coordinates (Angstroms) X	Y	Z
1	6	0	-0.972291	-0.999829	-0.000193
2	6	0	-1.121017	0.382888	0.000018
3	6	0	-2.441211	0.884987	0.000043
4	6	0	-3.145082	-1.325183	-0.000336

```
     5         6           0        1.059356   -0.080115    0.000031
     6         7           0       -3.424665   -0.022785   -0.000142
     7         7           0       -1.934203   -1.897641   -0.000375
     8         7           0       -2.776233    2.184408    0.000227
     9         7           0        0.402651   -1.245114   -0.000177
    10         7           0        0.172376    0.918776    0.000152
    11         1           0       -2.107192    2.934019    0.000423
    12         1           0       -3.758853    2.416493    0.000243
    13         1           0       -3.995567   -1.996102   -0.000478
    14        35           0        2.892210    0.119283    0.000132
    15         1           0        0.833584   -2.161741   -0.000303
    16         1           0        0.442678    1.892443    0.000310
 ---------------------------------------------------------------------
```

Result for 8BA+N7H_water.log

```
 SCF Done:  E(RB3LYP) =  -3041.48896554     A.U. after    1 cycles


 Zero-point correction=                           0.114029 (Hartree/Particle)
 Thermal correction to Energy=                    0.123112
 Thermal correction to Enthalpy=                  0.124057
 Thermal correction to Gibbs Free Energy=         0.078620
 Sum of electronic and zero-point Energies=          -3041.374936
 Sum of electronic and thermal Energies=             -3041.365853
 Sum of electronic and thermal Enthalpies=           -3041.364909
 Sum of electronic and thermal Free Energies=        -3041.410345

                     E (Thermal)             CV                S
                      KCal/Mol        Cal/Mol-Kelvin    Cal/Mol-Kelvin
 Total                   77.254             34.258             95.629

 ---------------------------------------------------------------------
 Center     Atomic      Atomic             Coordinates (Angstroms)
 Number     Number       Type             X           Y           Z
 ---------------------------------------------------------------------
     1         6           0        0.970196   -1.006347    0.000186
     2         6           0        1.116684    0.369916   -0.000019
     3         6           0        2.425585    0.894573   -0.000041
     4         6           0        3.151510   -1.307026    0.000332
     5         6           0       -1.042974   -0.084464   -0.000034
     6         7           0        3.430757   -0.002513    0.000147
     7         7           0        1.953383   -1.899265    0.000367
     8         7           0        2.707289    2.196794   -0.000223
     9         7           0       -0.395043   -1.255633    0.000168
    10         7           0       -0.163250    0.912676   -0.000164
    11         1           0        1.977472    2.892957   -0.000422
    12         1           0        3.671925    2.494719   -0.000251
    13         1           0        4.007657   -1.969582    0.000473
    14        35           0       -2.882835    0.114079   -0.000125
    15         1           0       -0.821524   -2.177468    0.000305
    16         1           0       -0.394273    1.902304   -0.000336
 ---------------------------------------------------------------------
```

Result for 8BG+N7H.log

```
SCF Done:  E(RB3LYP) =  -3116.66698988     A.U. after    7 cycles

Zero-point correction=                           0.119004 (Hartree/Particle)
Thermal correction to Energy=                    0.129153
Thermal correction to Enthalpy=                  0.130097
Thermal correction to Gibbs Free Energy=         0.082317
Sum of electronic and zero-point Energies=          -3116.547986
Sum of electronic and thermal Energies=             -3116.537837
Sum of electronic and thermal Enthalpies=           -3116.536892
Sum of electronic and thermal Free Energies=        -3116.584673

                    E (Thermal)             CV                S
                     KCal/Mol        Cal/Mol-Kelvin    Cal/Mol-Kelvin
Total                   81.045             38.362            100.563

---------------------------------------------------------------------
Center     Atomic      Atomic             Coordinates (Angstroms)
Number     Number       Type             X           Y           Z
---------------------------------------------------------------------
     1          6           0       -0.712121    0.669879    0.000000
     2          6           0       -0.770073   -0.706970    0.000107
     3          6           0       -2.988731   -0.794633    0.000268
     4          6           0       -1.913218    1.453013    0.000025
     5          8           0       -2.028744    2.654526   -0.000057
     6          7           0        0.552228   -1.140509    0.000047
     7          7           0       -1.852339   -1.479105    0.000241
     8          7           0       -3.033698    0.578425    0.000168
     9          1           0       -3.926669    1.057909    0.000192
    10          7           0        0.625853    1.031284   -0.000120
    11          7           0       -4.147194   -1.463887    0.000396
    12          1           0       -4.113430   -2.471279    0.000476
    13          1           0       -5.044272   -1.008150    0.000439
    14          6           0        1.372433   -0.069527   -0.000090
    15          1           0        0.857968   -2.105354    0.000098
    16         35           0        3.215148   -0.133795   -0.000215
    17          1           0        0.982500    1.979467   -0.000216
---------------------------------------------------------------------
```

Result for 8BG+N7H_water.log

```
SCF Done:  E(RB3LYP) =  -3116.77076801     A.U. after    1 cycles

Zero-point correction=                           0.118366 (Hartree/Particle)
Thermal correction to Energy=                    0.128675
Thermal correction to Enthalpy=                  0.129619
Thermal correction to Gibbs Free Energy=         0.081041
Sum of electronic and zero-point Energies=          -3116.652402
Sum of electronic and thermal Energies=             -3116.642094
```

```
Sum of electronic and thermal Enthalpies=          -3116.641149
Sum of electronic and thermal Free Energies=       -3116.689727

                    E (Thermal)            CV                S
                     KCal/Mol        Cal/Mol-Kelvin    Cal/Mol-Kelvin
Total                  80.744            38.294            102.241
```

Center Number	Atomic Number	Atomic Type	Coordinates (Angstroms) X	Y	Z
1	6	0	0.710347	0.665870	-0.000220
2	6	0	0.761267	-0.713870	-0.000040
3	6	0	2.980512	-0.798618	-0.000265
4	6	0	1.912537	1.427351	-0.000001
5	8	0	2.036956	2.649564	0.000319
6	7	0	-0.555796	-1.143887	0.000083
7	7	0	1.843455	-1.488752	0.000313
8	7	0	3.019241	0.574651	0.000130
9	1	0	3.925866	1.033510	0.000064
10	7	0	-0.624728	1.032582	-0.000148
11	7	0	4.147180	-1.440893	-0.006368
12	1	0	4.148693	-2.449132	0.018802
13	1	0	5.025109	-0.944046	0.021423
14	6	0	-1.362579	-0.068205	-0.000007
15	1	0	-0.860825	-2.112131	0.000088
16	35	0	-3.210781	-0.129184	0.000068
17	1	0	-0.975125	1.985636	-0.000192

Result for 8OA+N7H.log

```
SCF Done:  E(RB3LYP) =  -543.080209089     A.U. after    1 cycles

Zero-point correction=                           0.128631 (Hartree/Particle)
Thermal correction to Energy=                    0.137484
Thermal correction to Enthalpy=                  0.138428
Thermal correction to Gibbs Free Energy=         0.094759
Sum of electronic and zero-point Energies=           -542.951578
Sum of electronic and thermal Energies=              -542.942725
Sum of electronic and thermal Enthalpies=            -542.941781
Sum of electronic and thermal Free Energies=         -542.985450

                    E (Thermal)            CV                S
                     KCal/Mol        Cal/Mol-Kelvin    Cal/Mol-Kelvin
Total                  86.272            34.297             91.908
```

Center Number	Atomic Number	Atomic Type	Coordinates (Angstroms) X	Y	Z
1	6	0	0.174744	-0.926243	-0.000143
2	6	0	-0.160472	0.416219	-0.000204

```
     3          6           0        -1.516156    0.757902    0.000015
     4          6           0        -1.957224   -1.507810    0.000075
     5          6           0         2.253653    0.079330    0.000051
     6          7           0        -2.399304   -0.265167    0.000147
     7          7           0        -0.669854   -1.921791   -0.000018
     8          7           0        -2.017695    2.001324    0.000053
     9          7           0         1.570921   -1.076537   -0.000054
    10          7           0         1.072065    1.200194   -0.000380
    11          1           0        -1.455621    2.834707    0.000410
    12          1           0        -3.022830    2.100638    0.000619
    13          1           0         2.039153   -1.975686   -0.000305
    14          1           0        -2.707700   -2.289475    0.000155
    15          8           0         3.381272    0.409366    0.000450
    16          1           0         1.218315    1.785675   -0.830200
    17          1           0         1.218302    1.786673    0.828721
 ---------------------------------------------------------------------
```

Result for 8OA+N7H_water.log

```
 SCF Done:  E(RB3LYP) =  -543.207121109     A.U. after    7 cycles

 Zero-point correction=                           0.128557 (Hartree/Particle)
 Thermal correction to Energy=                    0.137433
 Thermal correction to Enthalpy=                  0.138378
 Thermal correction to Gibbs Free Energy=         0.094249
 Sum of electronic and zero-point Energies=           -543.078564
 Sum of electronic and thermal Energies=              -543.069688
 Sum of electronic and thermal Enthalpies=            -543.068744
 Sum of electronic and thermal Free Energies=         -543.112872

                     E (Thermal)             CV                S
                      KCal/Mol        Cal/Mol-Kelvin    Cal/Mol-Kelvin
 Total                   86.241             33.836             92.875

 ---------------------------------------------------------------------
 Center     Atomic      Atomic             Coordinates (Angstroms)
 Number     Number       Type             X           Y           Z
 ---------------------------------------------------------------------
     1          6           0         0.166265   -0.940819    0.000019
     2          6           0        -0.152359    0.395485   -0.000050
     3          6           0        -1.492159    0.775370   -0.000019
     4          6           0        -1.978153   -1.482372   -0.000025
     5          6           0         2.202146    0.089603    0.000007
     6          7           0        -2.407077   -0.231781   -0.000045
     7          7           0        -0.707367   -1.930565    0.000042
     8          7           0        -1.930618    2.035517   -0.000144
     9          7           0         1.552985   -1.089339    0.000071
    10          7           0         1.087700    1.159355   -0.000116
    11          1           0        -1.293616    2.817979    0.001090
    12          1           0        -2.924419    2.211394    0.000746
    13          1           0         2.043414   -1.977679    0.000143
    14          1           0        -2.744320   -2.247789   -0.000046
    15          8           0         3.357402    0.382142    0.000023
```

16	1	0	1.207968	1.756418	-0.831355
17	1	0	1.207962	1.756611	0.830985

Result for 8OG+N7H.log

```
SCF Done:  E(RB3LYP) =  -618.365507933     A.U. after    8 cycles
```

```
Zero-point correction=                           0.133922 (Hartree/Particle)
Thermal correction to Energy=                    0.143687
Thermal correction to Enthalpy=                  0.144632
Thermal correction to Gibbs Free Energy=         0.098843
Sum of electronic and zero-point Energies=           -618.231586
Sum of electronic and thermal Energies=              -618.221820
Sum of electronic and thermal Enthalpies=            -618.220876
Sum of electronic and thermal Free Energies=         -618.266665
```

	E (Thermal) KCal/Mol	CV Cal/Mol-Kelvin	S Cal/Mol-Kelvin
Total	90.165	38.023	96.370

Center Number	Atomic Number	Atomic Type	Coordinates (Angstroms) X	Y	Z
1	6	0	0.339547	0.613063	-0.000001
2	6	0	0.219424	-0.749509	-0.000000
3	6	0	-2.010152	-0.701074	0.000000
4	6	0	-0.796611	1.462903	-0.000000
5	8	0	-0.842756	2.671108	0.000000
6	7	0	1.491861	-1.333192	-0.000000
7	7	0	-0.899293	-1.448189	-0.000000
8	7	0	-1.982148	0.661411	0.000000
9	7	0	1.743639	0.976149	-0.000000
10	7	0	-3.187113	-1.326653	0.000000
11	6	0	2.500349	-0.430328	0.000000
12	8	0	3.675095	-0.494467	0.000000
13	1	0	-4.068136	-0.838950	-0.000001
14	1	0	-3.189194	-2.335306	-0.000000
15	1	0	2.045612	1.509174	0.824863
16	1	0	1.666796	-2.330726	0.000000
17	1	0	-2.843367	1.196493	0.000001
18	1	0	2.045612	1.509174	-0.824863

Result for 8OG+N7H_water.log

```
SCF Done:  E(RB3LYP) =  -618.489683735     A.U. after    1 cycles
```

```
Zero-point correction=                           0.133192 (Hartree/Particle)
Thermal correction to Energy=                    0.142971
Thermal correction to Enthalpy=                  0.143916
Thermal correction to Gibbs Free Energy=         0.098095
Sum of electronic and zero-point Energies=          -618.356492
Sum of electronic and thermal Energies=             -618.346712
Sum of electronic and thermal Enthalpies=           -618.345768
Sum of electronic and thermal Free Energies=        -618.391589

                    E (Thermal)             CV                S
                     KCal/Mol        Cal/Mol-Kelvin    Cal/Mol-Kelvin
Total                  89.716             37.822            96.438
```

Center Number	Atomic Number	Atomic Type	Coordinates (Angstroms) X	Y	Z
1	6	0	0.341551	0.609088	-0.000004
2	6	0	0.224691	-0.756688	-0.000003
3	6	0	-2.003240	-0.706818	-0.000000
4	6	0	-0.794528	1.435352	-0.000009
5	8	0	-0.856001	2.667443	0.000003
6	7	0	1.496446	-1.324619	-0.000001
7	7	0	-0.893990	-1.459964	-0.000003
8	7	0	-1.966719	0.655546	-0.000001
9	7	0	1.749473	0.958477	0.000002
10	7	0	-3.189708	-1.302593	0.000004
11	6	0	2.477951	-0.399316	0.000002
12	8	0	3.666425	-0.483160	0.000006
13	1	0	-4.048132	-0.770798	0.000006
14	1	0	-3.231333	-2.310637	0.000004
15	1	0	2.049545	1.489222	0.830025
16	1	0	1.691896	-2.320314	-0.000000
17	1	0	-2.841992	1.171406	0.000003
18	1	0	2.049550	1.489224	-0.830017

Result for A+N7H.log

```
SCF Done:  E(RB3LYP) =  -467.849942950     A.U. after    2 cycles

Zero-point correction=                           0.124308 (Hartree/Particle)
Thermal correction to Energy=                    0.132018
Thermal correction to Enthalpy=                  0.132962
Thermal correction to Gibbs Free Energy=         0.091919
Sum of electronic and zero-point Energies=          -467.725635
Sum of electronic and thermal Energies=             -467.717925
Sum of electronic and thermal Enthalpies=           -467.716981
Sum of electronic and thermal Free Energies=        -467.758024

                    E (Thermal)             CV                S
                     KCal/Mol        Cal/Mol-Kelvin    Cal/Mol-Kelvin
Total                  82.842             30.161            86.382
```

Center Number	Atomic Number	Atomic Type	Coordinates (Angstroms) X	Y	Z
1	6	0	-0.671157	0.786409	-0.000111
2	6	0	-0.142431	-0.501481	-0.000307
3	6	0	1.267708	-0.614595	-0.000104
4	6	0	1.324897	1.704442	0.000357
5	6	0	-2.360279	-0.662653	0.000035
6	7	0	1.957605	0.530824	0.000123
7	7	0	0.003837	1.917217	0.000090
8	7	0	1.951220	-1.768508	0.000026
9	7	0	-2.057530	0.635606	0.000010
10	7	0	-1.233736	-1.372988	-0.000014
11	1	0	1.517872	-2.675109	-0.000738
12	1	0	2.959741	-1.717960	-0.000014
13	1	0	-2.723816	1.398697	0.000299
14	1	0	1.955564	2.585222	0.000075
15	1	0	-3.355975	-1.075274	0.000088
16	1	0	-1.215581	-2.383363	-0.000577

Result for A+N7H_water.log

```
SCF Done:  E(RB3LYP) =  -467.965678055     A.U. after    1 cycles


Zero-point correction=                           0.124429 (Hartree/Particle)
Thermal correction to Energy=                    0.132073
Thermal correction to Enthalpy=                  0.133017
Thermal correction to Gibbs Free Energy=         0.092158
Sum of electronic and zero-point Energies=          -467.841249
Sum of electronic and thermal Energies=             -467.833605
Sum of electronic and thermal Enthalpies=           -467.832661
Sum of electronic and thermal Free Energies=        -467.873520
```

	E (Thermal) KCal/Mol	CV Cal/Mol-Kelvin	S Cal/Mol-Kelvin
Total	82.877	30.000	85.995

Center Number	Atomic Number	Atomic Type	Coordinates (Angstroms) X	Y	Z
1	6	0	-0.669595	0.799312	0.000004
2	6	0	-0.145460	-0.482699	-0.000184
3	6	0	1.254923	-0.624986	-0.000027
4	6	0	1.348030	1.690188	-0.000083
5	6	0	-2.345812	-0.647273	0.000044
6	7	0	1.977546	0.511964	0.000012
7	7	0	0.034740	1.929704	0.000111
8	7	0	1.881320	-1.803925	0.000085
9	7	0	-2.046613	0.653918	0.000149

```
    10          7           0        -1.224589   -1.355222   -0.000122
    11          1           0         1.369074   -2.673209   -0.000340
    12          1           0         2.890205   -1.828858   -0.000043
    13          1           0        -2.729874    1.403258    0.000475
    14          1           0         1.989599    2.562400    0.000382
    15          1           0        -3.341487   -1.057363    0.000072
    16          1           0        -1.186870   -2.368555   -0.000709
 ---------------------------------------------------------------------
```

Result for G+N7H.log

```
 SCF Done:  E(RB3LYP) =  -543.133581799     A.U. after    1 cycles

 Zero-point correction=                           0.129452 (Hartree/Particle)
 Thermal correction to Energy=                    0.138068
 Thermal correction to Enthalpy=                  0.139012
 Thermal correction to Gibbs Free Energy=         0.096042
 Sum of electronic and zero-point Energies=           -543.004130
 Sum of electronic and thermal Energies=              -542.995514
 Sum of electronic and thermal Enthalpies=            -542.994570
 Sum of electronic and thermal Free Energies=         -543.037540

                     E (Thermal)             CV                S
                      KCal/Mol        Cal/Mol-Kelvin    Cal/Mol-Kelvin
 Total                   86.639             34.059             90.438

 ---------------------------------------------------------------------
 Center     Atomic      Atomic             Coordinates (Angstroms)
 Number     Number       Type             X           Y           Z
 ---------------------------------------------------------------------
      1          6           0        -0.784729    0.474500   -0.000046
      2          6           0        -0.472211   -0.869339   -0.000001
      3          6           0         1.724438   -0.542805    0.000021
      4          6           0         0.253191    1.467398   -0.000025
      5          8           0         0.141868    2.668963   -0.000017
      6          7           0        -1.691101   -1.536295    0.000023
      7          7           0         0.736151   -1.426203    0.000034
      8          7           0         1.514422    0.816463   -0.000005
      9          7           0        -2.162369    0.579757   -0.000029
     10          7           0         2.987529   -0.983470   -0.000012
     11          6           0        -2.691399   -0.637356   -0.000003
     12          1           0        -3.743286   -0.868383   -0.000003
     13          1           0        -1.806960   -2.541864    0.000053
     14          1           0         3.783775   -0.368285    0.000243
     15          1           0         3.142851   -1.979458    0.000156
     16          1           0         2.304176    1.451729   -0.000013
     17          1           0        -2.683657    1.448407   -0.000059
 ---------------------------------------------------------------------
```

Result for G+N7H_water.log

```
SCF Done:  E(RB3LYP) =  -543.246927923     A.U. after    1 cycles

Zero-point correction=                           0.128814 (Hartree/Particle)
Thermal correction to Energy=                    0.137521
Thermal correction to Enthalpy=                  0.138465
Thermal correction to Gibbs Free Energy=         0.095189
Sum of electronic and zero-point Energies=           -543.118114
Sum of electronic and thermal Energies=              -543.109407
Sum of electronic and thermal Enthalpies=            -543.108463
Sum of electronic and thermal Free Energies=         -543.151738

                    E (Thermal)             CV                S
                     KCal/Mol        Cal/Mol-Kelvin    Cal/Mol-Kelvin
Total                   86.296             34.102            91.082
```

Center Number	Atomic Number	Atomic Type	Coordinates (Angstroms) X	Y	Z
1	6	0	-0.780551	0.480415	-0.001099
2	6	0	-0.485801	-0.869794	-0.000081
3	6	0	1.716166	-0.559319	-0.000715
4	6	0	0.267616	1.439438	-0.000006
5	8	0	0.177553	2.667236	0.001404
6	7	0	-1.703767	-1.522303	0.000987
7	7	0	0.722432	-1.438967	0.001272
8	7	0	1.509445	0.799041	0.000094
9	7	0	-2.159806	0.598000	-0.000570
10	7	0	2.983319	-0.980412	-0.018641
11	6	0	-2.690466	-0.613414	0.000518
12	1	0	-3.743261	-0.838225	0.000828
13	1	0	-1.846562	-2.526337	0.001521
14	1	0	3.751423	-0.331017	0.060884
15	1	0	3.166875	-1.969585	0.052883
16	1	0	2.318444	1.413347	0.000114
17	1	0	-2.690485	1.462466	-0.001163

Result for H+N7H.log

```
SCF Done:  E(RB3LYP) =  -487.728652152     A.U. after    1 cycles

Zero-point correction=                           0.113396 (Hartree/Particle)
Thermal correction to Energy=                    0.120257
Thermal correction to Enthalpy=                  0.121201
Thermal correction to Gibbs Free Energy=         0.081856
Sum of electronic and zero-point Energies=           -487.615256
Sum of electronic and thermal Energies=              -487.608395
Sum of electronic and thermal Enthalpies=            -487.607451
Sum of electronic and thermal Free Energies=         -487.646796
```

```
                   E (Thermal)             CV                 S
                    KCal/Mol        Cal/Mol-Kelvin    Cal/Mol-Kelvin
 Total                 75.463             27.433             82.809

 ---------------------------------------------------------------------
 Center     Atomic      Atomic             Coordinates (Angstroms)
 Number     Number       Type             X           Y           Z
 ---------------------------------------------------------------------
      1          6           0       -0.209551    0.513854    0.000012
      2          6           0       -0.574879   -0.812618    0.000010
      3          6           0        1.505271   -1.547542   -0.000065
      4          6           0        1.178565    0.907012   -0.000029
      5          8           0        1.636806    2.020873   -0.000031
      6          7           0       -1.962063   -0.827576    0.000057
      7          7           0        0.243055   -1.878424   -0.000027
      8          7           0        1.973178   -0.271211   -0.000066
      9          7           0       -1.371376    1.258607    0.000059
     10          1           0        2.255469   -2.329580   -0.000096
     11          6           0       -2.415182    0.433593    0.000077
     12          1           0       -3.451509    0.729089    0.000110
     13          1           0       -2.538940   -1.660856    0.000070
     14          1           0        2.975864   -0.112618   -0.000096
     15          1           0       -1.420239    2.271412    0.000072
 ---------------------------------------------------------------------
```

Result for H+N7H_water.log

```
 SCF Done:  E(RB3LYP) =  -487.843368252     A.U. after    1 cycles

 Zero-point correction=                           0.113275 (Hartree/Particle)
 Thermal correction to Energy=                    0.120121
 Thermal correction to Enthalpy=                  0.121065
 Thermal correction to Gibbs Free Energy=         0.081781
 Sum of electronic and zero-point Energies=           -487.730093
 Sum of electronic and thermal Energies=              -487.723247
 Sum of electronic and thermal Enthalpies=            -487.722303
 Sum of electronic and thermal Free Energies=         -487.761587

                   E (Thermal)             CV                 S
                    KCal/Mol        Cal/Mol-Kelvin    Cal/Mol-Kelvin
 Total                 75.377             27.437             82.680

 ---------------------------------------------------------------------
 Center     Atomic      Atomic             Coordinates (Angstroms)
 Number     Number       Type             X           Y           Z
 ---------------------------------------------------------------------
      1          6           0       -0.207877    0.515276    0.000017
      2          6           0       -0.583075   -0.811203    0.000014
      3          6           0        1.501256   -1.543803   -0.000065
      4          6           0        1.171069    0.885679   -0.000028
      5          8           0        1.653338    2.014288   -0.000032
      6          7           0       -1.961874   -0.822464    0.000055
      7          7           0        0.239366   -1.879887   -0.000023
```

8	7	0	1.963471	-0.268528	-0.000069
9	7	0	-1.366822	1.263164	0.000057
10	1	0	2.262138	-2.312584	-0.000099
11	6	0	-2.405897	0.438959	0.000077
12	1	0	-3.440775	0.736328	0.000107
13	1	0	-2.556374	-1.645295	0.000063
14	1	0	2.969331	-0.123795	-0.000104
15	1	0	-1.432870	2.275601	0.000070

Result for N+N7H.log

```
SCF Done:  E(RB3LYP) =  -412.444983344     A.U. after    1 cycles
```

```
Zero-point correction=                           0.107954 (Hartree/Particle)
Thermal correction to Energy=                    0.113830
Thermal correction to Enthalpy=                  0.114774
Thermal correction to Gibbs Free Energy=         0.077825
Sum of electronic and zero-point Energies=          -412.337030
Sum of electronic and thermal Energies=             -412.331154
Sum of electronic and thermal Enthalpies=           -412.330210
Sum of electronic and thermal Free Energies=        -412.367158
```

	E (Thermal) KCal/Mol	CV Cal/Mol-Kelvin	S Cal/Mol-Kelvin
Total	71.429	23.718	77.765

Center Number	Atomic Number	Atomic Type	Coordinates (Angstroms) X	Y	Z
1	6	0	0.192316	0.720757	0.000000
2	6	0	0.158470	-0.674398	-0.000000
3	6	0	-2.048694	-0.684776	-0.000000
4	6	0	-1.031931	1.386683	0.000000
5	7	0	1.486260	-1.099729	-0.000000
6	7	0	-0.928745	-1.412604	-0.000000
7	7	0	-2.137045	0.657635	-0.000000
8	7	0	1.540430	1.076997	0.000000
9	6	0	2.281544	-0.029761	0.000000
10	1	0	1.795389	-2.065184	-0.000000
11	1	0	3.359873	-0.054428	0.000001
12	1	0	-1.127952	2.467329	0.000000
13	1	0	-2.984029	-1.230290	-0.000001
14	1	0	1.920193	2.015453	0.000001

Result for N+N7H_water.log

```
SCF Done:  E(RB3LYP) =  -412.559481849     A.U. after    1 cycles
```

```
Zero-point correction=                           0.108281 (Hartree/Particle)
Thermal correction to Energy=                    0.114116
Thermal correction to Enthalpy=                  0.115060
Thermal correction to Gibbs Free Energy=         0.078198
Sum of electronic and zero-point Energies=           -412.451201
Sum of electronic and thermal Energies=              -412.445366
Sum of electronic and thermal Enthalpies=            -412.444422
Sum of electronic and thermal Free Energies=         -412.481284

                    E (Thermal)             CV                S
                     KCal/Mol        Cal/Mol-Kelvin    Cal/Mol-Kelvin
Total                   71.609             23.590             77.582

---------------------------------------------------------------------
Center     Atomic      Atomic             Coordinates (Angstroms)
Number     Number       Type             X           Y           Z
---------------------------------------------------------------------
     1          6           0        0.194171    0.712403    0.000000
     2          6           0        0.165513   -0.682865   -0.000000
     3          6           0       -2.047918   -0.675728   -0.000000
     4          6           0       -1.015390    1.382809    0.000000
     5          7           0        1.483740   -1.101828   -0.000000
     6          7           0       -0.935825   -1.413954   -0.000000
     7          7           0       -2.139209    0.664868   -0.000000
     8          7           0        1.532234    1.076158    0.000000
     9          6           0        2.272037   -0.025062    0.000000
    10          1           0        1.816227   -2.060840   -0.000000
    11          1           0        3.349446   -0.048082    0.000000
    12          1           0       -1.086847    2.462854    0.000000
    13          1           0       -2.983238   -1.218882   -0.000001
    14          1           0        1.907351    2.018898    0.000001
---------------------------------------------------------------------
```

Result for X+N7H.log

```
SCF Done:  E(RB3LYP) =  -562.992090640     A.U. after    1 cycles

Zero-point correction=                           0.117401 (Hartree/Particle)
Thermal correction to Energy=                    0.125633
Thermal correction to Enthalpy=                  0.126577
Thermal correction to Gibbs Free Energy=         0.084090
Sum of electronic and zero-point Energies=           -562.874690
Sum of electronic and thermal Energies=              -562.866457
Sum of electronic and thermal Enthalpies=            -562.865513
Sum of electronic and thermal Free Energies=         -562.908001

                    E (Thermal)             CV                S
                     KCal/Mol        Cal/Mol-Kelvin    Cal/Mol-Kelvin
Total                   78.836             32.300             89.423

---------------------------------------------------------------------
```

```
 Center     Atomic      Atomic             Coordinates (Angstroms)
 Number     Number       Type             X           Y           Z
 ---------------------------------------------------------------------
      1          6           0        0.723209    0.477153    0.000080
      2          6           0        0.506260   -0.872845   -0.000020
      3          6           0       -1.876342   -0.576276    0.000032
      4          6           0       -0.359753    1.446523    0.000276
      5          6           0        2.698453   -0.511650    0.000015
      6          8           0       -0.223471    2.643525    0.000005
      7          8           0       -2.980648   -1.034604   -0.000059
      8          7           0        1.747225   -1.473863   -0.000180
      9          7           0       -0.730263   -1.415461   -0.000033
     10          7           0       -1.596730    0.791176    0.000136
     11          7           0        2.089680    0.663214   -0.000126
     12          1           0        3.761632   -0.684430    0.000029
     13          1           0        1.943830   -2.466841   -0.000318
     14          1           0       -0.919269   -2.408783   -0.000139
     15          1           0       -2.419451    1.386067    0.000129
     16          1           0        2.545859    1.569731   -0.000143
 ---------------------------------------------------------------------
```

Result for X+N7H_water.log

```
 SCF Done:  E(RB3LYP) =  -563.119646638     A.U. after    1 cycles

 Zero-point correction=                           0.117080 (Hartree/Particle)
 Thermal correction to Energy=                    0.125198
 Thermal correction to Enthalpy=                  0.126143
 Thermal correction to Gibbs Free Energy=         0.083954
 Sum of electronic and zero-point Energies=           -563.002567
 Sum of electronic and thermal Energies=              -562.994448
 Sum of electronic and thermal Enthalpies=            -562.993504
 Sum of electronic and thermal Free Energies=         -563.035693

                     E (Thermal)             CV                S
                      KCal/Mol        Cal/Mol-Kelvin    Cal/Mol-Kelvin
 Total                   78.563             32.072             88.794

 ---------------------------------------------------------------------
 Center     Atomic      Atomic             Coordinates (Angstroms)
 Number     Number       Type             X           Y           Z
 ---------------------------------------------------------------------
      1          6           0        0.729483    0.498131   -0.000165
      2          6           0        0.512575   -0.853046   -0.000149
      3          6           0       -1.839824   -0.594339    0.000074
      4          6           0       -0.359582    1.429699   -0.000357
      5          6           0        2.687229   -0.509178   -0.000027
      6          8           0       -0.282893    2.651000    0.000407
      7          8           0       -2.973025   -1.047981    0.000262
      8          7           0        1.736377   -1.462799   -0.000164
      9          7           0       -0.721556   -1.407456   -0.000112
     10          7           0       -1.587533    0.767134    0.000017
     11          7           0        2.100607    0.673774    0.000020
```

12	1	0	3.747970	-0.693167	0.000041
13	1	0	1.918848	-2.461830	-0.000196
14	1	0	-0.864867	-2.411607	-0.000092
15	1	0	-2.415726	1.354988	0.000188
16	1	0	2.586564	1.565286	0.000123
